# DNA display of fragment pairs as a tool for the discovery of novel biologically active small molecules†
†Electronic supplementary information (ESI) available. See DOI: 10.1039/c4sc01654h


**DOI:** 10.1039/c4sc01654h

**Published:** 2014-09-22

**Authors:** J.-P. Daguer, C. Zambaldo, M. Ciobanu, P. Morieux, S. Barluenga, N. Winssinger

**Affiliations:** a Department of Organic Chemistry , NCCR Chemical Biology , University of Geneva , Switzerland . Email: nicolas.winssinger@unige.ch; b Institut de Science et Ingénierie Supramoléculaires (ISIS – UMR 7006) , Université de Strasbourg – CNRS , 8 allée Gaspard Monge , F67000 Strasbourg , France

## Abstract

Fragment-based lead discovery has proven to be a powerful method in the drug discovery process. The combinatorial output that is accessible by combining fragments is very attractive; however, identifying fragment pairs that bind synergistically and linking them productively can be challenging. Several technologies have now been established to prepare and screen nucleic acid-encoded libraries (ssDNA, dsDNA, PNA), and it has been shown that pairs of molecules combined by hybridization can bind synergistically to a target. Herein we apply this concept to combinatorially pair two libraries of small molecule fragments, use the fittest fragments supplemented with closely related analogs to build a focused library covalently linking the fragments with different spacers, and apply this strategy to the discovery of a potent ligand for Hsp70.

## Abstract 2

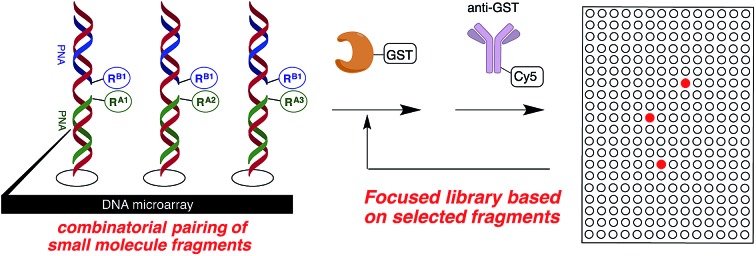
A focused library for Hsp70 was prepared from fragments identified from an array combinatorially pairing two libraries of small molecule fragments. Screening of the focus library yielded high affinity ligand to Hsp70.

## Introduction

**Fig. 1:**
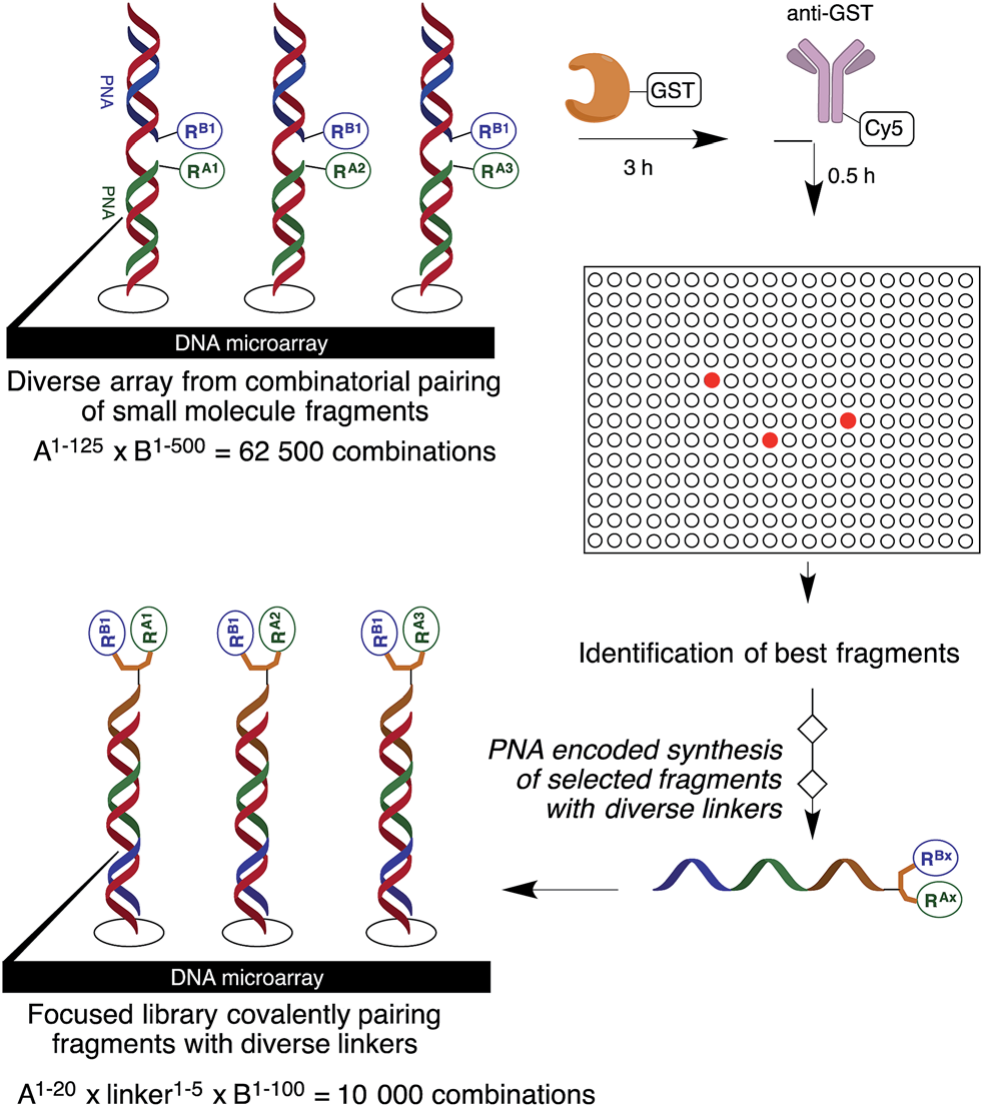
General protocol.

The discovery of small molecules that bind and modulate the activity of a specific target is a cornerstone of chemical biology and the development of new therapeutics. Decades of high-throughput screening to identify small molecule ligands have shown that library size is not everything and that libraries arising from a single chemistry generally suffer from redundancy.^1^ Moreover, many targets cannot be screened in this way because of a lack of measurable enzymatic activity or the lack of a ligand for use in a displacement assay. Fragment-based approaches have proven to be a powerful complementary strategy to high-throughput screening.^2^ The ability to combine different fragments provides rapid access to a large and diverse molecular diversity space. However, identifying fragments that bind synergistically to a target remains challenging. Moreover, finding suitable chemistry to link the fragments can be problematic. In light of these issues, fragment growing coupled with structural information is generally favoured. Despite the success of these technologies, the slow turnaround and high cost associated with the discovery of small molecule ligands is inadequate to interrogate the function of emerging targets.^3^ Several technologies have now been reported to synthesize and screen libraries of DNA- or PNA-encoded small molecules.^4^ Using these technologies, one can rapidly identify binders using an affinity purification of the library against an immobilized target (μg of protein are generally sufficient) followed by a tag decoding (next-generation sequencing or microarray hybridization). Compared to traditional screening, such technologies significantly miniaturize and accelerate the ligand discovery process. Moreover, fragment pairing through complementary sequences or hybridization to a DNA template can be used to identify ligand pairs that interact synergistically with a target.^5^,^6^ Although this latter strategy is attractive in terms of molecular diversity, identifying an appropriate linker to covalently pair the fragments remains a laborious process of trial-and-error optimization. Herein we demonstrate the productivity of taking the results from the fragment screen as the starting point for the synthesis of a focused library (Fig. 1).

As a test bed for this methodology, we selected Hsp70 as a target. This choice was motivated by the current interest in the stress response pathway and the fact that Hsp70 (ref. 7) is an important actor in this pathway, coupled with the challenge of screening this target and the limited number of high-affinity ligands reported. The cellular stress response machinery has attracted significant attention as a productive area for therapeutic intervention. It is well established that this machinery is important in malignant progression to overcome cellular insults such as hypoxia, nutrient deprivation, and the accumulation of mutated and incorrectly folded proteins. Furthermore, a number of oncogenic proteins are dependent on the chaperoning activity of this machinery. The successes achieved with selective inhibitors targeting the nucleotide binding pocket of Hsp90 (ref. 8) have stimulated efforts to target other chaperones. Like Hsp90, Hsp70 is an ATP-dependent chaperone but is less specialized than Hsp90 and works in concert with Hsp40. Compared to Hsp90, the low intrinsic ATPase activity of Hsp70 coupled with its high affinity for ADP (260 nM)^9^ has hampered development of HTS assays. Moreover, the lack of precise structural information has hindered structure-guided inhibitor development, with the first structure with an inhibitor targeting the nucleotide binding site being reported in 2009.^10^

## Results and discussion

**Fig. 2:**
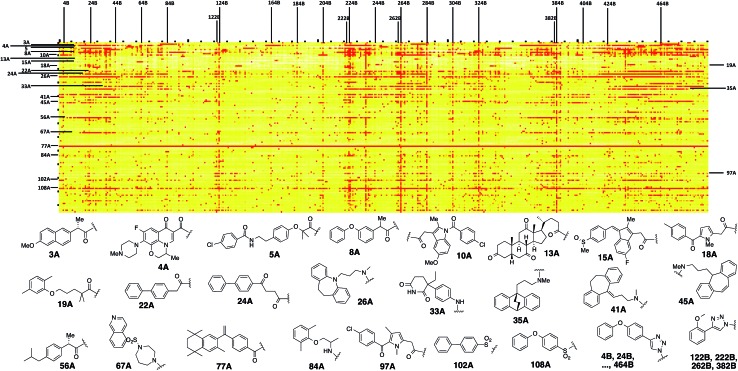
Top: microarray-based screen of combinatorially paired fragments against Hsp70-GST (125 fragment A on the horizontal lines × 500 fragment B on the vertical lines: 62 500 combinations). Bottom: structure of the fragments highlighted on top.

**Scheme 1:**
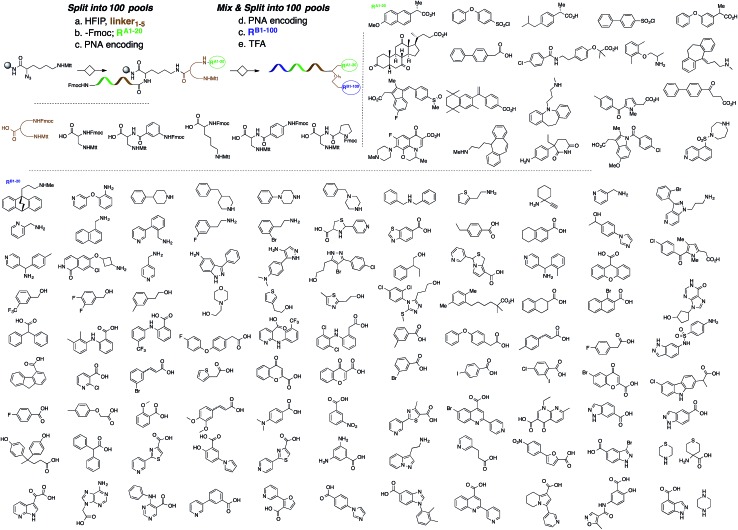
Synthesis of a 10 000-member focused library for Hsp70 (20 A fragments × 5 spacers × 100 B fragments).

**Fig. 3:**
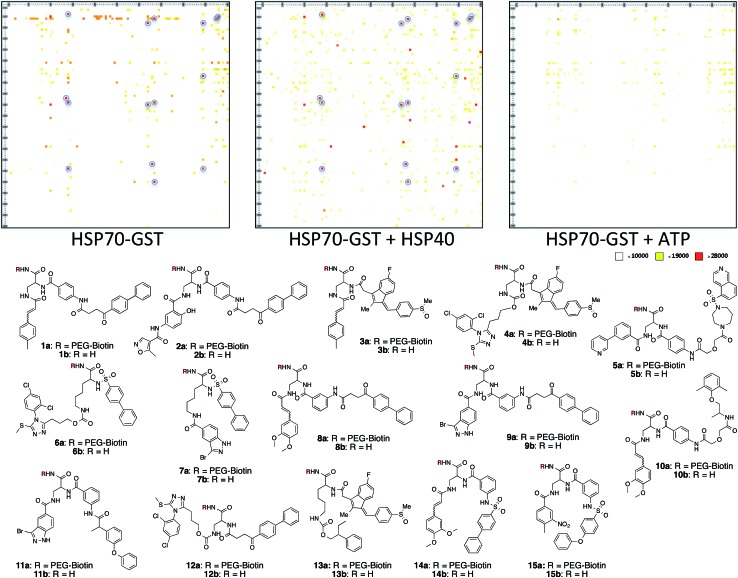
Top: microarray-based screen of the focused library under three different conditions: Hsp70-GST, Hsp70-GST + Hsp40, and Hsp70-GST + ATP (blue circles denote the 15 highest intensity compounds). Bottom: structure of the best ligands (blue circles).

**Fig. 4:**
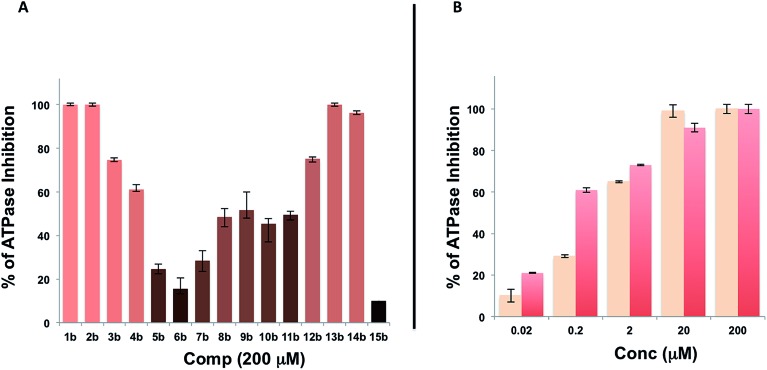
A. Evaluation of the ATPase inhibitor effect of compounds **1b–15b** at 200 mM on Hsp70/Hsp40 (1 μM); B. same as in A for compounds **1b** and **2b** in dose response (0.02–200 molar equivalence).

**Fig. 5:**
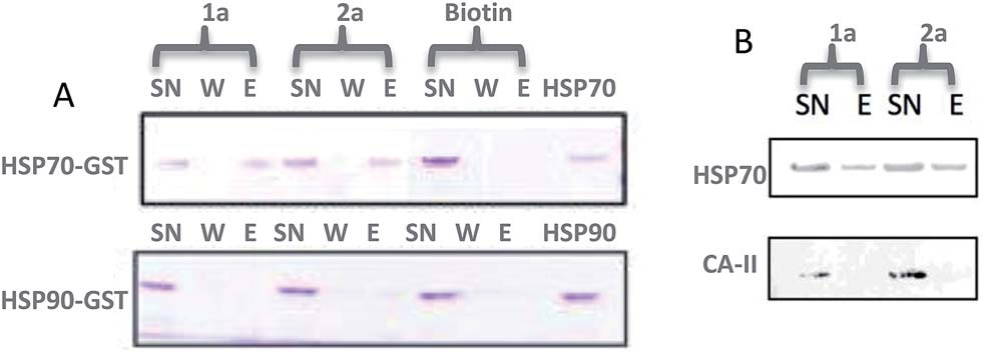
Affinity pull-down of Hsp70 *vs.* related (Hsp90) or unrelated (carbonic anhydrase) proteins using streptavidin resin loaded with compound **1a** or **2a**. A. SDS PAGE (*Coomassie* affinity Brilliant Blue staining) of supernatant (SN) wash and eluent (E) fractions (biotin was used as a negative control); B. same experiment as in A but with crude cell lysates from HEK and Western blotting using specific monoclonal anti-Hsp70 antibody (carbonic anhydrase was used as a negative control; see Table S1† for MS-MS analysis of the eluent of **2a**).

A microarray pairing 125 biologically validated fragments^11^ (FDA-approved drugs, bioactive natural products or fragments thereof) with a library of 500 heterocycles was used as a preliminary screen.^6^ The microarray contained 62 500 unique DNA sequences that combinatorially paired both fragment libraries. The screening was performed by incubating GST-tagged Hsp70 for 3 h, washing the slide to remove unbound Hsp70, and revealing Hsp70 binders using an anti-GST antibody labelled with a Dylight 649 fluorophore.^12^ The result from this screen with Hsp70 is shown in Fig. 2, arranged with fragments from the 125-fragment set and 500-fragment set on horizontal and vertical lines, respectively. The recurrence of binding across a line reflects the fitness of a given fragment. Thus, line intensities were used to rank the different fragments. What is interesting is that lines across the library of the 500-fragment set (4B, 24B, 44B, 84B, 124B, …) shared a common structural motif (biaryl-ether; Fig. 2). This motif was also present in the set of 125 fragments (fragment 108A) and also showed good binding, thus offering a cross-validation from this preliminary screening. It is noteworthy that this biaryl moiety is part of a previously reported Hsp70 ligand (MAL3-101).^13^,^14^ Aside from fragment 77A, all other A fragments showed different affinities depending on their combination with different B fragments, suggesting a synergy of interactions between both fragments and Hsp70. Although screening fragments as conjugates to a nucleic acid tag restricts their binding mode to a particular orientation (the orientation that will position the linker to the tag toward the outside of the binding pocket), it greatly facilitates the synthesis of the corresponding covalent adduct combining both fragments, as the same chemistry that was used to link the fragments to the tag can be used to covalently pair them. Nonetheless, the linker length and conformational bias must be identified experimentally. We reasoned that the identified fragments could be used as the starting point for a focused library covalently pairing the fragments. This would allow for supplementing the selected fragment set with new entities (near neighbours) and enriching the local diversity space around these initial hits. Such iterative processes are highly efficient in sifting through a large diversity space. Following this strategy, we designed an Hsp70-focused library of 10 000 members using 20 fragments A × 5 different linkers with different lengths and geometrical constraints × 100 fragments B. The 100-fragment set contained 90 entities that were not present in the original screen. To be viable, an iterative library synthesis approach should be leveraged on chemistry that is compatible with SPPS and can be performed by automated PNA-encoded synthesis. As shown in Scheme 1, this was achieved through the use of three orthogonal amino group masking strategies (Mtt, Fmoc, and azide) that enabled selective chemical reactions at both sites of the linker joining the fragment and the encoding site. Thus, a resin derivatized with lysine with orthogonal protecting groups (Mtt and N3) was split into 100 pools and deprotected on one end (Mtt deprotection using HFIP), and the different linkers to join both fragments were introduced by standard peptide coupling method, affording the two sites for fragment coupling protected by Fmoc and Mtt, respectively.^15^ The fragments were coupled using two different chemistries: for fragments containing a carboxylic acid, DIC/HOBt was used; for fragments with an amino group, hydroxyl, or aniline, the amino-functionalized linker was first treated with 4-nitrophenylchloroformate and the resulting carbamate coupled with the fragment. The azide was next reduced and the first 100 pools were encoded with a 7mer Boc-protected PNA sequence. The resins were mixed and split to introduce the second encoding 7mer sequence followed by deprotection of Mtt and the introduction of the corresponding second fragments. The success of each fragment coupling and encoding step was verified by MALDI analysis of an analytical sample. Cleavage of the final library and hybridization afforded the spatially resolved 10 000-member Hsp70-focused library. A slide containing 4-fold redundancy of each DNA sequence randomly distributed on the array was used to have four data points for each small molecule–target interaction, thus allowing a statistical analysis of the hits. The array was screened under three different conditions: Hsp70, Hsp70 + Hsp40, and Hsp70 + ATP. It is important to note that hits from the three different screens (the 50 highest intensity spots of the array) had less than 10% standard deviation across the four copies of the molecule. For the purpose of this study, we chose to focus on compounds that were directly or allosterically outcompeted by ATP but not by Hsp40 (highlighted by circles in Fig. 3). The 15 compounds showing the highest fluorescent intensity from this set were selected for resynthesis without the PNA tag as well as replacing the PNA tag with a biotin. Biotin-tagged compounds facilitate affinity measurement by providing simple immobilization to a streptavidin-coated SPR chip. Furthermore, biotin conjugates can be used to assess the selectivity of a selected compound against a proteome by affinity pull-down experiments with lysates. Thus, the affinity of the 15 biotin tagged compounds was measured by SPR using Hsp70 and Hsp90 as related ATP-dependent chaperones for comparison (Table 1). Gratifyingly, all 15 hits from the array were confirmed by SPR to be high-affinity ligands, with *K*_D_ ranging from 0.64 nM to 12.1 nM. One ligand (compound **2a**) showed no measurable affinity for Hsp90 but was an excellent ligand for Hsp70 (1.58 nM); 13 other compounds showed a selectivity for Hsp70 *vs.* Hsp90, ranging from 2- to 95-fold, and one compound appeared to be a marginally better ligand for Hsp90 than Hsp70 (compound **11a**, 12 nM for Hsp70 *vs.* 5.6 nM for Hsp90). In light of artefacts that can arise from affinity measurements of molecules immobilized on surfaces, we next prepared a fluorescein isothiourea conjugate of **1** (**1-FITC**) for anisotropy measurements. Using a displacement assay, an affinity of 31 nM for **1b** was measured (see Fig. S1† for titration plots). These affinities are notable considering that the tightest Hsp70 binder reported to date has an affinity of 210 nM.^16^ We next assessed the ATPase inhibition of the selected compounds lacking the biotin tag (**1a–15a**). Note that Hsp70 ligands reported thus far bind to different domains of Hsp70 (nucleotide binding pocket,^10^ substrate binding site,^17^ C-terminal EEVD motif,^18^ as well as an allosteric site^19^) and do not all inhibit its ATPase activity; some even stimulate this activity (such as MAL3-101).^14^ The high concentration of Hsp70 (1 μM) required to reliably measure ATP hydrolysis precludes the identification of sub-micromolar EC_50_. At 200 molar equivalence of inhibitor (200 μM), 11 out of 15 compounds showed greater than 50% inhibition, with compounds **1b** and **2b** showing complete inhibition. Compounds **1b** and **2b** were further tested in a dose–response manner and both compounds showed greater than 50% inhibition using 1 molar equivalent to Hsp70 (Fig. 4B). We then verified the ability of compounds **1a** and **2a** to capture Hsp70 using streptavidin magnetic beads. As shown in Fig. 5, both compounds retained Hsp70 but not Hsp90, in line with affinity measurements by SPR. It is important to note that neither of the fragments used in the synthesis of **1a** and **2a** retained Hsp70 under the same conditions, attesting to the synergic interaction of the fragments and linker (Fig. S2†). Furthermore, the pull down of Hsp70 with immobilized **1a** was outcompeted with soluble free ligand in solution (Fig. S2†). When crude cell lysates were used, both compounds retained Hsp70 but no carbonic anhydrase (used as a negative control). To further assess the selectivity of the compound against a crude proteome, we challenged the immobilized compound with crude lysates from HEK cells, washed it, and subjected the retained fraction to a proteomic analysis. Hsp70 was identified as having the highest sequence coverage along with Hsp72, a stress-induced isoform of constitutively expressed Hsp70 (see Table S1† for list of retained proteins).^20^

**Table 1:** SPR affinity measurements of compounds immobilized on a streptavidin chip for the selected ligand. Fluorescence intensities of compounds on the microarray (the mean of four spots with a standard deviation between 5% and 10%)

Compound	Codons	FU microarray HSP70-GST	*K*_D_ HSP70 (nM)	*K*_D_ HSP90 (nM)
**1a**	6–67	28 333	5.94	563
**2a**	6–95	28 653	1.58	Low binding
**3a**	44–67	28 945	7.65	27
**4a**	44–28	28 566	2.69	10.7
**5a**	5–96	28 447	0.38	(5.44)^*a*^
**6a**	74–28	28 958	2.33	18.7
**7a**	74–89	24 143	1.34	(3.17)^*a*^
**8a**	8–64	27 982	(0.80)^*a*^	(16.9)^*a*^
**9a**	8–89	29 024	(0.64)^*a*^	(14.7)^*a*^
**10a**	45–64	28 961	1.88	2.97
**11a**	32–89	27 941	12.10	(5.6)^*a*^
**12a**	4–28	28 174	2.02	10.1
**13a**	42–27	27 215	(1.16)^*a*^	(29.5)^*a*^
**14a**	80–64	28 588	2.88	(52.8)^*a*^
**15a**	72–66	28 732	(1.30)^*a*^	(35.3)^*a*^

^*a*^ *χ* ^2^ > 10.

## Conclusions

The study presented herein illustrates an efficient workflow for identifying potent small molecule ligands from diverse fragment libraries. A key to the success of this workflow is the ability to generate a focused library upon the identification of small molecule fragments from generic libraries. Salient features are the opportunity to enrich the focused library with new (near neighbour) fragments and the use of robust and streamlined synthetic technologies to pair fragments covalently. The use of a combinatorial approach to optimize the covalent pairing offers the potential to adjust distance and geometry of the linker. Different linkers may also provide additional affinity through target-linker interactions. The research reported herein led to the identification of the most potent Hsp70 ligand reported to date. The ligand is selective for Hsp70 s (Hsp70/Hsp72) across a proteome.

## Supplementary Material

Supplementary information
