# Supplementary material for: DNA display of fragment pairs as a tool for the discovery of novel biologically active small molecules
Source: Chem Sci. 2014 Sep 22;6(1):739–44. doi: 10.1039/c4sc01654h (PMC6085657; doi:10.1039/c4sc01654h)
Supplement: Supplementary file 1 [file SC-006-C4SC01654H-s001.pdf]

Electronic Supplementary Material (ESI) for Chemical Science.
This journal is © The Royal Society of Chemistry 2014

Supplementary Information

Figure S1 p 2

Figure S2 p 3

Table S1 p 4

1. General Techniques for chemical synthesis p 5
2. General procedures for the supported synthesis of PNA-encoded libraries p 6
3. Screening of combinatorial fragment pair with Hsp70-GST p 7
4. Synthesis and characterization of 10 000 member focused library p 8
5. Screening of the focused library p 29
6. Characterization of selected binders p 30
7. SPR and BLI affinity measurements of 1-15 p 47
8. Protein pull-down with immobilized ligand p 58
9. Hsp70 ATPase activity measures p 58
10. Pull down of Hsp70 from crude cell extracts p 59

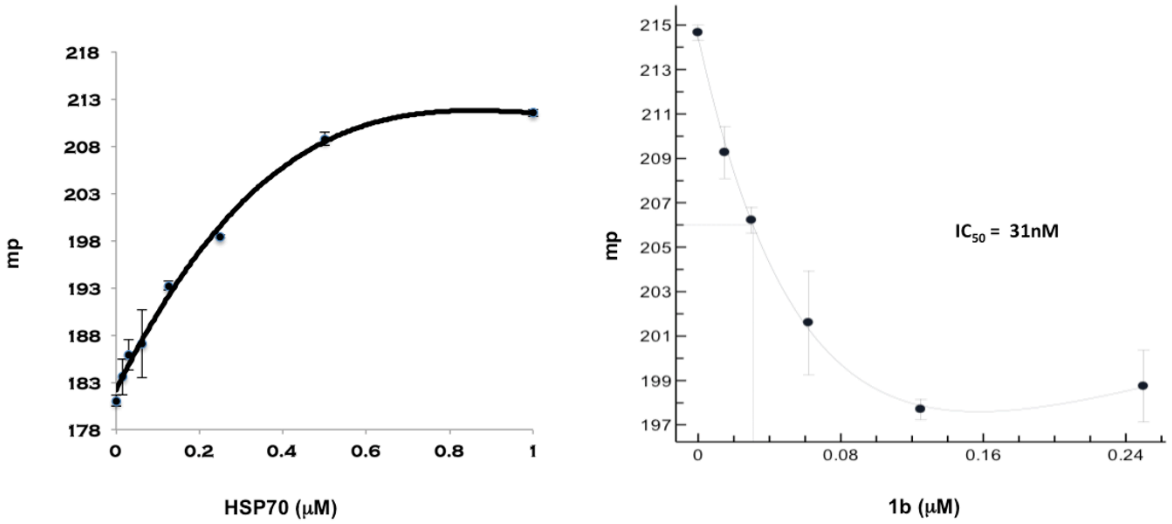

Figure S1. Fluorescence anisotropy measurements. Left: Titration 1-FITC with Hsp70 measured by fluorescence polarization. Serial dilution of Hsp70 were incubated with **1-FITC** (25 nM) for 30 min at room temperature and FP values were measured. Buffer (25 mM HEPES, pH 7.4 – 50 mM KCl – 20 mM NaCl – 5 mM $MgCl_2$ - 100 mM $CaCl_2$ – 0.05% Tween 20 – 2% DMSO) Background correction using the protein dilutions. Each data point is the average of three independent experiments; Right: Affinity of **1b** for Hsp70 by fluorescence polarization: Serial dilution of **1b** + **1-FITC** (25 nM) with Hsp70 (100 nM). Buffer (25 mM HEPES, pH 7.4 – 50 mM KCl – 20 mM NaCl – 5 mM $MgCl_2$ - 100 mM $CaCl_2$ – 0.05% Tween 20 – 2% DMSO) Each data point is the average of three independent experiments. IC_{50} = 31 nM (R^2 = 0.998).

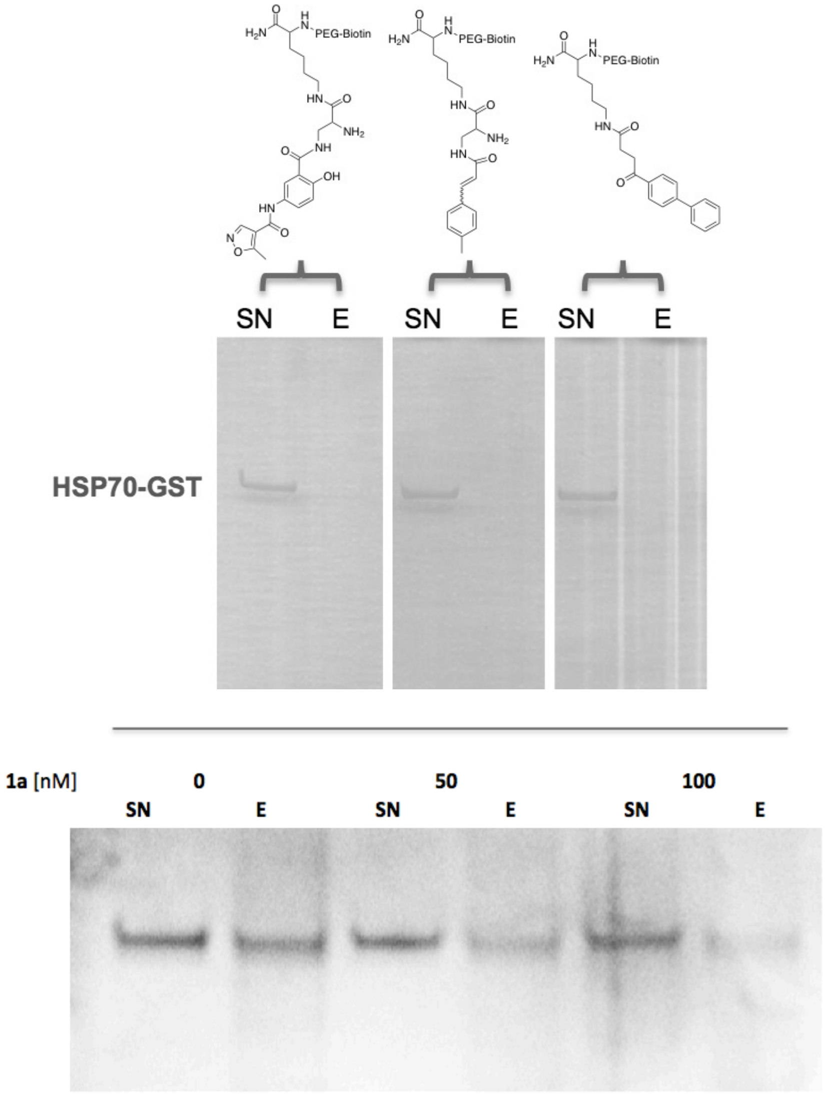

Figure S1. Top: *Coomassie* Brilliant Blue staining SDS PAGE separation of a pull down of Hsp70-GST with fragments of **1a** and **2a** fragments (SN stands for supernatant, E for elution); Bottom: Hsp70 pull-down on Streptavidin beads functionalized with 1a (biotin conjugate) in competition with different concentrations of 1b. Buffer (25 mM HEPES, pH 7.4 – 0.5 M KCl – 0.2 M NaCl – 0.02 M $MgCl_2$ - 100 mM $CaCl_2$ – 0.05% Tween 20) Elution on SDS and SDS PAGE analysis.

Table S1. List of Proteins identified in a pull down with immobilized compound **2** from a HEK crude cell lysate.

MSMS	Protein Description in UniPro	Mw (kDa)	Protein pI	N°Access	Protein Score Mascot	Error (ppm)[a]	% Protein Coverage[b]
16	Heat shock 70 kDa protein 1A/1B OS=Homo sapiens GN=HspA1A PE=1 SV=5	70.0	5.4	Hsp71_HUMAN	881.3	5.54	24.8
15	Tubulin beta chain OS=Homo sapiens GN=TUBB PE=1 SV=2	49.6	4.6	TBB5_HUMAN	764.2	5.58	26.4
13	Tubulin beta-4B chain OS=Homo sapiens GN=TUBB4B PE=1 SV=1	49.8	4.6	TBB4B_HUMAN	647.7	5.85	26.3
10	Actin, cytoplasmic 1 OS=Homo sapiens GN=ACTB PE=1 SV=1	41.7	5.2	ACTB_HUMAN	411.7	2.33	26.7
9	Tubulin alpha-1C chain OS=Homo sapiens GN=TUBA1C PE=1 SV=1	49.9	4.8	TBA1C_HUMAN	487.0	5.11	25.2
8	60 kDa heat shock protein, mitochondrial OS=Homo sapiens GN=HspD1 PE=1 SV=2	61.0	5.6	CH60_HUMAN	400.6	6.81	13.6
7	Putative tubulin beta chain-like protein ENSP00000290377 OS=Homo sapiens PE=5 SV=2	41.7	4.6	YI016_HUMAN	199.1	5.51	9.9
6	Heat shock protein 70 2 OS=Pichia angusta GN=HSA2 PE=3 SV=2	70.0	4.8	Hsp72_PICAN	321.3	7.54	7.9
4	Actin, cytoplasmic 1 OS=Homo sapiens GN=ACTB PE=1 SV=1	41.7	5.2	ACTB_HUMAN	154.7	9.67	8.8
3	Ig gamma-1 chain C region OS=Homo sapiens GN=IGHG1 PE=1 SV=1	36.1	9.4	IGHG1_HUMAN	105.7	7.65	4.5
3	78 kDa glucose-regulated protein OS=Homo sapiens GN=HspA5 PE=1 SV=2	72.3	4.9	GRP78_HUMAN	137.4	5.85	4.3
3	WD repeat, SAM and U-box domain-containing protein 1 OS=Homo sapiens GN=WDSUB1 PE=1 SV=3	52.8	5.9	WSDU1_HUMAN	61.7	0.77	1.3
2	Elongation factor 1-alpha 1 OS=Homo sapiens GN=EEF1A1 PE=1 SV=1	50.1	9.7	EF1A1_HUMAN	87.3	7.45	4.1
2	RuvB-like 1 OS=Homo sapiens GN=RUVBL1 PE=1 SV=1	50.2	6.0	RUVB1_HUMAN	105.6	7.61	5.0
1	RuvB-like 2 OS=Homo sapiens GN=RUVBL2 PE=1 SV=3	51.1	5.4	RUVB2_HUMAN	57.2	6.46	2.2
1	Tubulin alpha-3E chain OS=Homo sapiens GN=TUBA3E PE=1 SV=2	49.8	4.9	TBA3E_HUMAN	54.1	0.13	3.1
1	Far upstream element-binding protein 2 OS=Homo sapiens GN=KHSRP PE=1 SV=4	73.1	7.0	FUBP2_HUMAN	83.9	7.41	1.5

[a]The error in ppm is calculated between the experimental and the theoretical mass of the peptides.
[b]The protein coverage is calculated on the basis of the amino acids identified and matched to the total number of aa in the protein sequence.

1. General Techniques for chemical synthesis

All reactions were carried out under a nitrogen atmosphere with dry solvents under anhydrous conditions, unless otherwise noted. Anhydrous solvents were obtained by passing through commercially available alumina columns (Innovative Technology, Inc.). NovaPEG Rink Amide Resin was purchased from Novabiochem®, and was swollen in DCM before each reaction. Solid phase reactions were carried in SPE tubes fitted with a frit and a tap. Automated solid phase synthesis was carried out on an Intavis AG Multipep RS instrument. LC-MS spectras were recorded using an HP1100 series of Thermo Electron Corporation HPLC with a Thermo Finnigan Surveyor MSQ Mass Spectrometer System. A Thermo Scientific column (50 x 2.1 mm) was used. MALDI spectra were measured using a Brucker Daltonics Autoflex TOF spectrometer. Cleavages were carried out on 0.1-0.3 mg of dry resin with 20 µL of TFA for 1 h at room temperature. The TFA solution was either evaporated or added to 200 µL of Et_2O and centrifuged at 15000g for 5 min to pellet the precipitated compound. The resulting pellet was then washed with Et_2O (200 µL) and dissolved in 1:1 MeCN:H_2O (40µL) for analysis. The mix and split synthesis was performed according to previously established protocols.[1]

2. General procedures for the supported synthesis of PNA-encoded libraries

Procedure 1. Capping. To 100 mg of NovaPEG Rink amide resin were added 2.0 mL of capping mixture (9.2 mL of acetic anhydride and 13 mL of 2,6-lutidine in 188 mL of DMF) and the resin was shaken for 15 min. Subsequently, the resin was washed with 6 x 2 mL of DMF and 6 x 2 mL of CH_2Cl_2.

Procedure 2. Capping in Intavis AG Multipep RS Synthesizer. To 10 mg of NovaPEG Rink amide resin were added 100 µL of capping mixture (9.2 mL of acetic anhydride and 13 mL of 2,6 lutidine in 188 mL of DMF). After 5 min, the resin was washed with 2 x 250 µL of DMF.

Procedure 3. Fmoc deprotection. To 100 mg of NovaPEG Rink amide resin were added 2.0 mL of 20% piperidine solution in DMF, and the resin was shaken for 5 min. Subsequently, the resin was washed with 6 x 2 mL of DMF and 6 x 2 mL of CH_2Cl_2, and the deprotection sequence was repeated a second time.

Procedure 4. Fmoc deprotection in Intavis AG Multipep RS Synthesizer. To 10 mg of NovaPEG Rink amide resin were added 100 µL of 20% piperidine solution in DMF. After 2 min, the resin was washed with 250 µL DMF and the sequence was repeated a second time for 4 min. Finally, the resin was washed with 5 x 250 µL of DMF and 3 x 250 µL of CH_2Cl_2.

Procedure 5. First amino acid coupling on resin with loading reduction. To a solution of 0.09 mmol (1.0 equiv, 0.2 mmol/g loading) of amino acid in 7.0 mL of NMP were added 68.9 mg (0.45 mmol, 5.0 equiv) of HOBt followed by 210 µL (1.35 mmol, 15.0 equiv) of DIC. The mixture was activated for 5 min at room temperature, and then added to 450 mg of NovaPEG Rink amide resin. The reaction mixture was shaken for 16 hours and the resin was subsequently washed with 6 x 10 mL of DMF and 6 x 10 mL of CH_2Cl_2. The remaining free amino groups were capped as described in ***procedure 1*** (30 min.)

Procedure 6. Carboxylic acid coupling (including amino acids or PEG-spacer) **using HCTU/HATU activation:** To a solution of 0.08 mmol (4.0 equiv) of amino acid in NMP (1 mL) were added 140 µL (0.07 mmol, 3.5 equiv) of HCTU or HATU (0.5 M) in NMP followed by 67 µL of base solution [DIPEA 1.2 M (0.25 mmol, 4.0 equiv) and 2,6 lutidine 1.8M (0.38 mmol, 6.0 equiv) in NMP]. The mixture was activated for 5 min at room temperature, and then added to 100 mg of resin (0.02 mmol, 1.0 equiv). The reaction mixture was shaken for 2 hours and the resin was subsequently washed with 6 x DMF and 6 x CH_2Cl_2.

Carboxylic acid coupling (including aminoacids or PEG-spacer) **using HOBt/DIC activation**:

The corresponding carboxylic acid (0.01 mmol, 5.0 equiv) was dissolved in 200µL of NMP and HOBt (1.5 mg, 0.01 mmol, 5.0 equiv) followed by DIC (4.7 µL, 0.03 mmol, 15.0 equiv) were added. The mixture was

[1] F. Debaene, J. DaSilva, Z. Pianowski, F. Duran, N. Winssinger, *Tetrahedron* 2007, **63**, 6577-6586

activated for 15 min and then, added to the resin (10 mg, 0.002 mmol, 1.0 equiv) and the reaction was shaken for 12 hours. Finally, the resin was washed with 6 x 250 µL of DMF and 6 x 250 µL CH_2Cl_2.

Procedure 7. Carboxylic acid coupling (including PEG spacer) in Intavis AG Multipep RS Synthesizer. To a solution of 8.0 µmol (4.0 equiv) of carboxylic acid in 40 µL of NMP were added 14 µL (7.0 µmol, 3.5 equiv) of HCTU or HATU 0.5 M in NMP, followed by 6.7 µL of base solution [DIPEA 1.2 M (0.008 mmol, 4.0 equiv) and 2,6 lutidine 1.8 M (0.012 mmol, 6.0 equiv) in NMP]. The mixture was then added to 10 mg (2.0 µmol, 1.0 equiv) of the corresponding resin. After 20 min, the resin was filtered and washed with DMF and the sequence was repeated. The resin was then filtered and washed with 6 x 250 µL of DMF and 6 x 250 µL of CH_2Cl_2. Finally, the resin was capped (***procedure 2***).

Procedure 8. Azide generation. To a solution of 294 mg (1.4 mmol, 11 equiv) of imidazole-1-sulfonyl azide hydrochloride[2] in 12.6 mL of MeOH were added successively 305 mg (2.2 mmol, 18 equiv) of K_2CO_3 and 8.0 mg (0.05 mmol, 0.35 equiv) of anhydrous $CuSO_4$ and the resulting solution was sonicated for 20 min. To 10 mg (2.0 µmol, 1.0 equiv) of the corresponding resin were added 250 µL of the previously prepared solution. After 16 hours, the resin was washed with 6 x 250 µL of sodium diethyl dithiocarbamate 0.02 M in DMF, 6 x 250 µL of DMF, 6 x MeOH and 6 x CH_2Cl_2, and the sequence was repeated.

Procedure 9. Mtt deprotection (also in Intavis AG Multipep RS Synthesizer). 10 mg of resin were treated with 200 µL of a HOBt solution (122 mg in 10 mL of a 1:1 (v:v) solution of hexafluoroisopropanol : 1,2 dichoroethane) for 3 min. The sequence was repeated 4 times with a CH_2Cl_2 wash after the second cycle.

Procedure 10.* PNA synthesis in Intavis AG Multipep RS Synthesizer.** Fmoc or Mtt were removed as described in ***procedure 3 and ***9*** respectively. Then, to a solution of 8.0 µmol (4.0 equiv) of the corresponding PNA monomer (the nucleobases are Boc protected[3]) in 40 µL of NMP were added 14 µL (7.0 µmol, 3.5 equiv) of HCTU or HATU 0.5 M in NMP (for Mtt monomers, use 21.3 µL (7.0 µmol, 3.5 equiv) of TNTU 0.33 M in NMP), followed by 6.7 µL of base solution [DIPEA 1.2 M (0.008 mmol, 4.0 equiv) and 2,6-lutidine 1.8M (0.012 mmol, 6.0 equiv) in NMP]. The mixture was then added to 10 mg of the corresponding resin. After 20 min the resin was filtered and washed with DMF and the sequence was repeated, then, the resin was washed with 6 x 100 µL of DMF and 6 x 100 µL of CH_2Cl_2. Finally, the resin was capped (***procedure 2***).

Procedure 11. Cleavage from the resin. 10 mg of resin were treated with 200 µL TFA for 4 hours. Next, the resulting solution was precipitated in 2 mL of Et_2O and centrifuged to recover the product as a pellet. The precipitate was re-dissolved in 500 µL of H_2O, the resulting was solution was filtered and then freeze-dried.

Procedure 12. General procedure for copper catalyzed cycloaddition.[4] To the corresponding resin (11.5 mg) were added successively 173 µL (0.0173 mmol, 7.5 equiv) of alkyne 0.1M in NMP, 17.3 µL (17.2 µmol, 7.5 equiv) of sodium ascorbate 198 mg/mL in H_2O, 4.4 µL (0.57 µmol, 0.25 equiv) of copper sulfate 21.4 mg/mL in H_2O and 44 µL (1.1 mmol, 0.5 equiv of TBTA). After 16 hours, the resin was washed with 6 x 250 µL of sodium diethyl dithiocarbamate 0.02 M in DMF, 6 x 250 µL of DMF, 6 x MeOH and 6 x CH_2Cl_2.

Procedure 13. Azide reduction. 10 mg of resin were treated with 200µL of a solution of tris 2-carboxyethyl phosphine (0.35 M in 9-1 DMF-H_2O) for 2h min. Finally, the resin was washed with 6 x 250 µL H_2O, 6 x 250 µL DMF and 6 x 250 µL CH_2Cl_2.

Procedure 14. Biotin labelling. 10 mg of resin (0.002 mmol, 1.0 equiv) were treated with a solution of 3.4 mg (0.01 mmol, 5.0 equiv) of Biotin-OSu and 3.3 µL (0.02 mmol, 10.0 equiv) of DIPEA in 100 µL of NMP

[2] E.D. Goddard-Borger, R.V. Stick, *Org. Lett.* 2007, **9**, 3797-3800
[3] S. Pothukanuri, Z. Pianowski, N. Winssinger, *Eur. J. Org. Chem.* 2008, **18**, 3141-3148
[4] N.G. Angelo, P.S. Arora, *J. Org. Chem.* **2007**, *72*, 7963-7967

and 20 µL of DMSO. The reaction was shaken overnight at 50°C and then washed 6 x 250 µL of DMF and 6 x 250 µL of CH_2Cl_2.

3. Screening of combinatorial fragment pair with Hsp70-GST.

Microarrays containing the 62 500 different 28mer DNA sequences complementary to all the combinations of a 500 membered PNA encoded with a 125 membered PNA encoded library were obtained from Agilent (Agilent Technologies, Earray Design ID: 031552; 2x105K format). The small PNA-encoded libraries were previously reported.[5] Both PNA encoded libraries were hybridized on the microarray at an equimolar concentration of 5µM, overnight at 50° C, in PBS buffer, 40 % formamide using the incubation Agilent incubation chamber and incubation oven with rotor for mixing the microarray solution. The slides were removed from the oven, allowed to cool to room temperature and washed twice with PBS-T and once with mQ water to eliminate unhybridized material.

A 100nM solution of recombinant Hsp70-GST protein (Signal Chem, # H34-54G) in HEPES buffer (200 mM HEPES pH 7.4, 0.5 M KCl, 50 mM $MgCl_2$, 200 mM Na_2MO_4, 0.1% Tergitol, 2 mM DTT) was incubated on the array for 2 hours at room temperature with gentle rotation. After, the slide was washed twice with PBS-T and once with water before being dried by centrifugation (5 min at 1000 g).

The slide was then incubated with anti GST antibody DyLigth Tm 649 conjugated (Rockland Immunogenics Cat# 600 443 200) for 20 min and then washed and dried as for Hsp70. The slide was scanned at 635 nm (670DF40 filter) on a Genepix Personnal Scanner 4100A and the fluorescence signal quantified using GenepixPRO7 software.

4. Synthesis of 10 000 member focused library.

<u>Introduction of the spacers & Fragments A</u>

NovaPEG resin was loaded with Fmoc-Lys(Mtt)-OH according to ***procedure 5***, then Fmoc was deprotected according to ***procedure 3*** and the resulting free amine was converted into the corresponding azide as described in ***procedure 8***. Next, the resin was split in 100 pools and the 5 different spacers (bearing a carboxylic acid and two orthogonally protected amines) were introduced through automated synthesis (***procedure 7***) in 20 pools each. After Fmoc deprotection (***procedure 3***), the 20 different **fragments A** were introduced (A_{1-20} for each spacer). These fragments displayed a carboxylic acid, an alcohol an amine or a sulfonyl chloride.

Carboxylic acid coupling was performed according to ***procedure 6***.

Amine coupling via chloroformate activation. 4-nitrophenyl chloroformate (3.2 mg, 0.016 mmol, 8.0 equiv) and 2,6-lutidine (3.8 µL, 0.032 mmol, 16.0 equiv) were dissolved in 200 µL of 1,2-dichloroethane (solution A). 4-DMAP (2 mg, 0.016 mmol, 8 equiv) was dissolved in 28 µL of 1,2-dichloroethane (solution B). Solution A, followed by solution B were added to the corresponding resin (10 mg, 0.002 mmol, 1.0 equiv), and the reaction was shaken for 16 hours. The resin was subsequently washed with 1,2-dichloroethane, and the activation procedure was repeated a second time. Finally, the resin was washed with DMF and CH_2Cl_2. The alcohol or amine (0.07 mmol, 35.0 equiv), followed by DIPEA (23.1 µL, 0.14 mmol, 70.0 equiv – only for amine hydrochlorides) and DMAP (24.4 mg, 0.20 mmol, 100.0 equiv) were dissolved in 280 µL of 1,2-dichloroethane. Then, the solution was added to the corresponding resin (10

[5] J. P. Daguer, M. Ciobanu, S. Alvarez, S. Barluenga, N. Winssinger, *Chem. Sci.* **2011**, *2*, 625-632

mg, 0.002 mmol, 1.0 equiv), and the reaction was shaken for 16 hours at 50°C. Finally, the resin was washed with 6 x 250 µL of DMF and 6 x 250 µL of CH_2Cl_2.

Amine coupling via diglycolic anhydride Diglycolic anhydride (2.3 mg, 0.02 mmol, 10.0 equiv) and 2,6-lutidine (4.7 µL, 0.04 mmol, 20.0 equiv) were dissolved in 200 µL NMP. The solution was added to the resin (10 mg, 0.002 mmol, 1.0 equiv) and the reaction was shaken for 16 hours. Finally, the resin was washed with DMF and CH_2Cl_2. BOP (7.1 mg, 0.016 mmol, 8.0 equiv), HOBt (2.5 mg, 0.016 mmol, 8.0 equiv) and DIPEA (4.2 µL, 0.024 mmol, 12.0 equiv) were dissolved in 100 µL NMP. The solution was added to the corresponding resin and the reaction was shaken for 20 min. Amine or alcohol (0.02 mmol, 10.0 equiv), followed by DIPEA (6.7 µL, 0.04 mmol, 20.0 equiv – only for amine hydrochlorides) and DMAP (4.9 mg, 0.040 mmol, 20 equiv) were dissolved in 100 µL NMP. The solution was subsequently added to the resin, after removing the activation solution, and the reaction was shaken for 16h at 35°C. Finally, the resin was washed with DMF and CH_2Cl_2.

Sulfonyl chlorides coupling The resins were treated with a solution of the corresponding sulfonyl chloride (0.02 mmol, 10.0 equiv) and DIPEA (0.04 mmol, 20.0 equiv) in 200µL of NMP. The reaction was shaken for 8 hours. Finally, the resin was washed with DMF and CH_2Cl_2.

Finally, the azide was reduced (***procedure 13***) and a different 7-mer PNA was introduced in each of the 100 pools by automated synthesis (***procedure 10***), to encode for the 5 spacers x 20 fragments A = 100. TFA cleavage of an aliquot following ***procedure 11*** and MALDI analysis of each pool confirmed the structures presence of each compound and the conversion of the reaction. MALDI spectra (2,5-Dihydroxybenzoic acid or a-Cyano-4-hydroxycinnamic acid matrix and desorbed with laser between 15-55%) were recorded form 700 to 5000 m/z.

Codon 1	Spacer	Fragment A	PNA sequence	Mass expected	Maldi
1	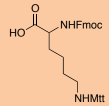	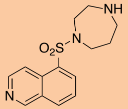	PEG-GGAAGCT	2970.63	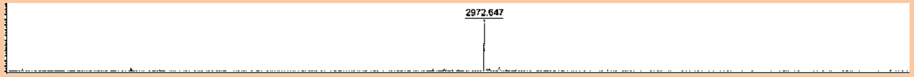
2	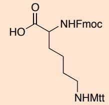	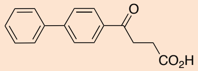	PEG-GTAAGGG	2857.47	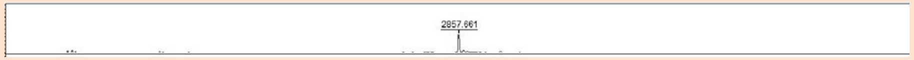
3	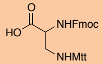	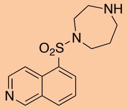	PEG-GTAACCG	2888.62	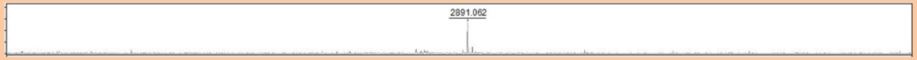
4	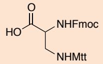	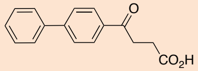	PEG-GTAAGCC	2735.46	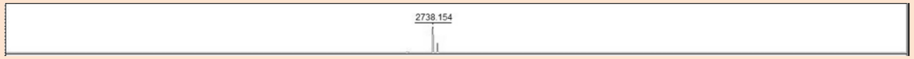
5	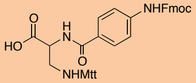	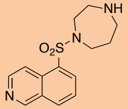	PEG-GCAACGA	3016.75	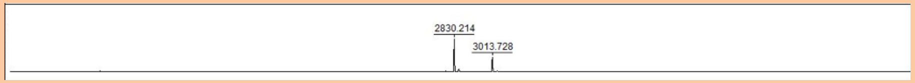
6	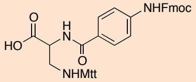	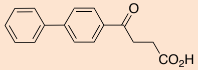	PEG-GCAAGAG	2903.60	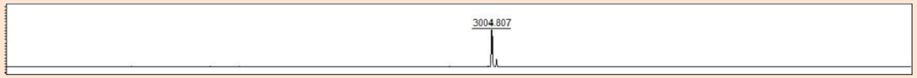
7	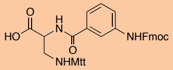	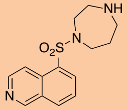	PEG-GCAACTG	3007.74	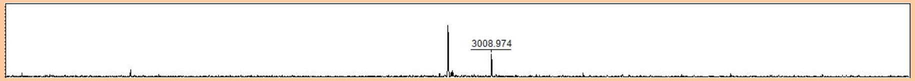

**9**	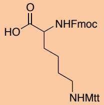	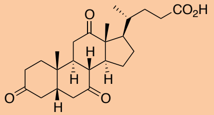	PEG-GCAACCT	2885.69	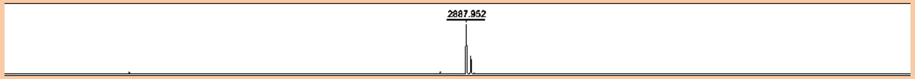
**10**	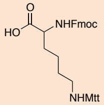	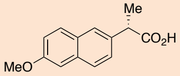	PEG-GCAAGTC	2753.44	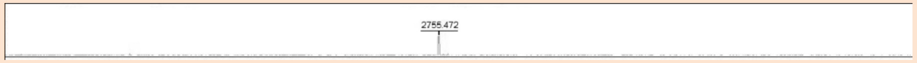
**11**	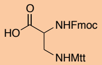	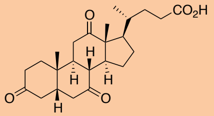	PEG-GAGAGCA	2932.72	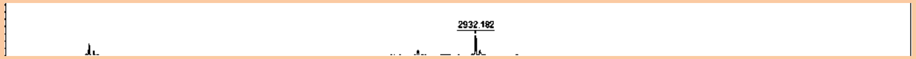
**12**	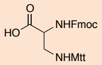	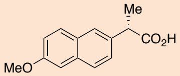	PEG-GGGAGTA	2791.45	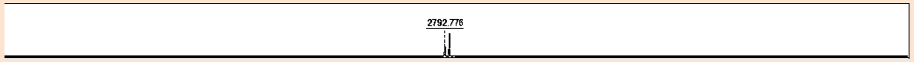
**13**	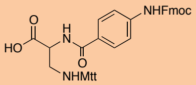	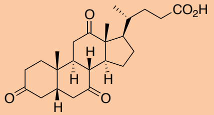	PEG-GTGAGGA	3082.83	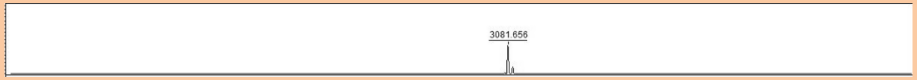
**14**	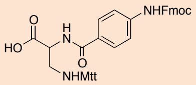	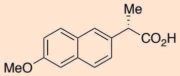	PEG-GTGACCA	2830.56	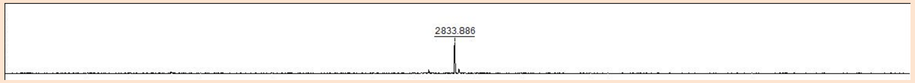
**15**	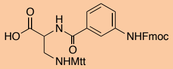	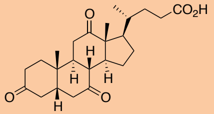	PEG-GTGAGTG	3073.82	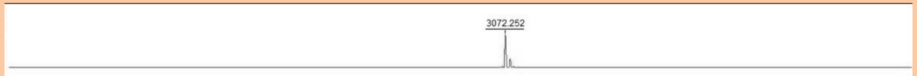
**16**	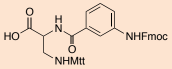	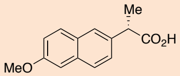	PEG-GTGAGCT	2861.56	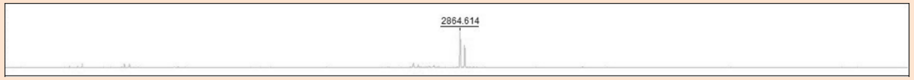
**17**	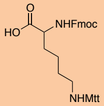	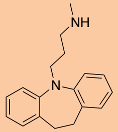	PEG-GCGAGAA	2954.71	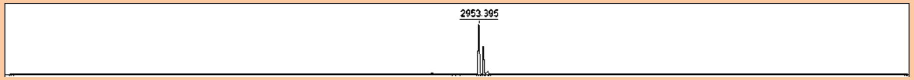
**18**	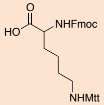	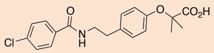	PEG-GCGACTA	2885.00	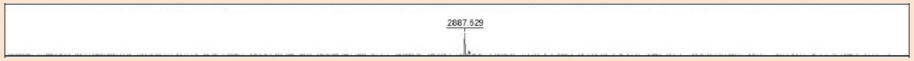

19	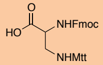	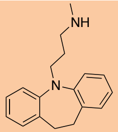	PEG-GCGAGTT	2894.69	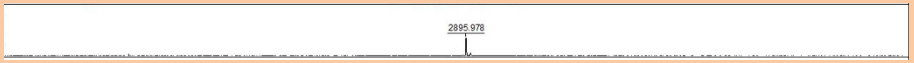
20	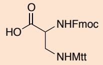	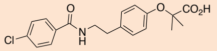	PEG-GGTAGCA	2883.01	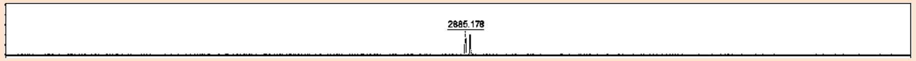
21	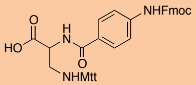	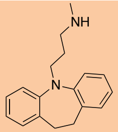	PEG-GTTAGCG	3013.81	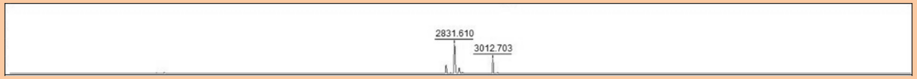
22	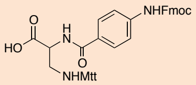	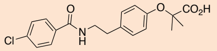	PEG-GCTAGGA	3002.13	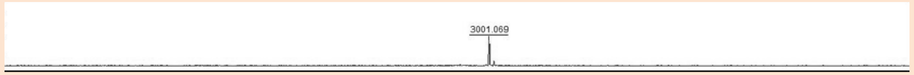
23	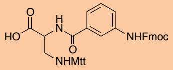	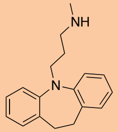	PEG-GCTACCA	2942.80	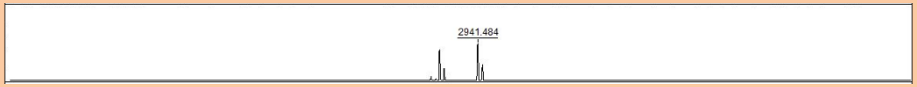
24	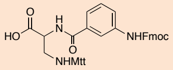	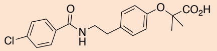	PEG-GCTAGTG	2993.12	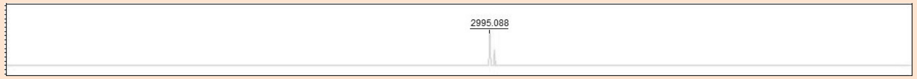
25	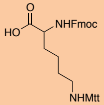	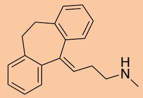	PEG-GCTAGCT	2893.58	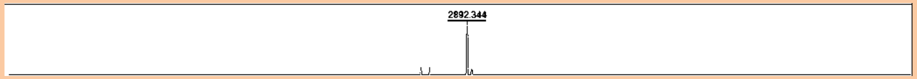
26	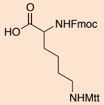	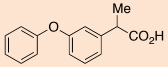	PEG-GACACAC	2734.46	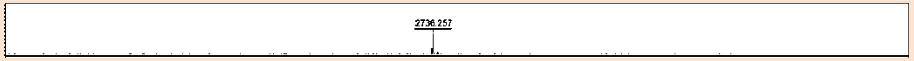
27	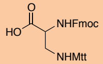	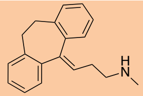	PEG-GTCAGCA	2860.59	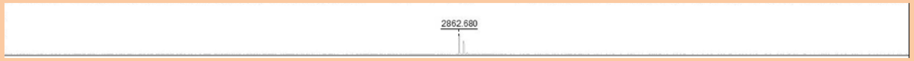
28	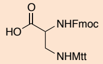	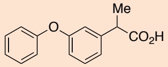	PEG-GCCAGTA	2723.45	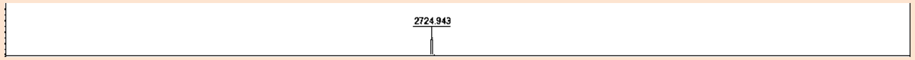

29	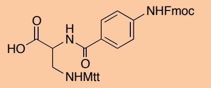	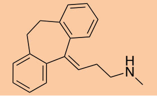	PEG-GGAAAGG	3068.73	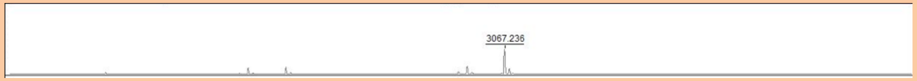
30	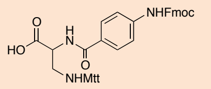	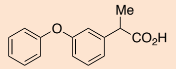	PEG-GGAATCG	2882.58	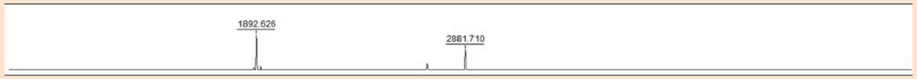
31	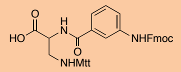	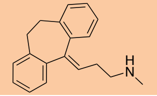	PEG-GAAGGGA	3068.73	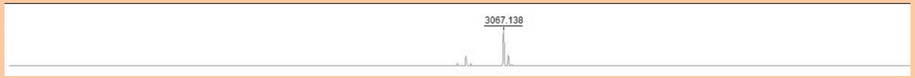
32	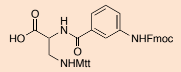	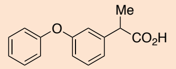	PEG-GAAGCCA	2851.58	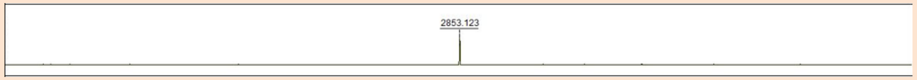
33	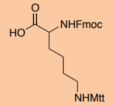	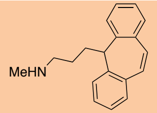	PEG-GAAGGTG	2982.60	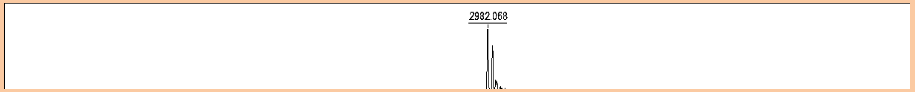
34	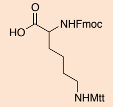	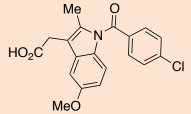	PEG-GAAGGCT	2920.98	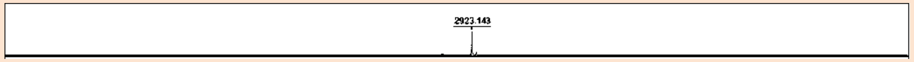
35	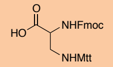	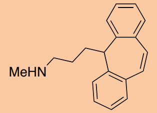	PEG-GGAGGAA	2949.61	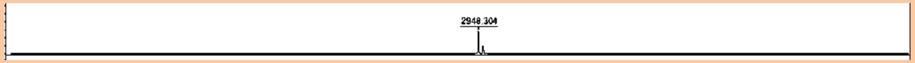
36	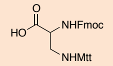	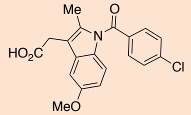	PEG-GGAGCTA	2878.98	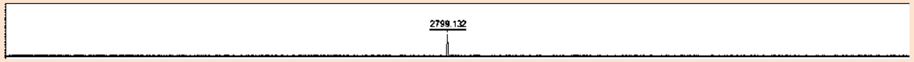
37	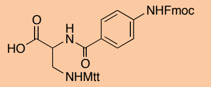	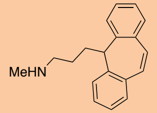	PEG-GGAGGTT	3050.71	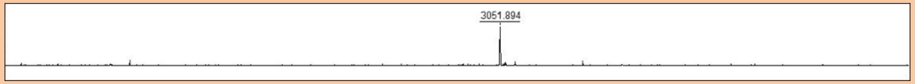
38	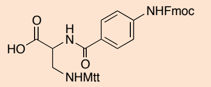	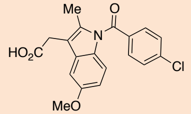	PEG-GTAGCGA	2998.10	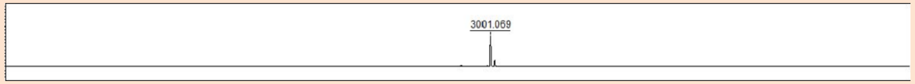

39	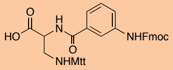	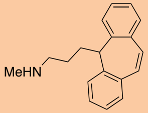	PEG-GTAGGAG	3059.72	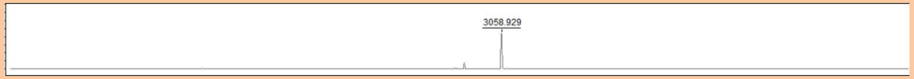
40	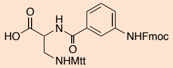	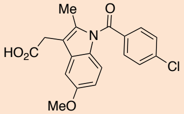	PEG-GTAGCTG	2989.09	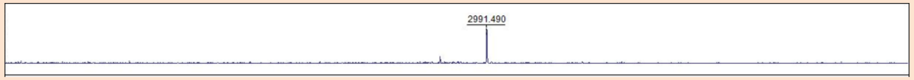
41	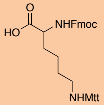	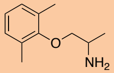	PEG-GTAGGGT	2889.47	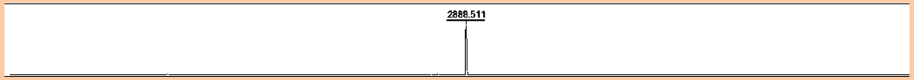
42	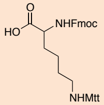	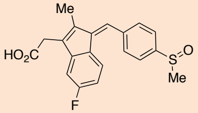	PEG-GTAGCCT	2870.58	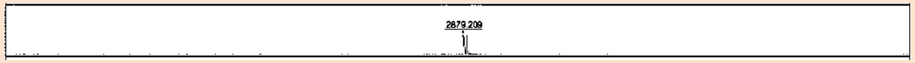
43	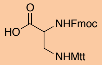	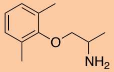	PEG-GTAGGTC	2807.47	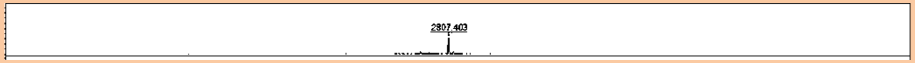
44	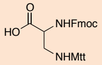	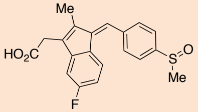	PEG-GCAGCAA	2846.60	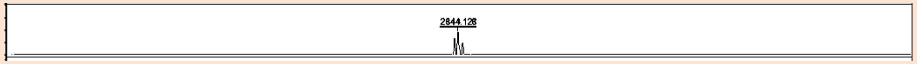
45	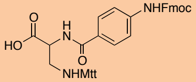	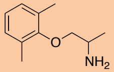	PEG-GCAGGAT	2935.60	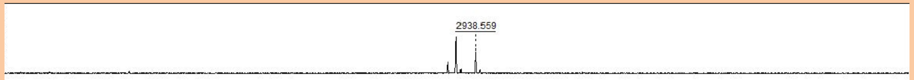
46	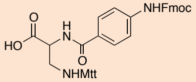	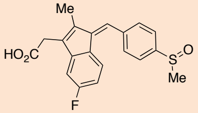	PEG-GCAGCTT	2947.70	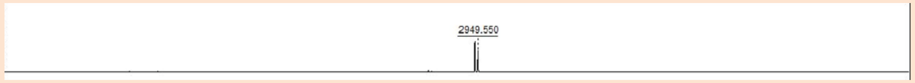
47	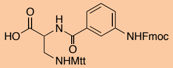	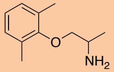	PEG-GAGGGTA	2975.60	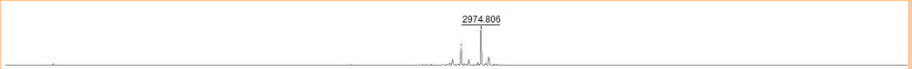
48	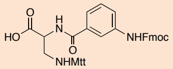	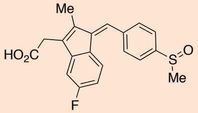	PEG-GTGGGAA	3036.72	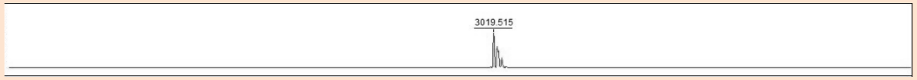

49	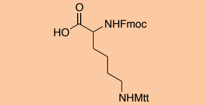	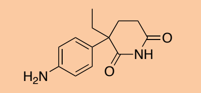	PEG-GTGGCTA	2902.53	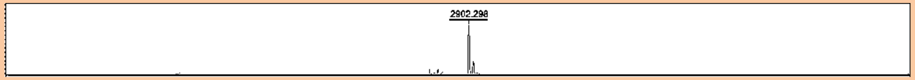
50	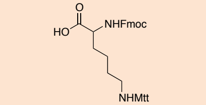	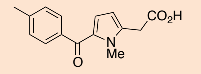	PEG-GTAGCCT	2841.36	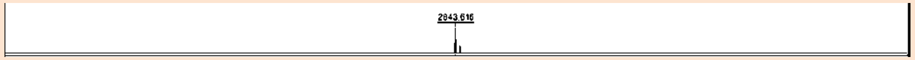
51	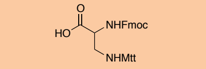	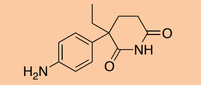	PEG-GATGGCA	2869.54	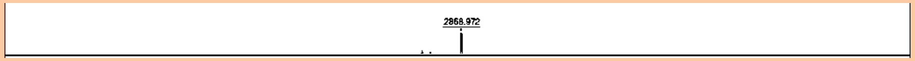
52	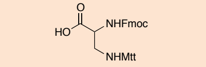	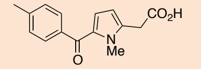	PEG-GGTGGTA	2808.37	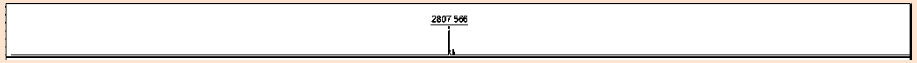
53	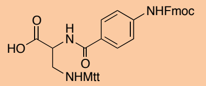	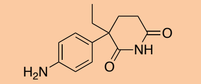	PEG-GTTGGGA	3019.65	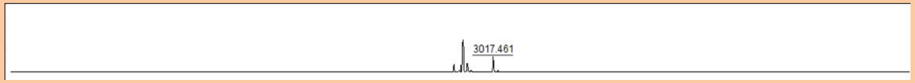
54	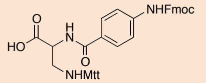	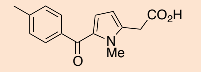	PEG-GTTGCCA	2847.48	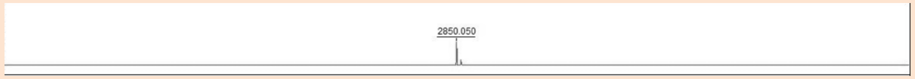
55	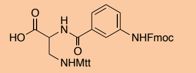	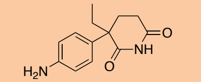	PEG-GTTGGTG	3010.64	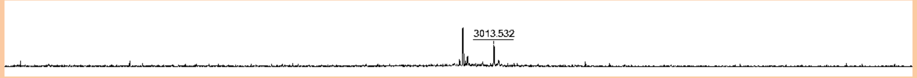
56	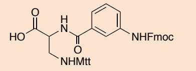	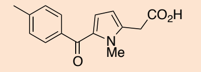	PEG-GTTGGCT	2878.47	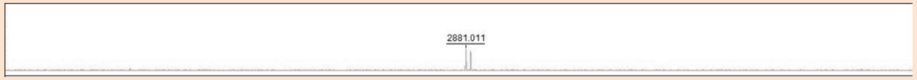
57	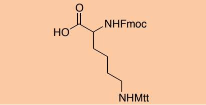	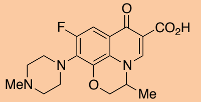	PEG-GCTGGAA	2924.56	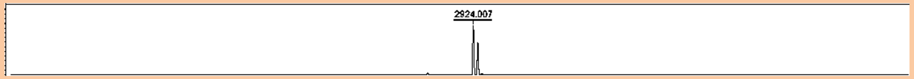
58	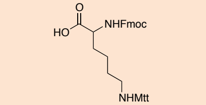	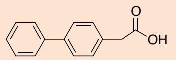	PEG-GCTGCTA	2726.41	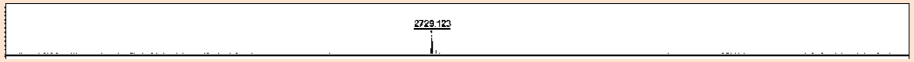
59	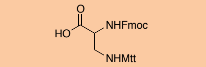	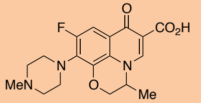	PEG-GCTGGTT	2864.53	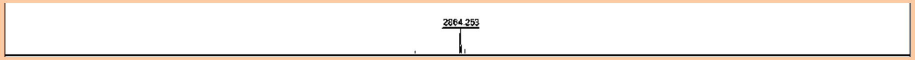

60	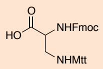	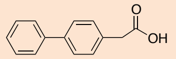	PEG-GTCGGTA	2724.42	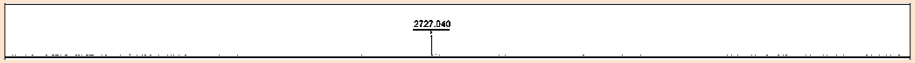
61	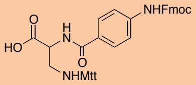	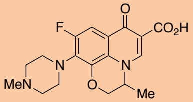	PEG-GAATGGG	3041.68	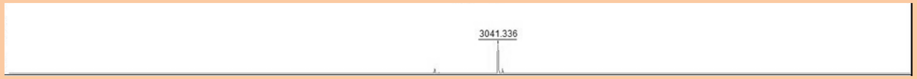
62	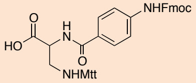	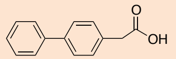	PEG-GAATCCG	2812.54	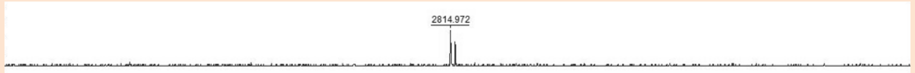
63	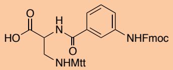	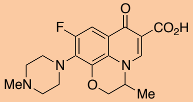	PEG-GGATCGA	3001.68	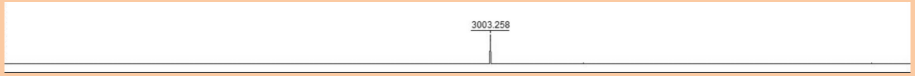
64	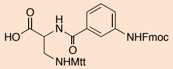	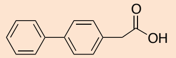	PEG-GGATGAG	2892.55	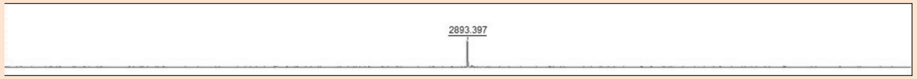
65	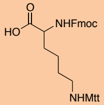	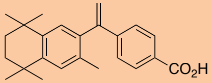	PEG-GGATCTG	2902.66	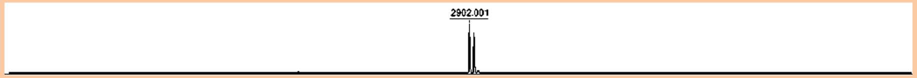
66	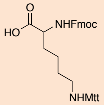	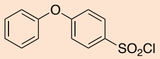	PEG-GGATGGT	2844.46	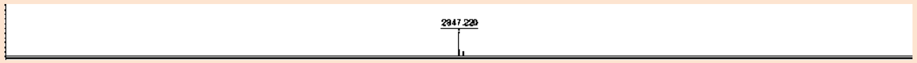
67	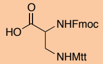	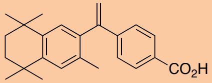	PEG-GGATCCT	2820.65	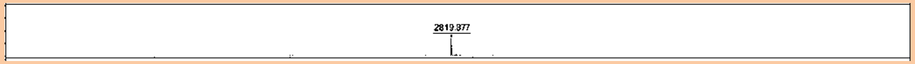
68	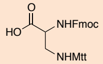	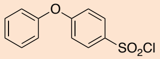	PEG-GGATGTC	2762.46	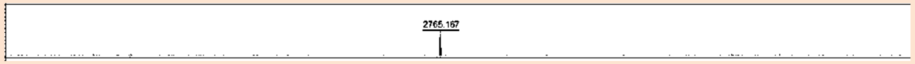
69	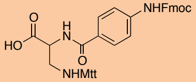	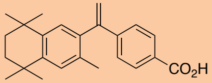	PEG-GTATCGG	2979.78	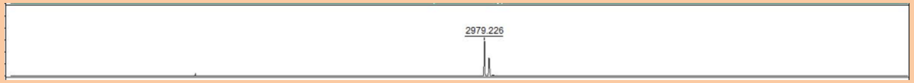
70	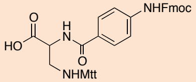	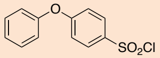	PEG-GTATGGC	2881.58	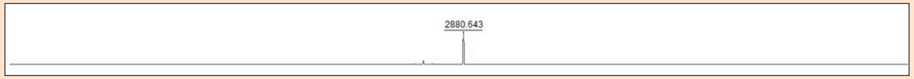

71	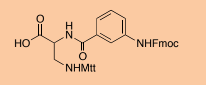	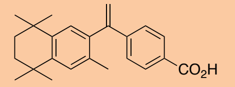	PEG-GTATCCC	2899.76	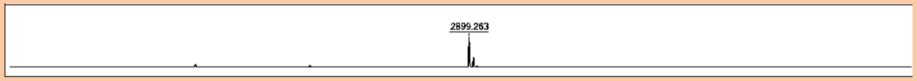
72	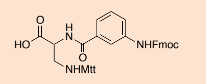	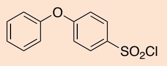	PEG-GCATCAG	2850.58	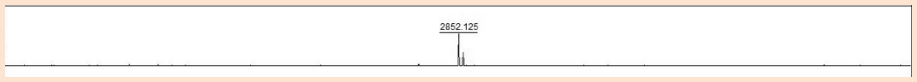
73	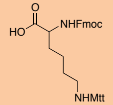	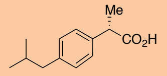	PEG-GCATCGT	2720.45	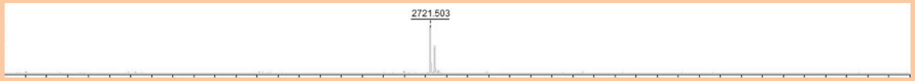
74	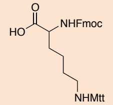	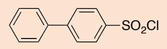	PEG-GCATGAC	2757.46	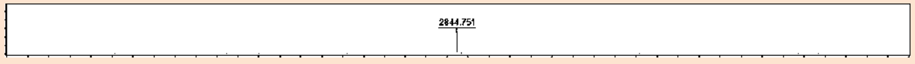
75	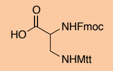	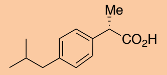	PEG-GCATCTC	2638.44	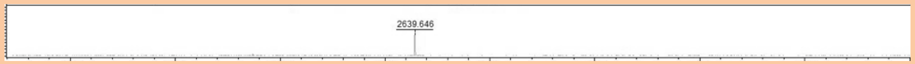
76	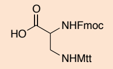	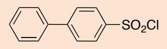	PEG-GAGTGGA	2795.47	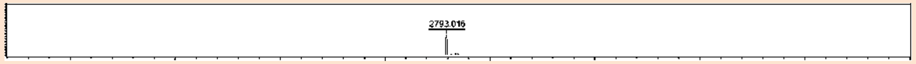
77	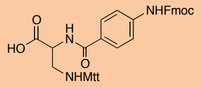	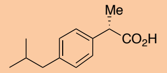	PEG-GAGTCCA	2806.58	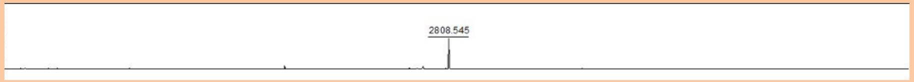
78	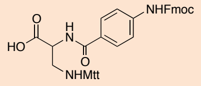	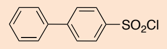	PEG-GAGTGTG	2905.58	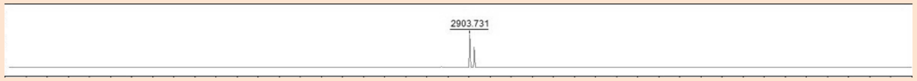
79	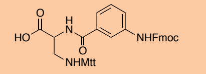	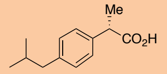	PEG-GAGTGCT	2837.58	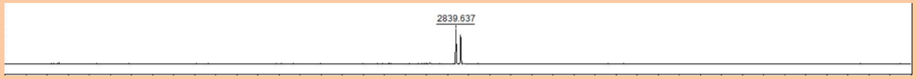
80	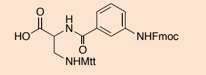	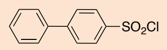	PEG-GGGTGAA	2914.59	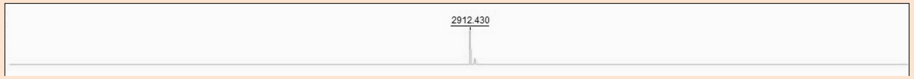
81	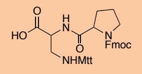	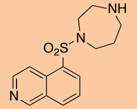	PEG-GGGTCTA	3016.67	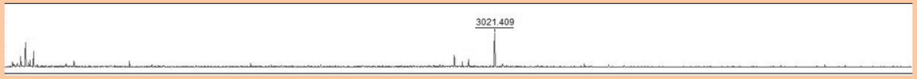

82	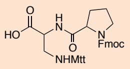	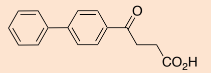	PEG-GGGTGTT	2894.50	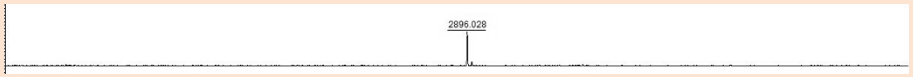
83	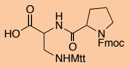	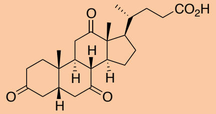	PEG-GTGTCGA	3011.75	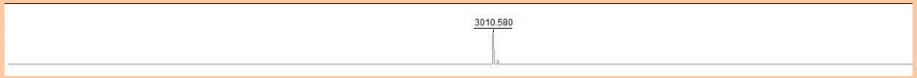
84	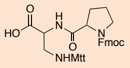	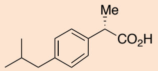	PEG-GCTTCGA	2775.50	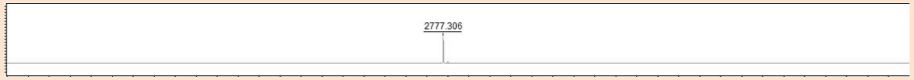
85	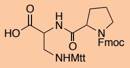	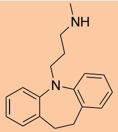	PEG-GTGTCTG	2982.72	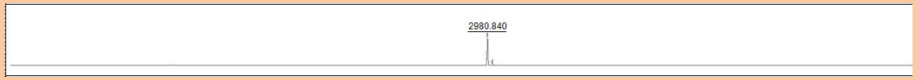
86	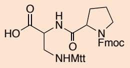	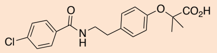	PEG-GTGTGGT	3002.04	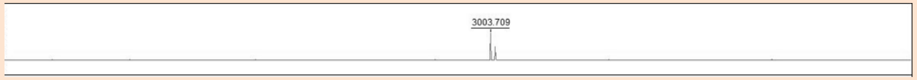
87	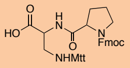	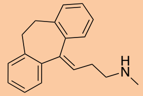	PEG-GTGTCCCT	2939.62	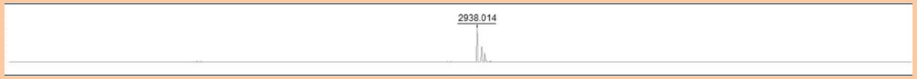
88	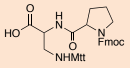	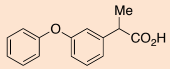	PEG-GTGTGTC	2842.48	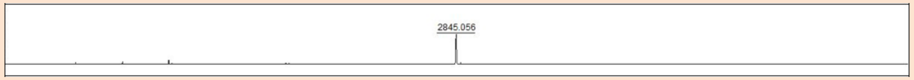
89	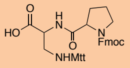	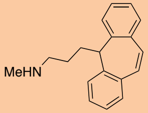	PEG-GCGTCAA	2957.64	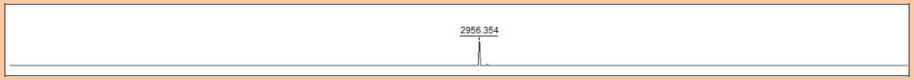
90	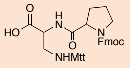	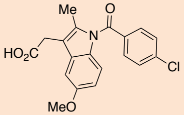	PEG-GCGTGAT	2967.02	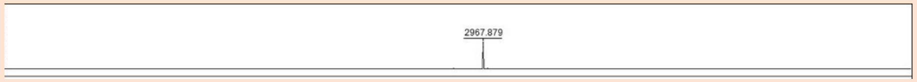
91	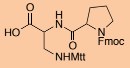	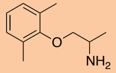	PEG-GCGTCTT	2855.50	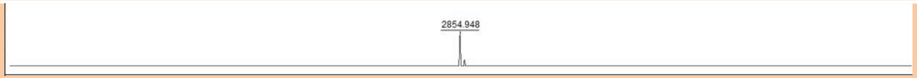
92	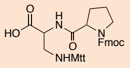	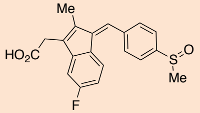	PEG-GATTGCG	2965.64	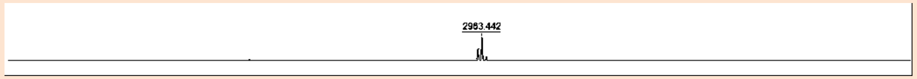

93	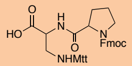	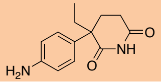	PEG-GGTTGGA	2997.58	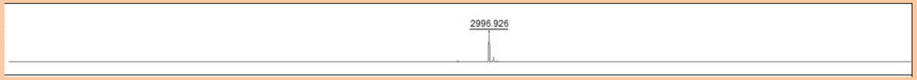
94	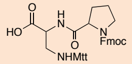	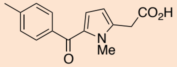	PEG-GGTTCCA	2825.41	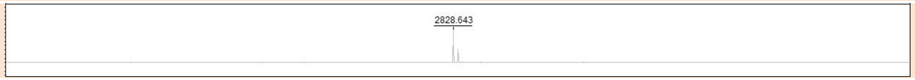
95	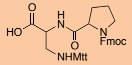	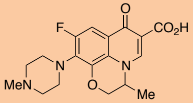	PEG-GGTTGTG	3001.59	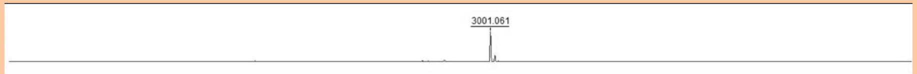
96	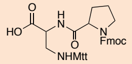	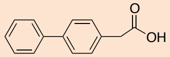	PEG-GGTTGCT	2812.45	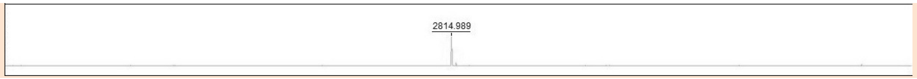
97	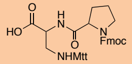	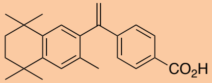	PEG-GTTTGGG	2988.70	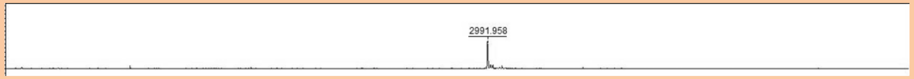
98	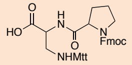	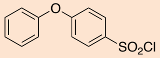	PEG-GTTTCCG	2810.49	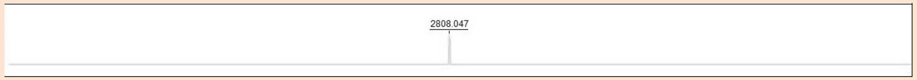
99	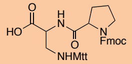	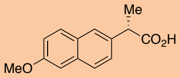	PEG-GTGTGAG	2879.49	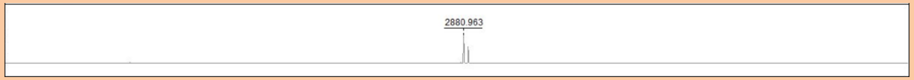
100	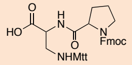	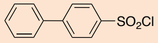	PEG-GCTTGAG	2843.51	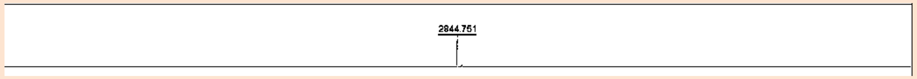

Introduction of Fragments B

The 100 pools from the previous steps were mixed and split into 100 new pools each containing a mixture of 100 different compounds.

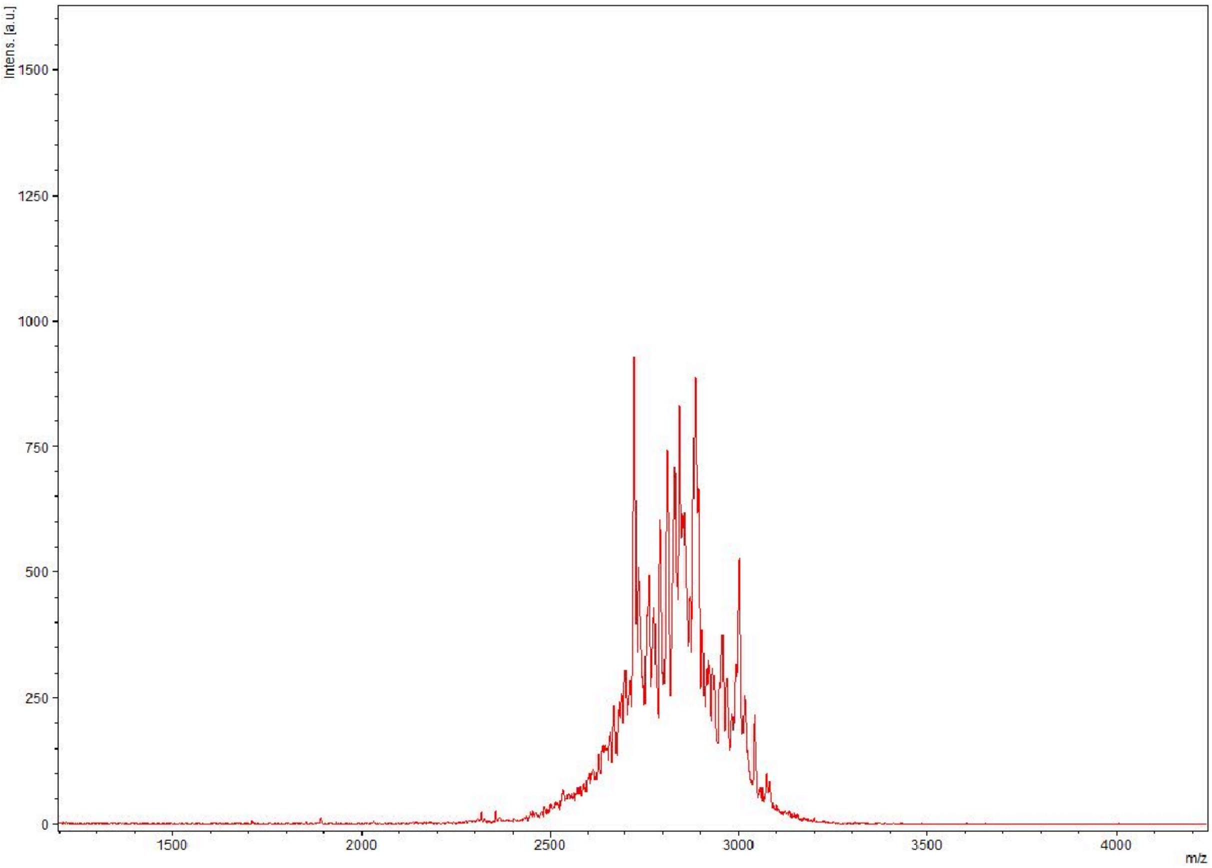

MALDI analysis of the mixture of 100 compounds after mix and split.

Each pool was then encoded with the corresponding 7mer PNA codon PNA using the s. Finally, Mtt was deprotected (***procedure 9***) and 100 different fragments (one in each pool) were coupled through the same procedures as the first fragments (***procedure 10***). MALDI analysis of each pool confirmed the expected molecular weight range. MALDI spectra (2,5-Dihydroxybenzoic acid or a-Cyano-4-hydroxycinnamic acid matrix and desorbed with laser between 15-55%) were recorded from 2000 to 7000 m/z.

Codon 2	Fragment B	PNA sequence	Mass Range	MALDI
1	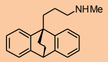	CTTCTGG-Lys	4730.35-5174.74	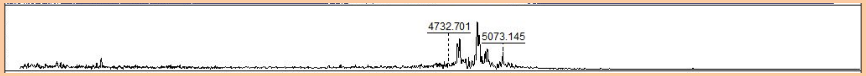
2	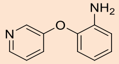	CTTGGTG-Lys	4678.95-5123.34	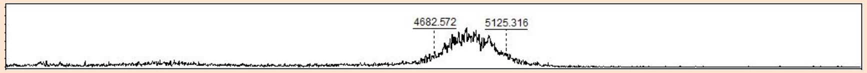
3	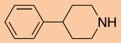	CTTCCTG-Lys	4588.21-5032.60	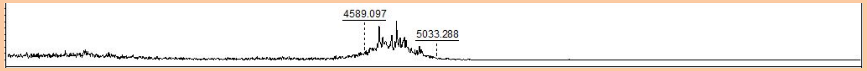
4	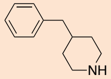	CTTGTCG-Lys	4615.18-5059.57	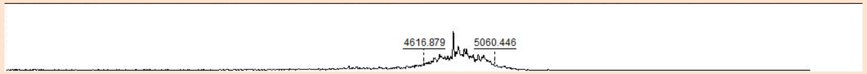
5	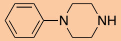	ACTGCAG-Lys	4647.23-5091.62	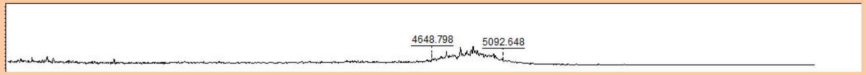
6	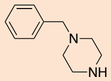	GCTGTAG-Lys	4501.96-4946.35	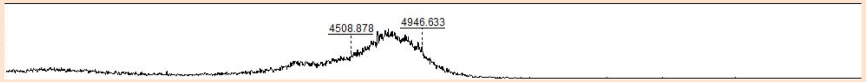
7	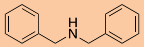	TCTGGAG-Lys	4699.24-5143.63	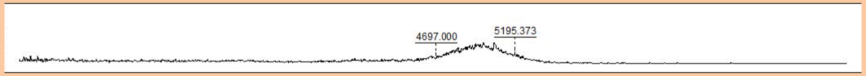
8	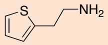	TCTCCAG-Lys	4579.15-5023.54	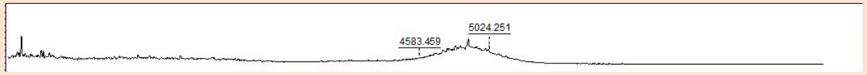
9	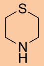	TCTGTGG-Lys	4596.15-5040.54	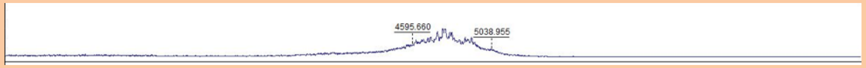
[illegible]	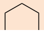	[illegible]	[illegible]	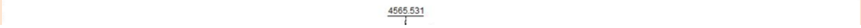

12	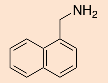	CCTCTAG-Lys	4578.95-5023.34	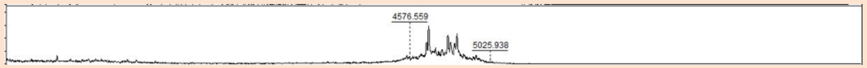
13	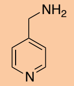	CCTGTTG-Lys	4561.09-5005.48	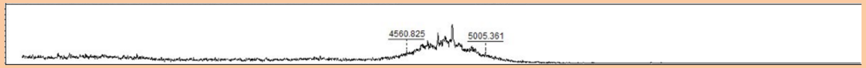
14	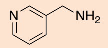	AACCGAG-Lys	4588.12-5032.51	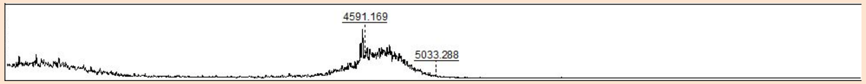
15	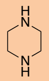	AACGAGG-Lys	4706.25-5150.64	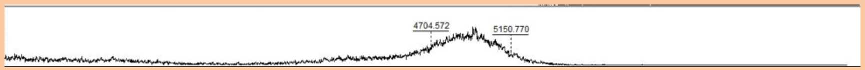
16	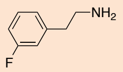	AACCTGG-Lys	4610.17-5054.56	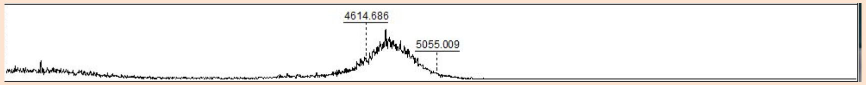
17	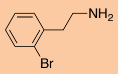	AACGGTG-Lys	4711.07-5155.46	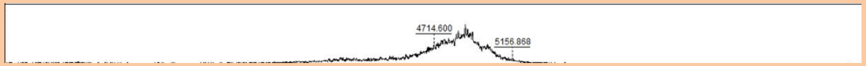
18	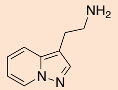	AACCCTG-Lys	4591.96-5036.35	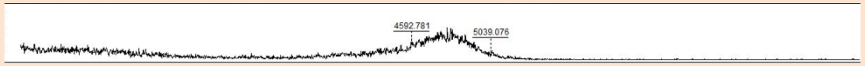
19	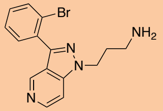	AACGTCG-Lys	4801.97-5246.36	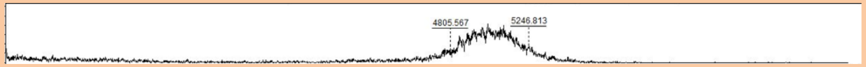
20	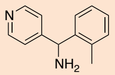	GACCAAG-Lys	4677.98-5122.37	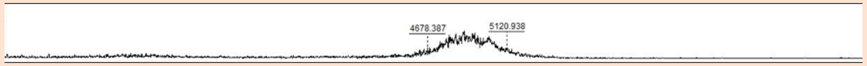
21	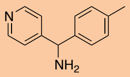	GACGATG-Lys	4708.97-5153.36	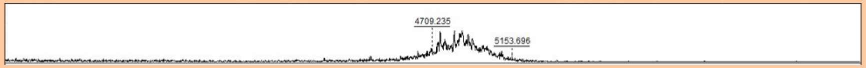
22	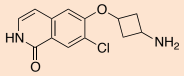	GACCTTG-Lys	4725.96-5170.35	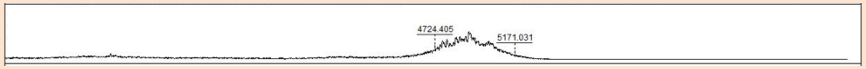
23	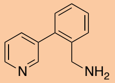	TACCAGG-Lys	4654.97-5099.36	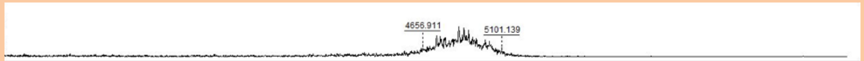

24	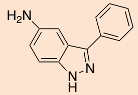	TACCGTG-Lys	4670.961-5115.351	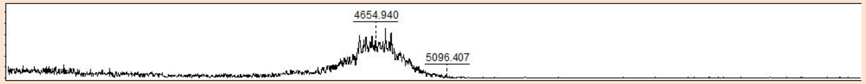
25	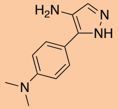	TACGACG-Lys	4672.973-5117.363	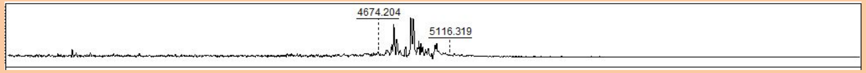
26	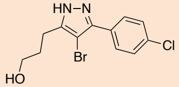	TACCTCG-Lys	4736.955-5181.345	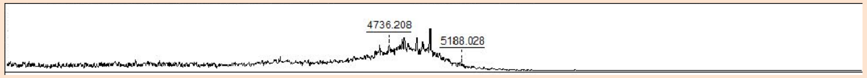
27	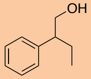	CACCATG-Lys	4581.186-5025.576	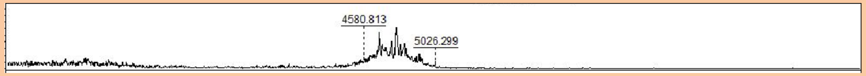
28	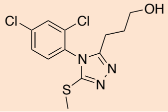	AGCGAAG-Lys	4837.99-5282.38	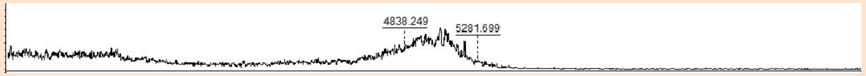
29	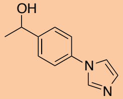	AGCCTAG-Lys	4658.973-5103.363	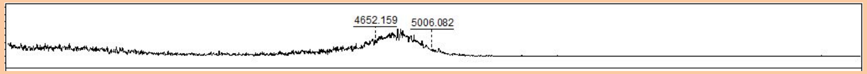
30	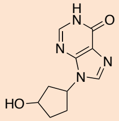	AGCGTTG-Lys	4721.967-5166.357	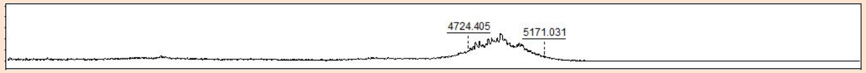
31	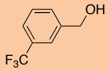	TGCCAAG-Lys	4647.113-5091.503	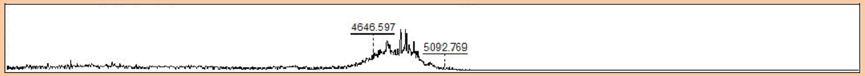
32	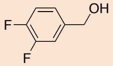	TGCGATG-Lys	4646.087-5090.477	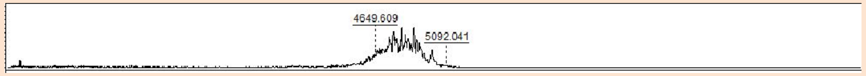
33	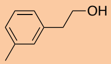	TGCCTTG-Lys	4589.15-5033.54	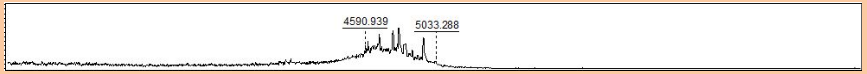
34	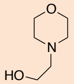	ATCGGAG-Lys	4642.179-5086.569	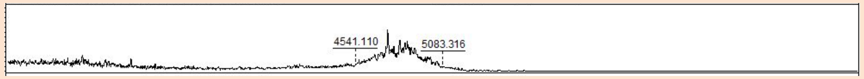

35	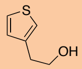	ATCCCAG-Lys	4559.166-5003.556	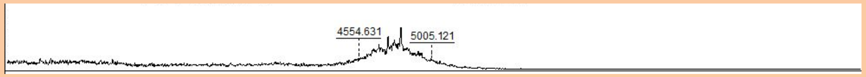
36	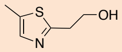	ATCGTGG-Lys	4645.167-5089.557	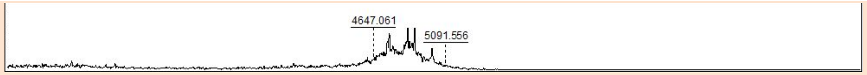
37	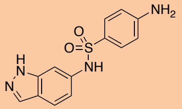	ATCGCTG-Lys	4749.961-5194.351	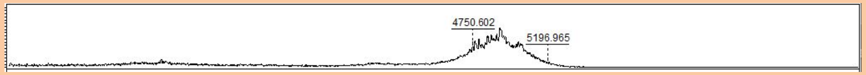
38	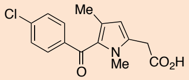	GTCGAAG-Lys	4757.579-5201.969	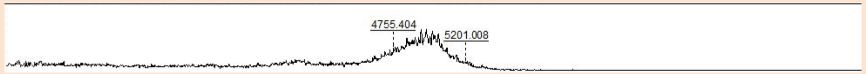
39	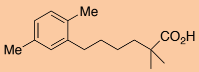	GTCCTAG-Lys	4668.261-5112.651	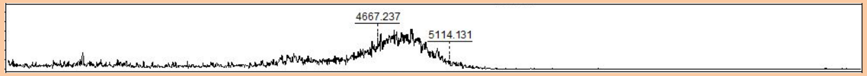
40	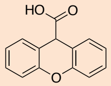	GTCGTTG-Lys	4675.186-5119.576	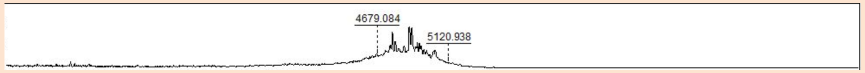
41	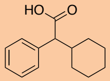	TTCCGAG-Lys	4636.251-5080.641	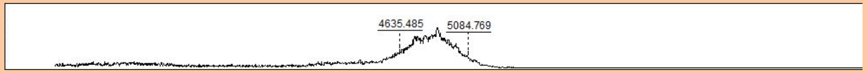
42	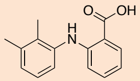	TTCGAGG Lys	4699.257-5143.647	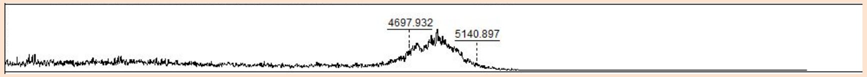
43	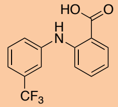	TTCCTGG-Lys	4690.18-5164.57	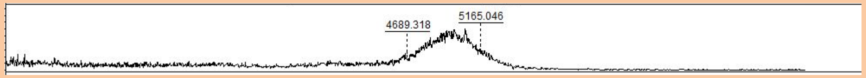
44	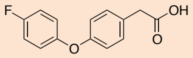	TTCGGTG-Lys	4695.186-5139.576	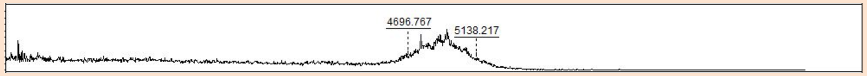
45	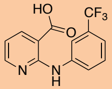	TTCCCTG-Lys	4651.163-5095.553	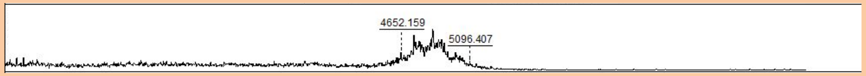

46	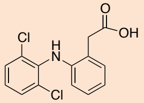	TTCGTCG-Lys	4727.08-5171.47	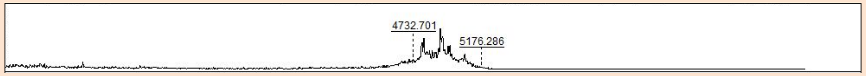
47	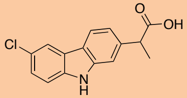	CTCCAAG-Lys	4660.676-5105.066	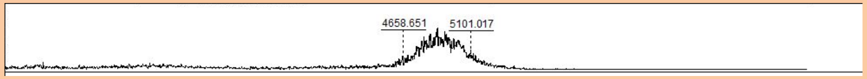
48	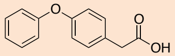	CTCGATG-Lys	4646.201-5090.591	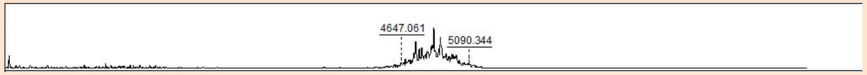
49	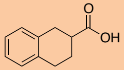	CTCCTTG-Lys	4545.153-4989.543	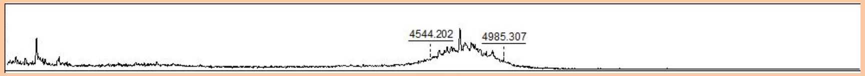
50	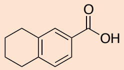	ACCGTAG-Lys	4603.183-5047.573	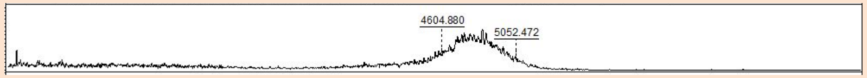
51	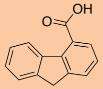	TCCGAAG-Lys	4637.203-5081.593	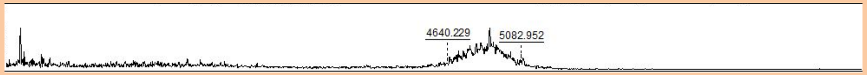
52	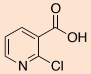	TCCCTAG-Lys	4535.515-4979.905	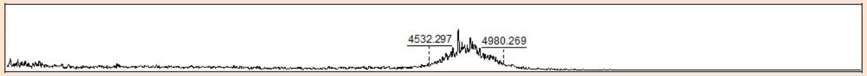
53	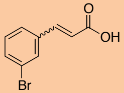	TCCGTTG-Lys	4635.99-5080.38	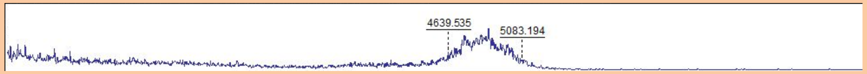
54	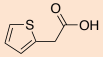	GCGTAAG-Lys	4609.159-5053.549	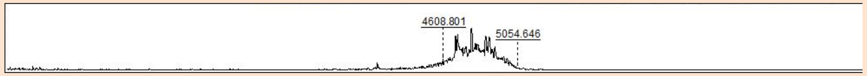
55	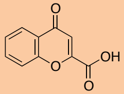	GCGATAG-Lys	4657.129-5101.519	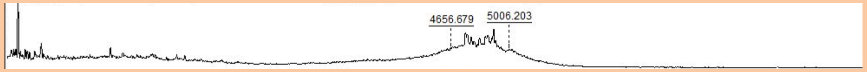
56	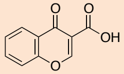	GCGAATG-Lys	4657.129-5101.519	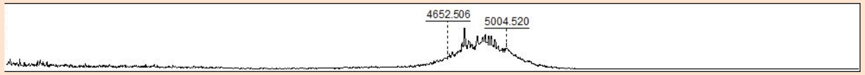
57	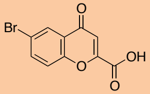	GCGTATG-Lys	4727.017-5171.407	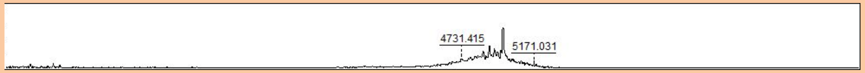

58	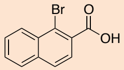	GCGATTG-Lys	4709.047-5153.437	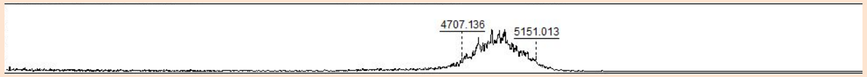
59	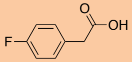	GCGTTTG-Lys	4603.096-5047.486	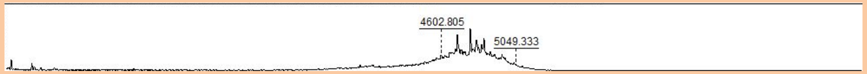
60	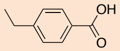	CGCATAG-Lys	4577.143-5021.533	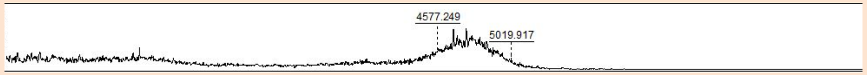
61	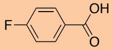	CGCTTAG-Lys	4558.081-5002.471	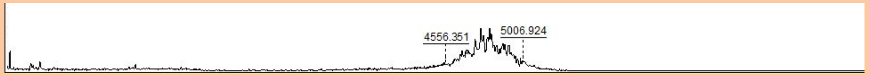
62	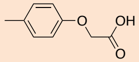	CGCATTG-Lys	4584.161-5028.551	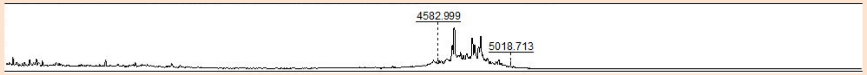
63	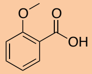	CGCTTTG-Lys	4561.1-5005.49	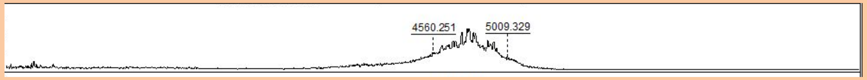
64	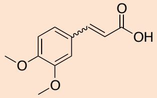	GGGAAAG-Lys	4724.207-5168.597	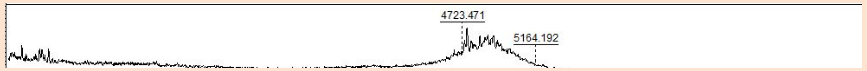
65	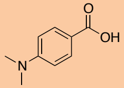	GGGTTAG-Lys	4663.164-5107.554	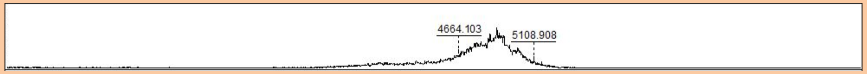
66	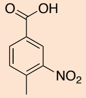	GGGTATG-Lys	4679.124-5123.514	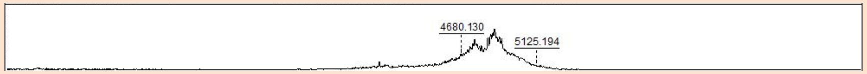
67	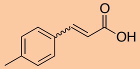	GGGATTG-Lys	4660.164-5104.554	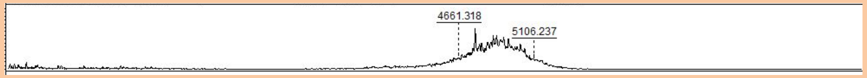
68	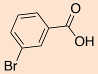	GGGTTTG-Lys	4689.982-5134.372	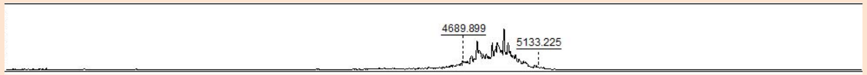
69	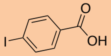	CCGAAAG-Lys	4684.004-5133.394	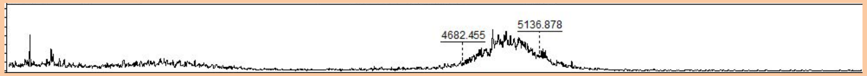

70	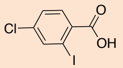	CCGTTAG-Lys	4700.421-5144.811	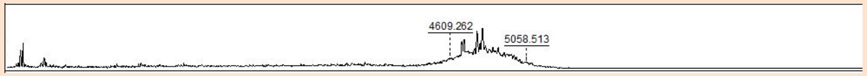
71	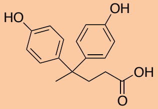	CCGTATG-Lys	4704.281-5148.671	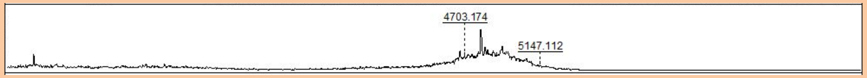
72	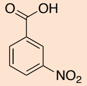	CCGATTG-Lys	4585.081-5029.471	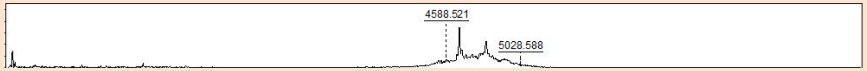
73	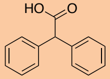	CCGTTTG-Lys	4621.2-5065.59	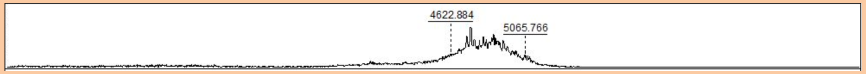
74	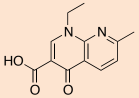	TTGCCAG-Lys	4649.961-5094.351	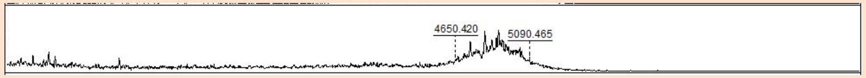
75	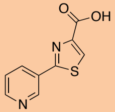	TTGAGGG-Lys	4703.974-5148.364	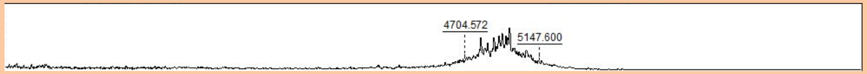
76	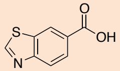	TTGGCTG-Lys	4627.956-5072.346	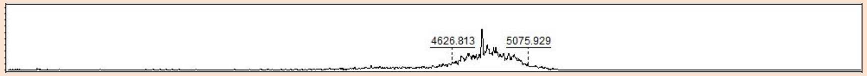
77	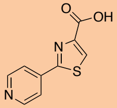	TTGCACG-Lys	4623.961-5068.351	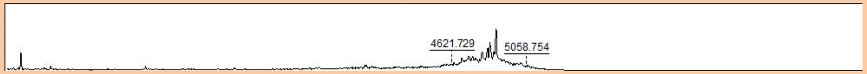
78	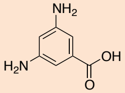	TTGTGCG-Lys	4448.956-4893.346	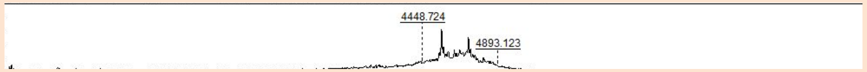
79	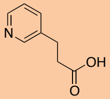	TTGACCG-Lys	4568.961-5013.351	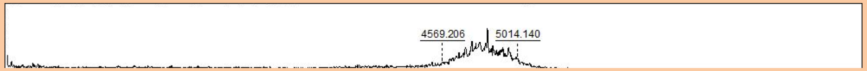
80	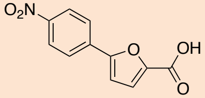	TTACCCG-Lys	4610.955-5055.345	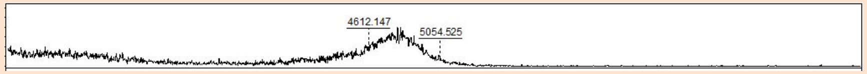

**81**	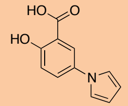	TTCGCAG-Lys	4620.961-5065.351	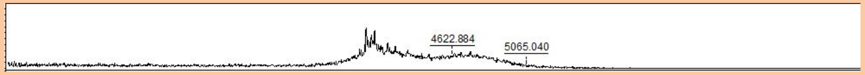
**82**	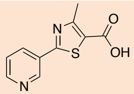	TTCTGGG-Lys	4668.956-5113.346	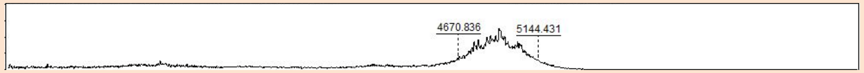
**83**	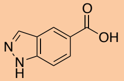	TTCGTGG-Lys	4610.956-5055.346	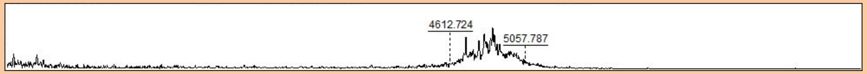
**84**	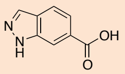	TTCACGG-Lys	4579.961-5024.351	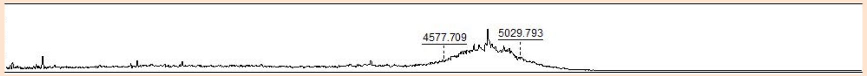
**85**	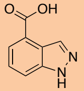	TTCAGCG-Lys	4579.961-5024.351	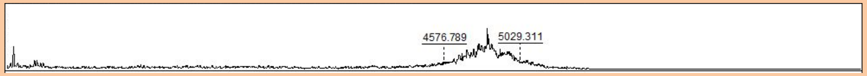
**86**	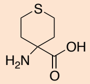	TTCTCCG-Lys	4368.943-4813.333	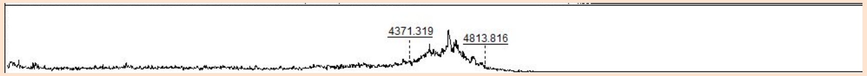
**87**	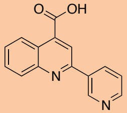	AAAGGGG-Lys	4765.997-5210.387	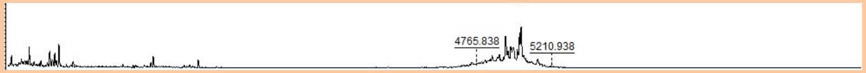
**88**	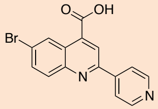	AAACCGG-Lys	4764.984-5209.374	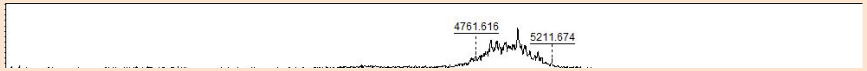
**89**	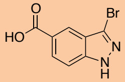	AAAGCCG-Lys	4676.984-5121.374	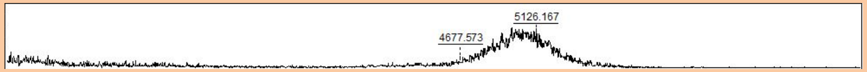
**90**	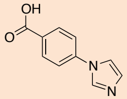	GGCTATG-Lys	4645.967-5090.357	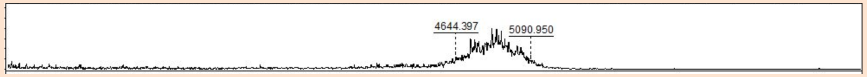
**91**	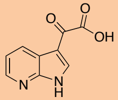	AATGCCG-Lys	4616.973-5061.363	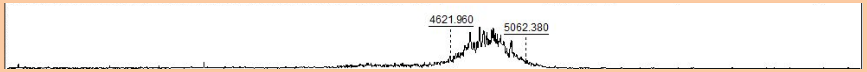

92	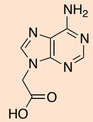	TTTGCCG-Lys	4601.95-5046.34	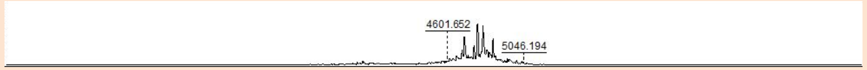
93	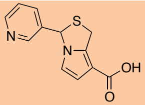	AATCTAG-Lys	4671.977-5116.367	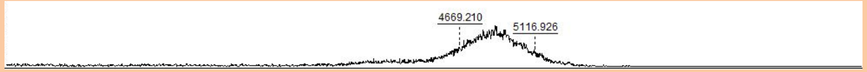
94	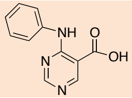	TAACGCG-Lys	4641.973-5086.363	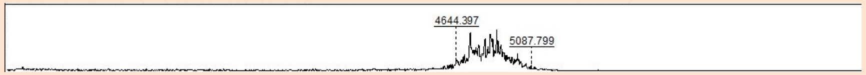
95	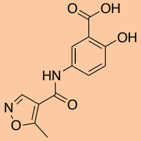	AGAGGTG-Lys	4768.985-5213.375	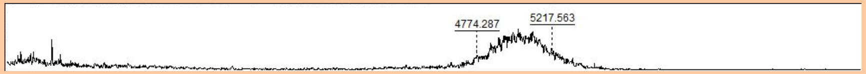
96	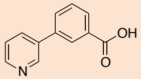	AGAGACG-Lys	4674.99-5119.38	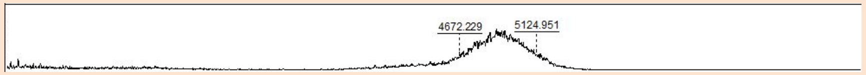
97	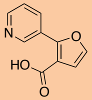	AGACCTG-Lys	4615.973-5060.363	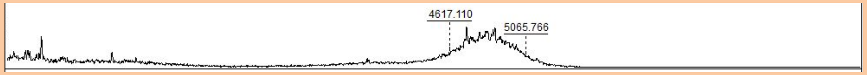
98	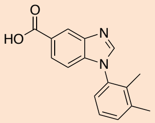	AGAAGCG-Lys	4741.99-5186.38	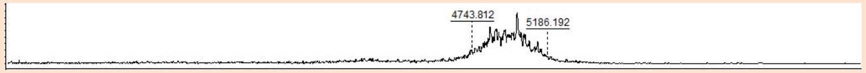
99	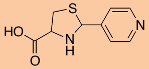	AGACTGG-Lys	4676.979-5121.369	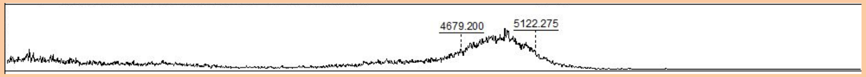
100	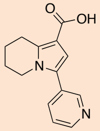	AGATGAG-Lys	4732.99-5177.38	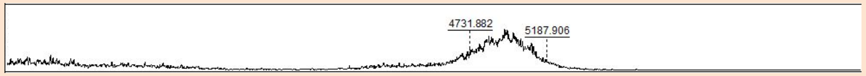

The 100 pools were mixed and TFA cleavage (***procedure 11***) afforded the Hsp70 focused library of 10,000 members.

5. Screening of the focused library

Array with the complementary DNA sequences (4 copies randomly distributed on the array) to the PNA sequences were ordered from Agilent (Agilent Earray design 041896). Recombinant Hsp70-GST protein (Signal Chem) was diluted to 100nM in buffer [200 mM HEPES pH 7.4, 0.5 M KCl, 50 mM $MgCl_2$, 200 mM Na_2MO_4, 0.1% Tergitol, 2 mM DTT]. In parallel, solution containing an additional 100 nM Hsp40 (SignalChem: H32-54G) or 1mM ATP were prepared. Then the solutions were added to the microarray and mixed by rotation for two hours at room temperature. After, the hybridization chamber was dismounted and the array was washed twice with PBS-T and once with mQ water just before being dried by centrifugation (5 min at 1,000 g). The slide was placed again in the hybridization chamber and incubated with anti-GST DyLight 649 coupled antibody (Rockland Immunogenics, diluted 1/5000 in PBST, 0.5%BSA) for 20 min. The microarray were washed and dried as before. The slide was scanned at 635 nm on a Genepix Personnal Scanner.

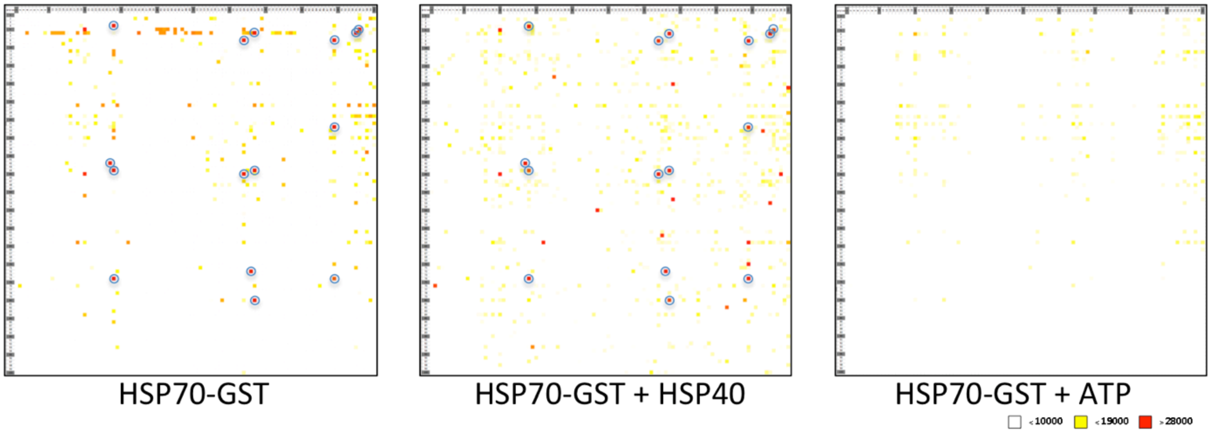

6. Characterization of selected binders

LC (Total Scan 190-800nm)

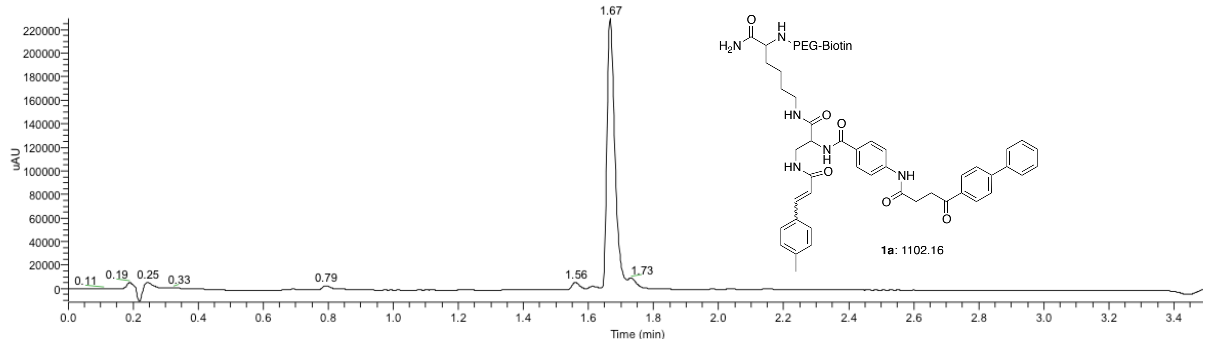

ESI (150-2000)

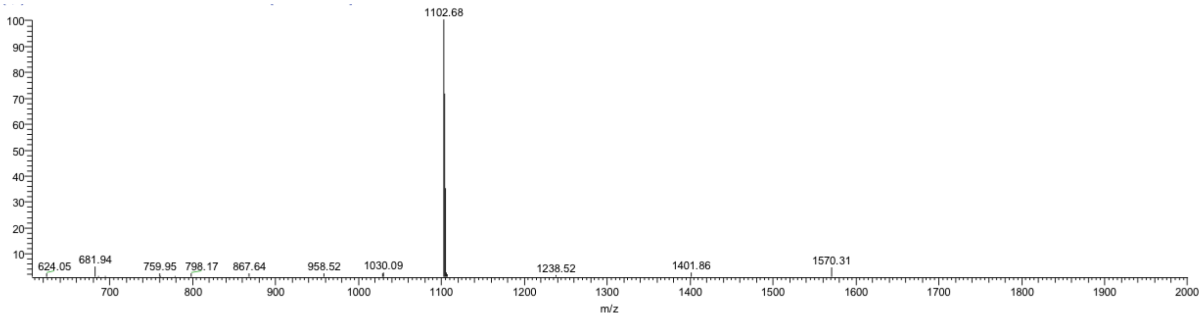

NMR date of compound 1a

^{1}H NMR (DMSO-d6, 500 MHz, 23 °C) δ = 10.23 (s, 1H); 8.35(d, *J* = 1.6 Hz, 1H); 8.19 (t, *J* = 4.9 Hz, 1H); 8.02 (d, *J* = 8.5 Hz, 2H); 7.78 (d, *J* = 8.5 Hz, 2H); 7.76 (d, *J* = 8.7 Hz, 2H); 7.70 (d, *J* = 8.4 Hz, 2H); 7.62 (d, *J* = 8.8 Hz, 2H); 7.45 (t, *J* = 7.9 Hz, 2H); 7.38-7.32 (m, 5H), 7.34 (d, *J* = 15.3 Hz, 1H); 7.14 (d, *J* = 8.5 Hz, 2H); 6.54 (d, *J* = 15.4 Hz, 1H); 4.41-4.37 (m, 1H); 3.57-3.47 (m, 2H); 3.32 (t, *J* = 6.5 Hz, 2H); 2.71 (t, *J* = 6.5 Hz, 2H); 2.23 (s, 3H) ppm. ^{13}C NMR (DMSO-d6, 100 MHz, 23 °C) δ = 198.8, 172.6, 171.3, 167.1, 166.2, 145.0, 142.5, 139.7, 139.5, 139.4, 135.8, 132.5, 129.9 (x2), 129.6 (x2), 129.1 (x2), 128.8 (x2), 128.7 (x2), 128.6, 128.0 (x2), 127.5 (x2), 127.4 (x2), 121.3, 118.4 (x2), 54.9, 41.2, 33.5, 30.8, 21.4 ppm.

LC (Total Scan 190-800nm)

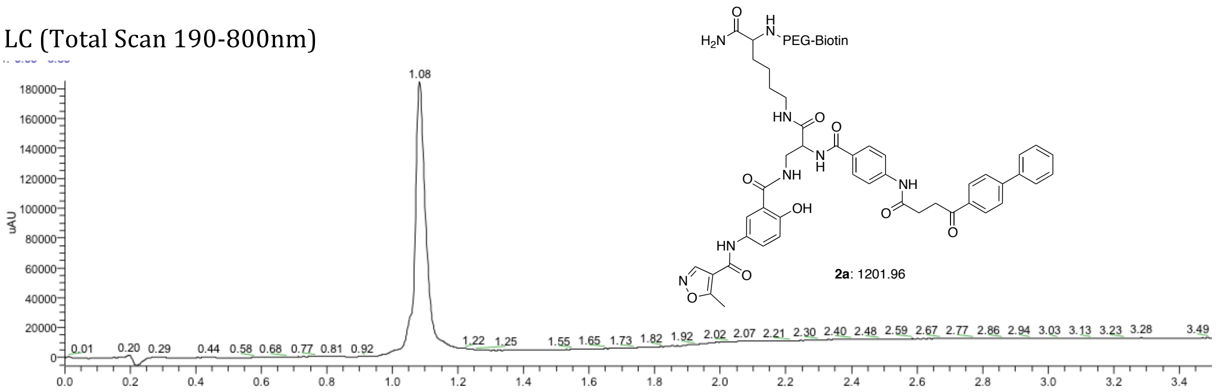

ESI (150-2000)

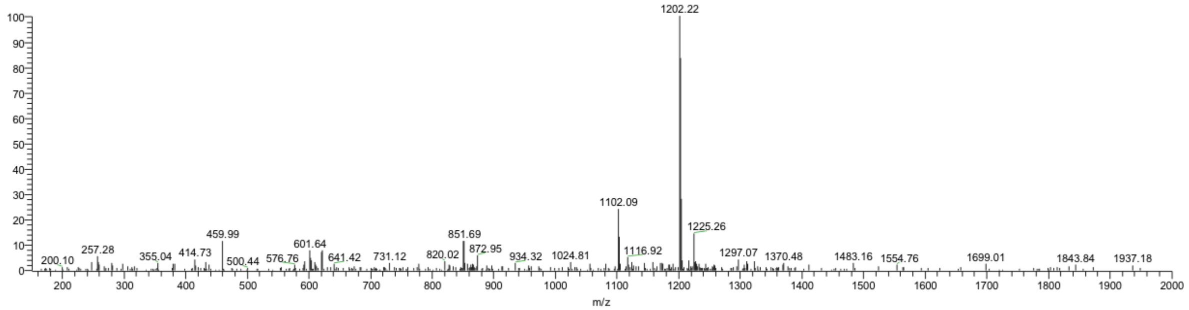

NMR date of compound 2a

^{1}H NMR (DMSO-d6, 500 MHz, 23 °C) δ = 11.6 (s, 1H); 10.3 (s, 1H); 9.98 (s, 1H); 8.81(t, *J* = 5.9 Hz, 1H); 8.44 (d, *J* = 7.8 Hz, 2H); 8.08 (d, *J* = 8.5 Hz, 2H); 8.05 (d, *J* = 2.8 Hz, 1H); 7.83 (t, *J* = 8.6 Hz, 2H); 7.75 (d, *J* = 7.3 Hz, 2H); 7.66 (d, *J* = 8.8 Hz, 2H); 7.63 (dd, *J* = 8.8, 2.8 Hz, 1H); 7.50 (t, *J* = 7.3 Hz, 2H); 7.48 (bs, 2H), 7.43 (d, *J* = 6.7 Hz, 1H); 7.16 (s 1H), 6.89 (d, *J* = 8.5 Hz, 1H); 4.59-4.54 (m, 1H), 3.48-3.77 (m, 1H), 3.65-3.57 (m, 1H), 3.37 (t, *J* = 6.6 Hz, 2H); 2.77 (t, *J* = 6.6 Hz, 2H); 2.65 (s, 3H) ppm. ^{13}C NMR (DMSO-d6, 100 MHz, 23 °C) δ = 198.9, 173.2, 172.4, 171.3, 167.4, 166,4, 159.3, 154.8, 149.3 (x2), 144.9, 142.5, 139.5, 135.7, 130.3, 129.6 (x2), 129.1 (x2), 128.9, 128.8 (x2), 128.7, 127.5 (x2), 127.4 (x2), 127.0m 122.5, 118.5, 117.4, 112.2, 70.4, 54.2, 41.4, 33.5, 30.9, 12.1 ppm.

LC (Total Scan 190-800nm)

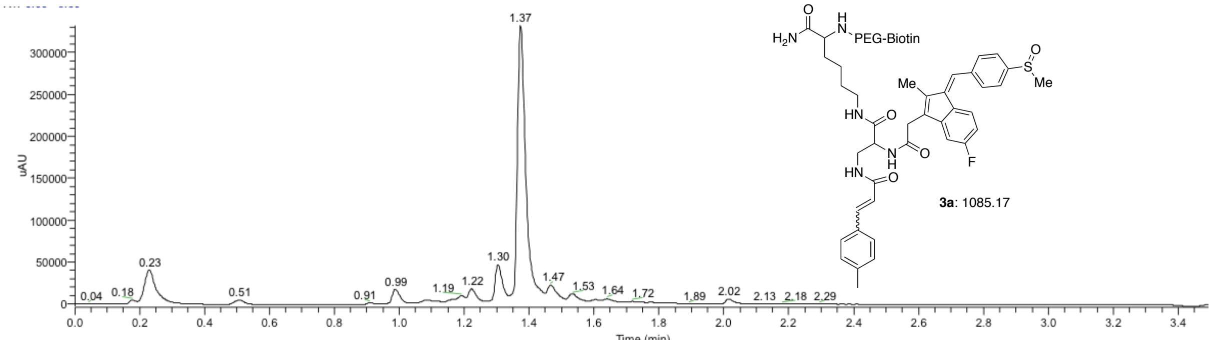

ESI (150-2000)

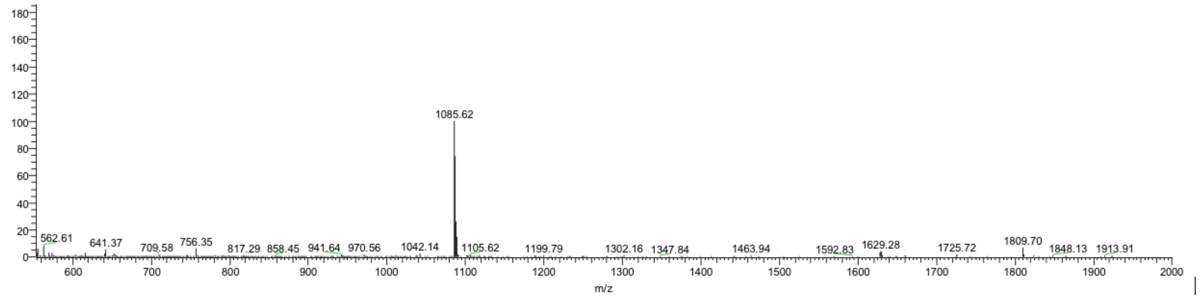

LC (Total Scan 190-800nm)

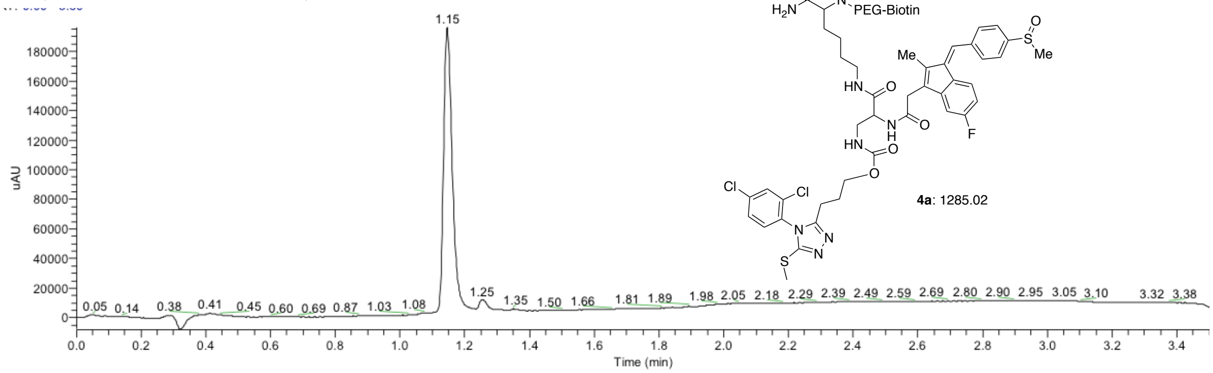

ESI (150-2000)

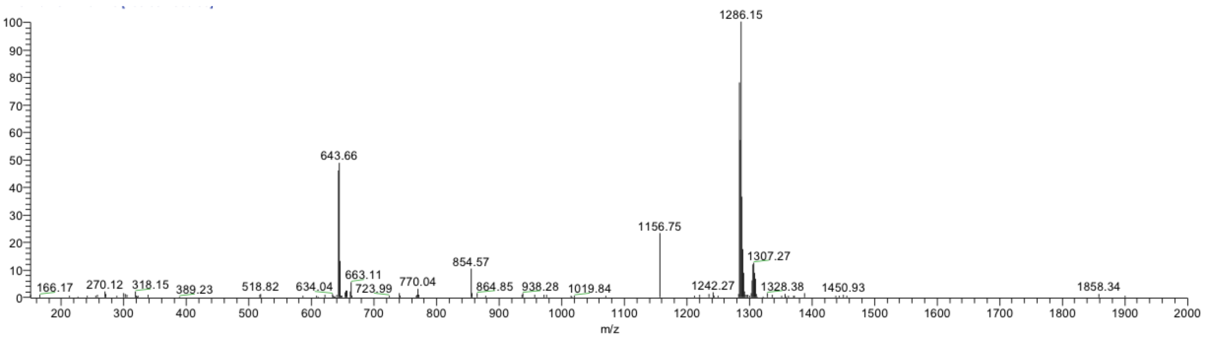

LC (Total Scan 190-800nm)

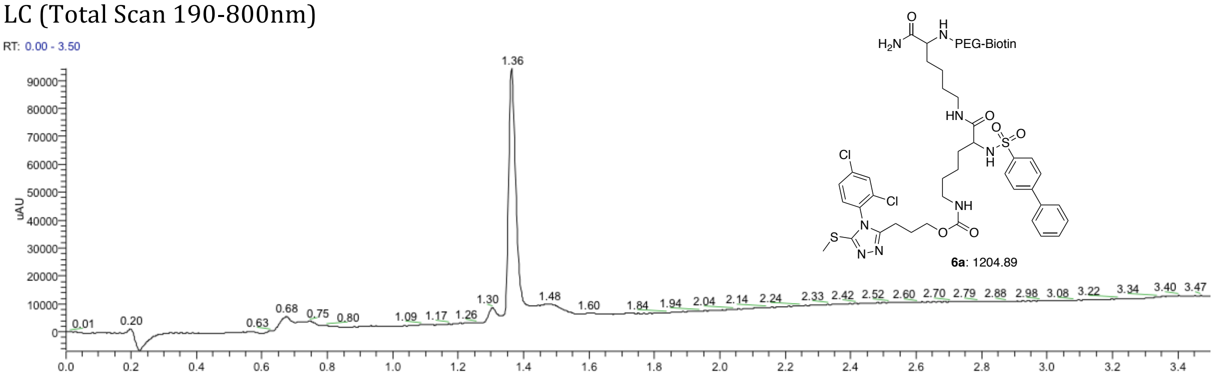

6a: 1204.89

ESI (150-2000)

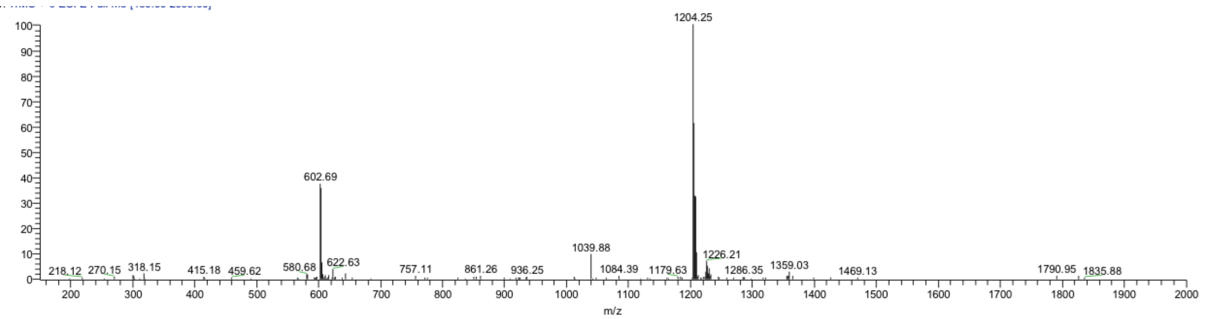

LC (Total Scan 190-800nm)

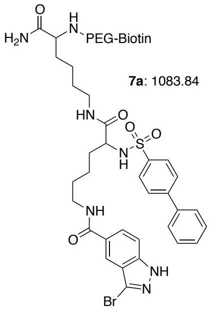

7a: 1083.84

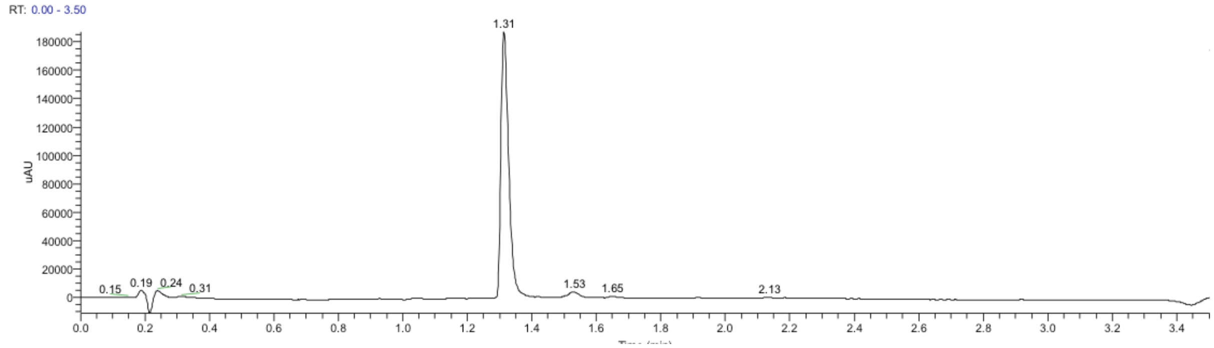

ESI (150-2000)

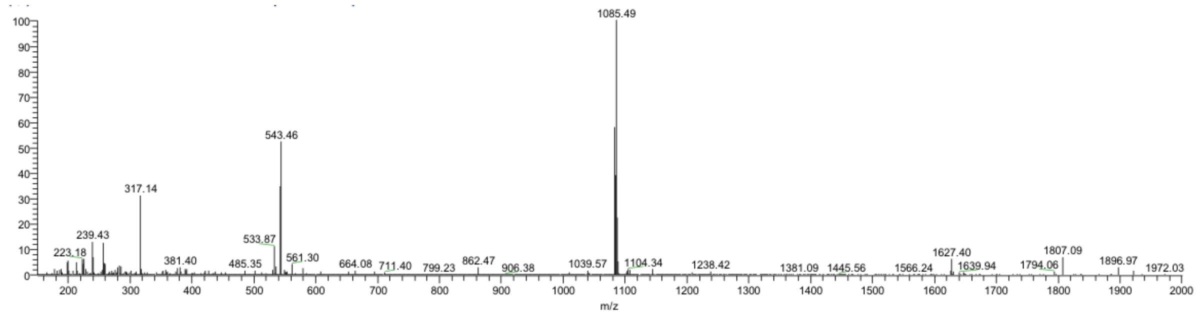

LC (Total Scan 190-800nm)

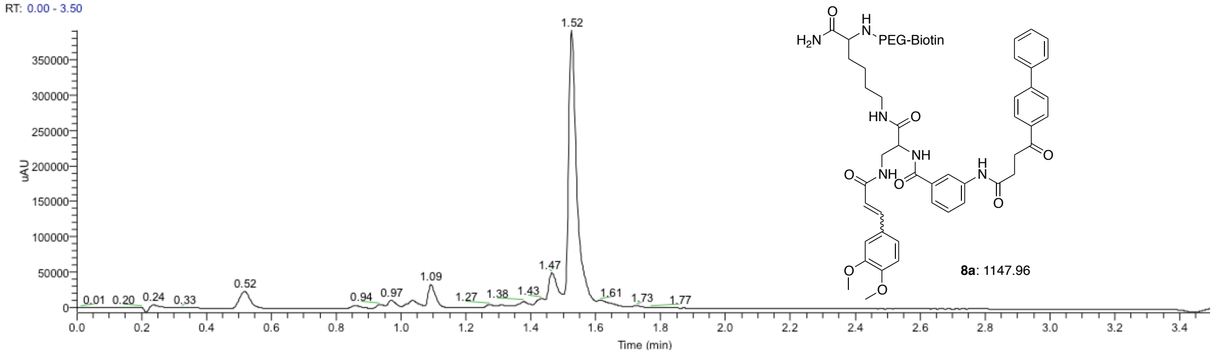

ESI (150-2000)

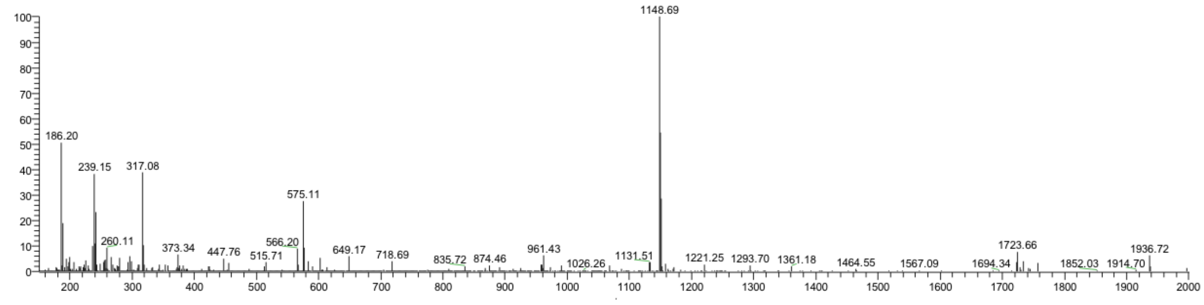

LC (Total Scan 190-800nm)

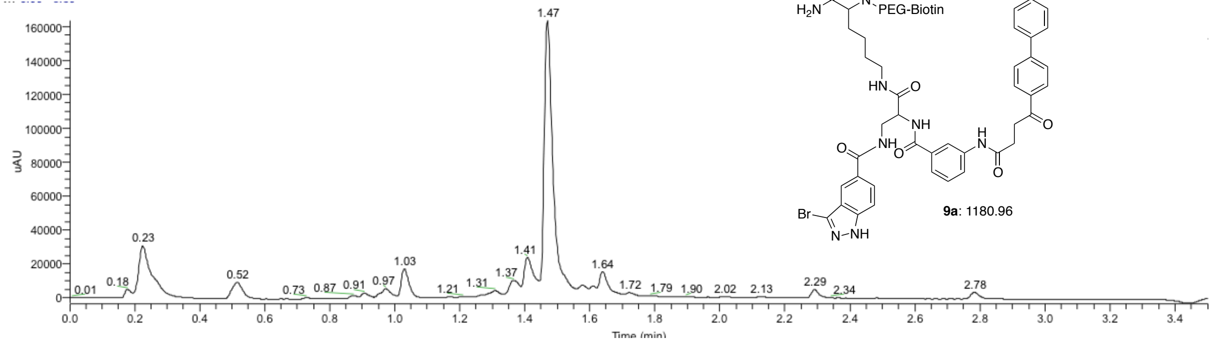

ESI (150-2000)

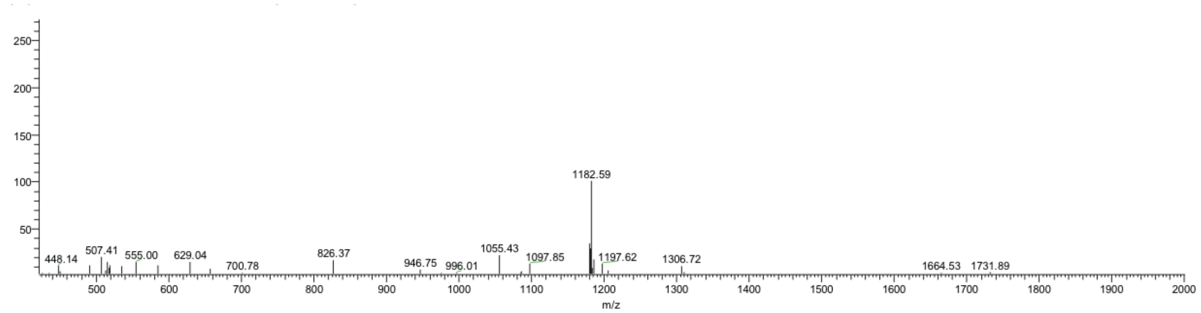

LC (Total Scan 190-800nm)

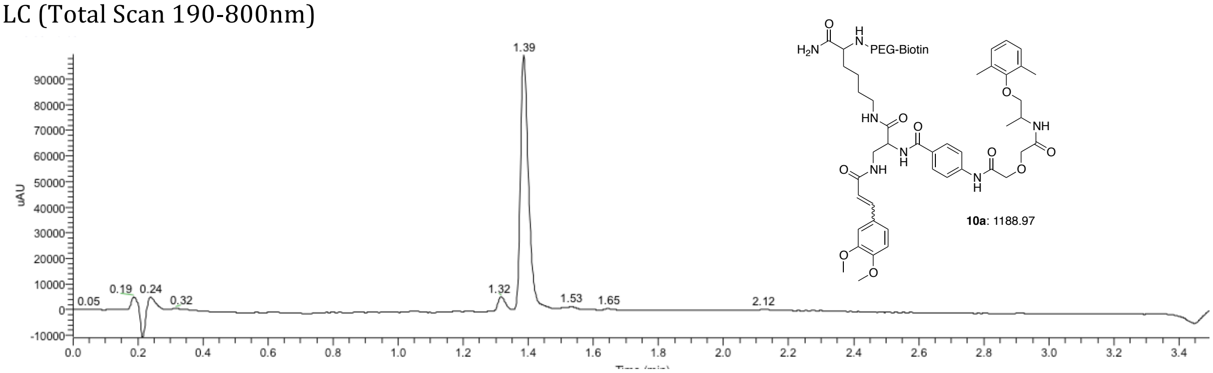

ESI (150-2000)

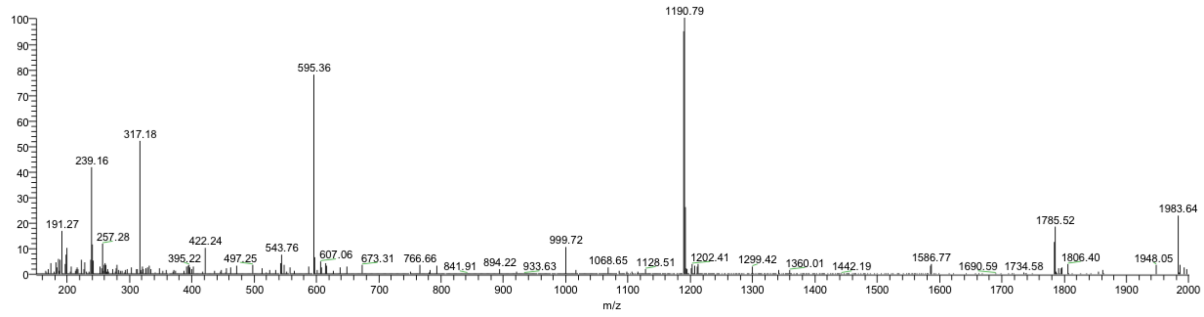

LC (Total Scan 190-800nm)

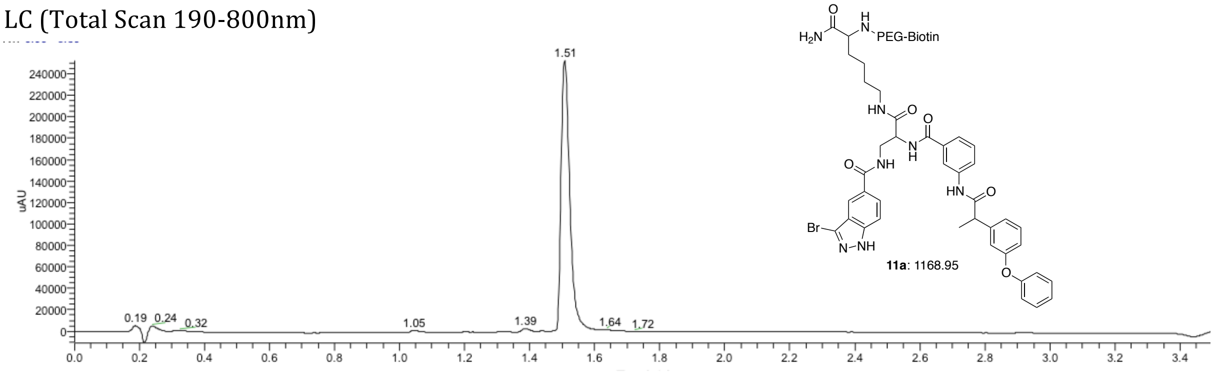

ESI (150-2000)

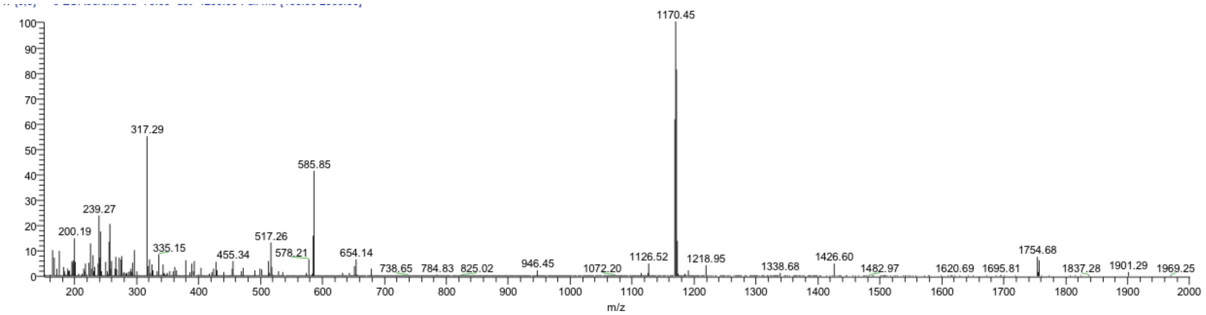

LC (Total Scan 190-800nm)

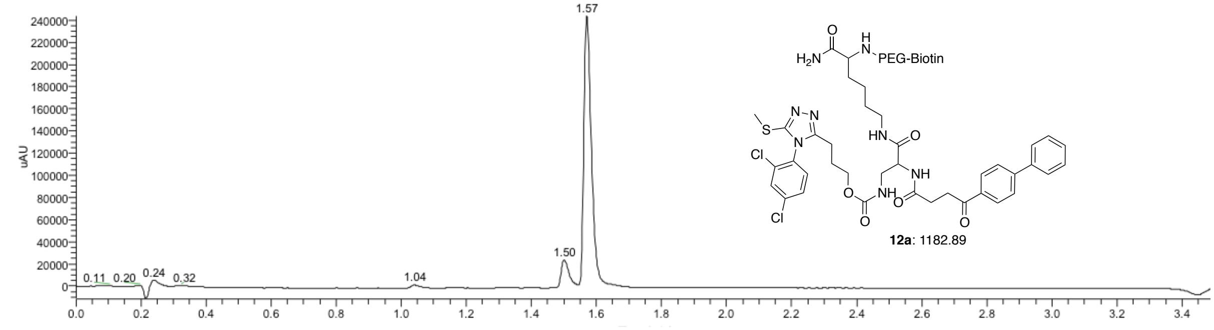

ESI (150-2000)

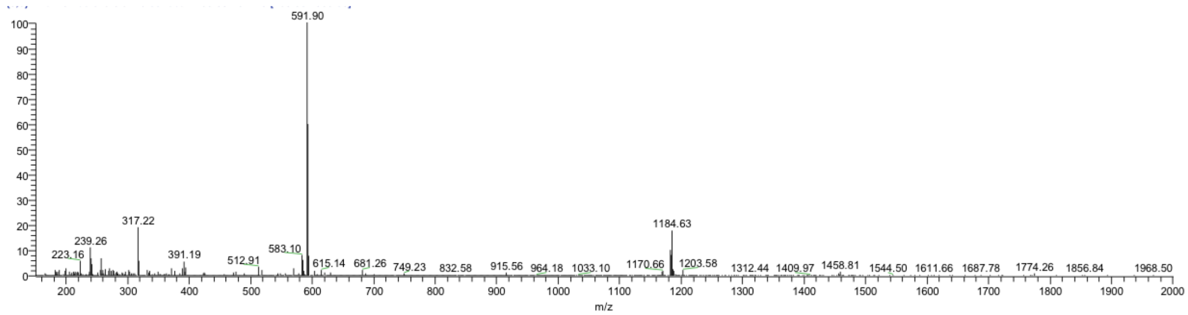

LC (Total Scan 190-800nm)

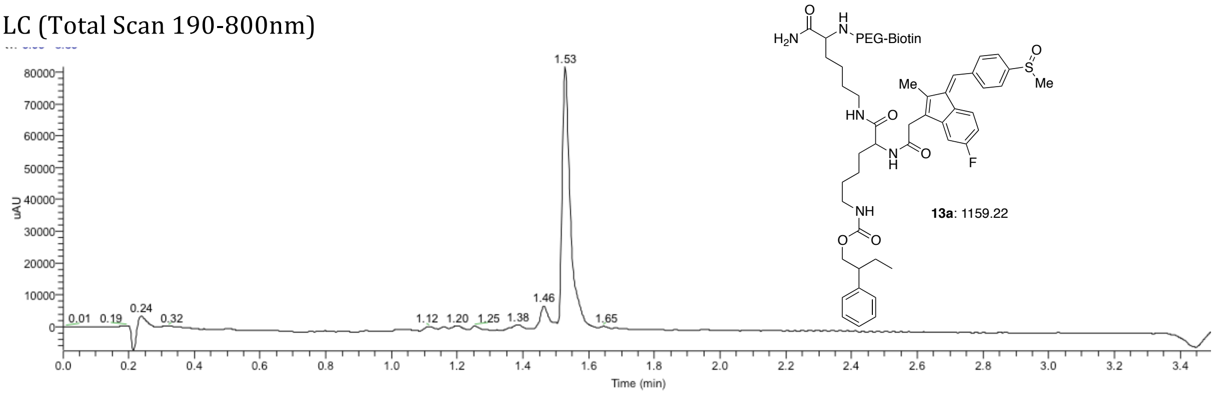

13a: 1159.22

ESI (150-2000)

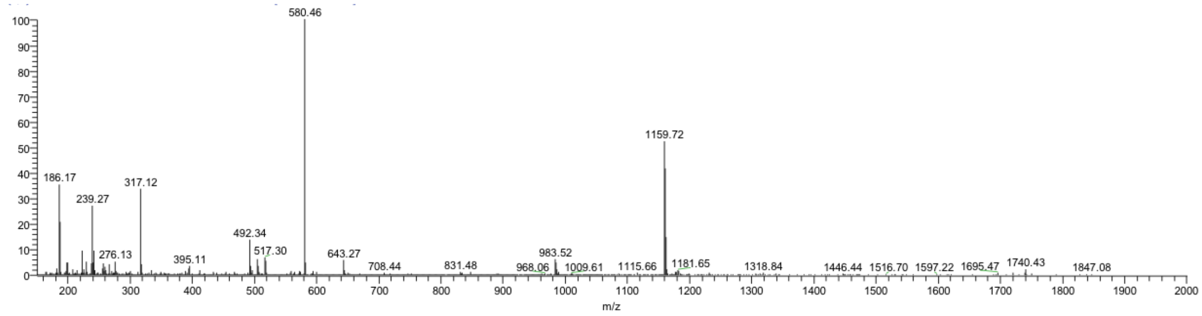

LC (Total Scan 190-800nm)

14a: 1127.96

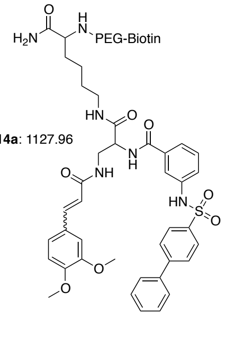

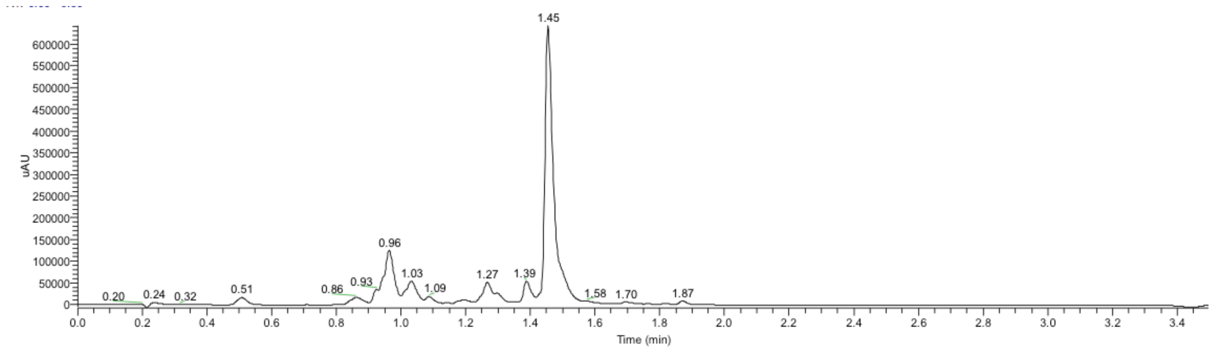

ESI (150-2000)

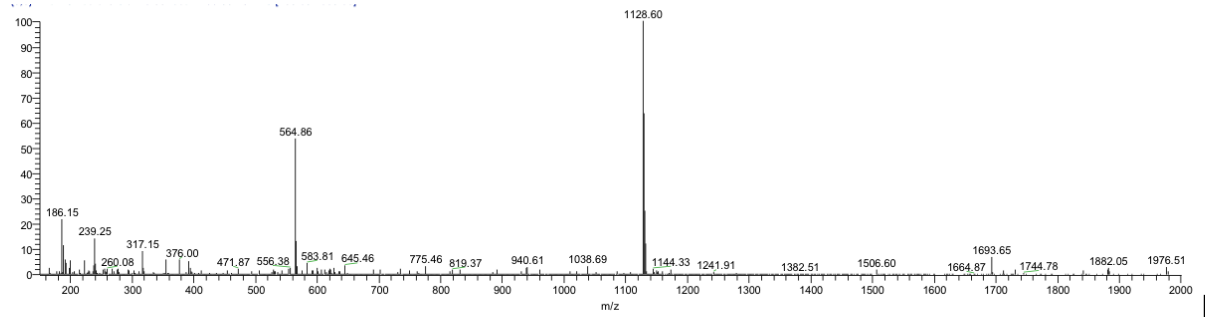

LC (Total Scan 190-800nm)

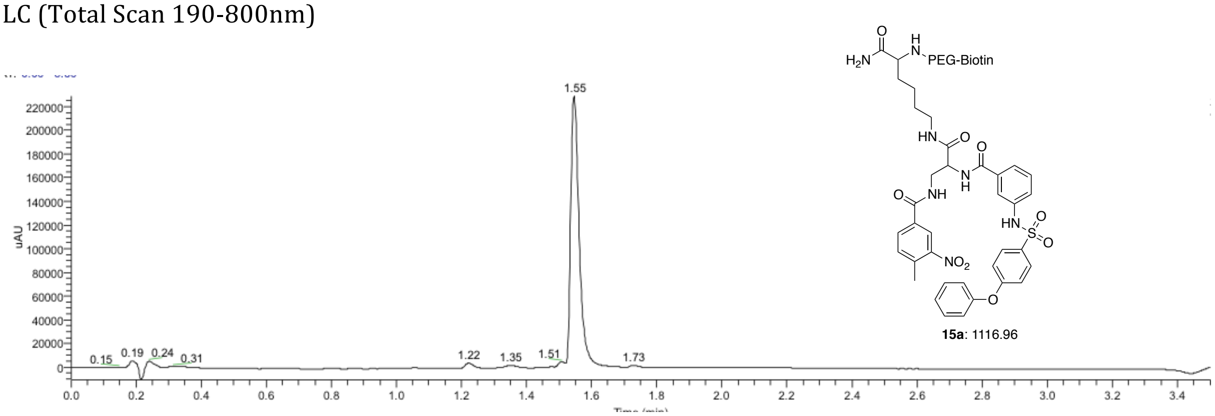

15a: 1116.96

ESI (150-2000)

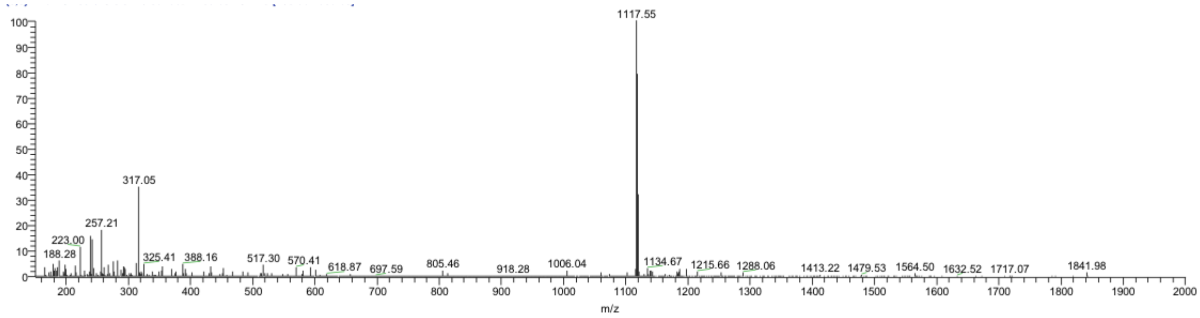

LC (Total Scan 190-800nm)

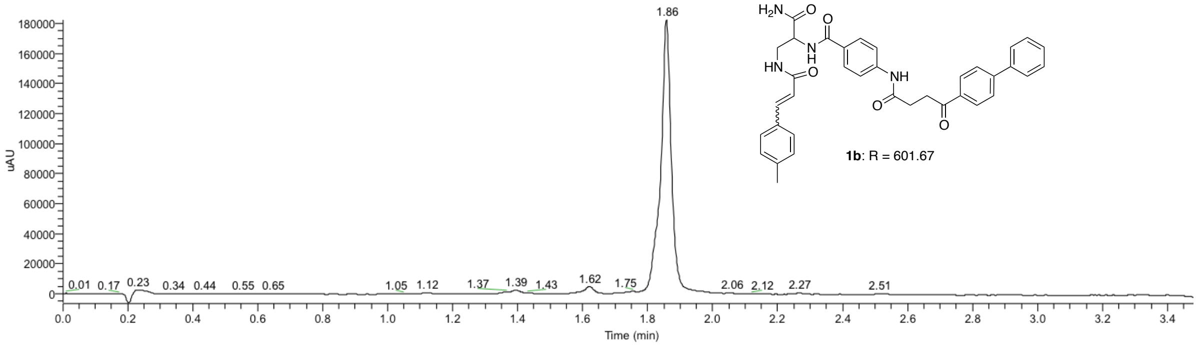

ESI (150-2000)

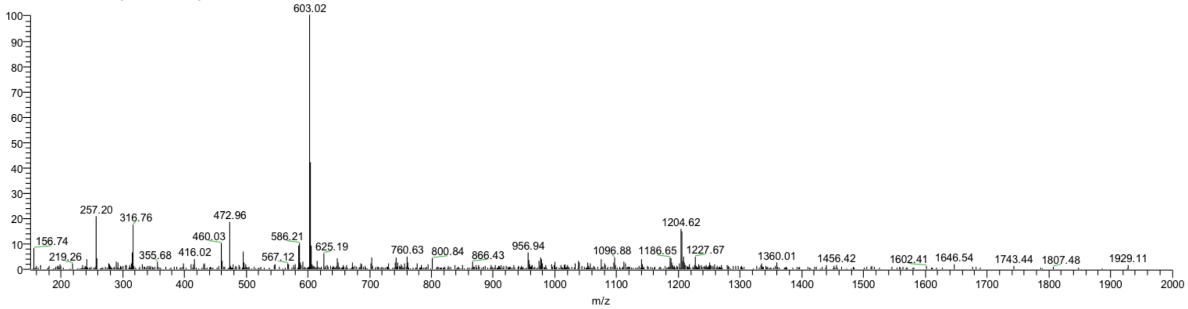

LC (Total Scan 190-800nm)

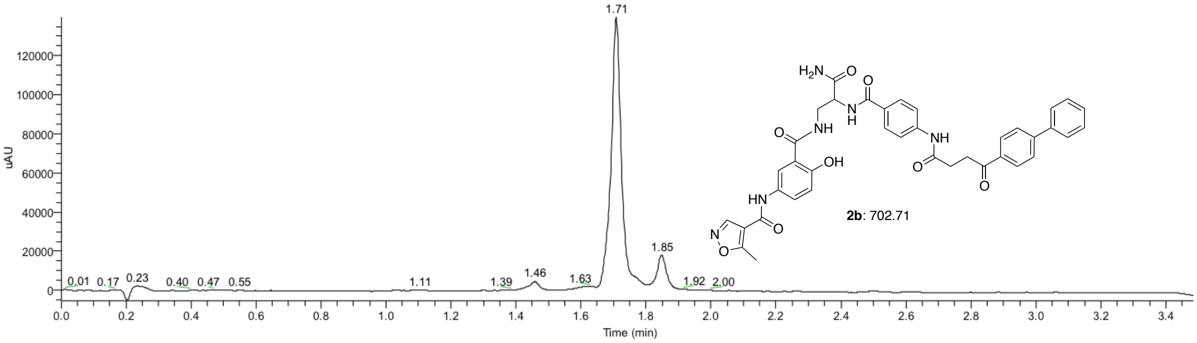

ESI (150-2000)

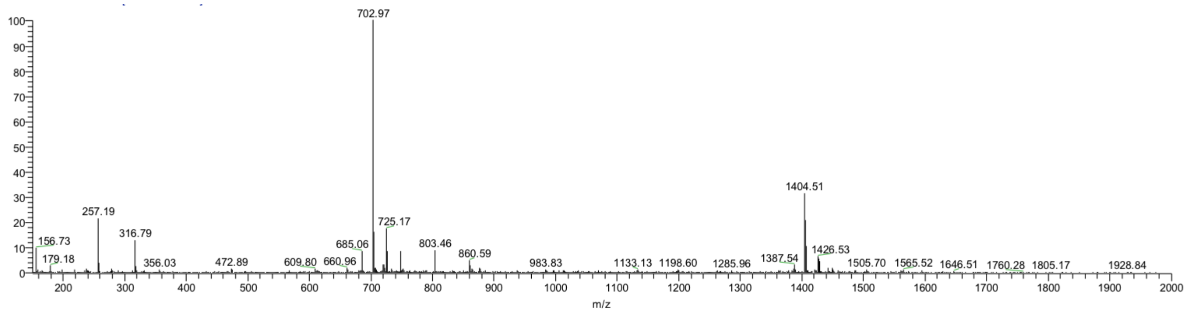

LC (Total Scan 190-800nm)

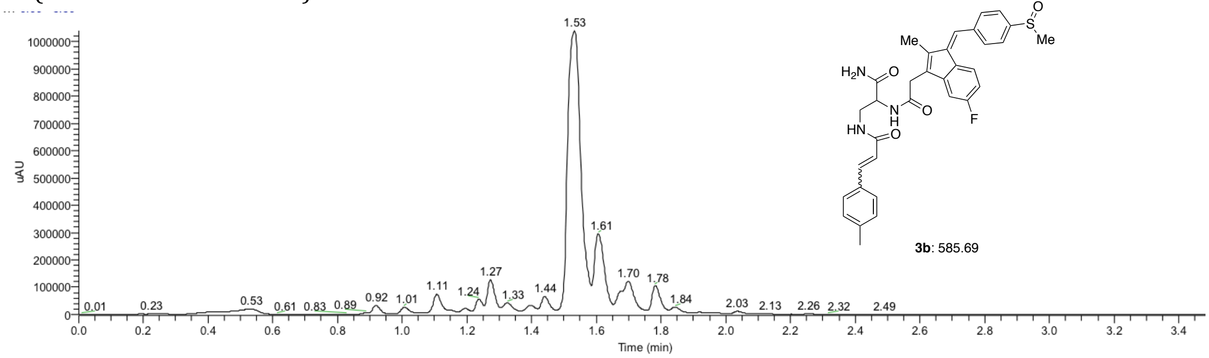

ESI (150-2000)

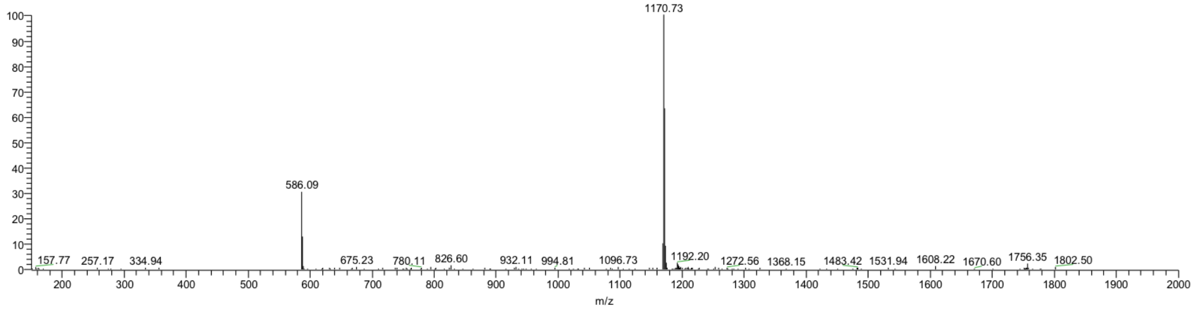

LC (Total Scan 190-800nm)

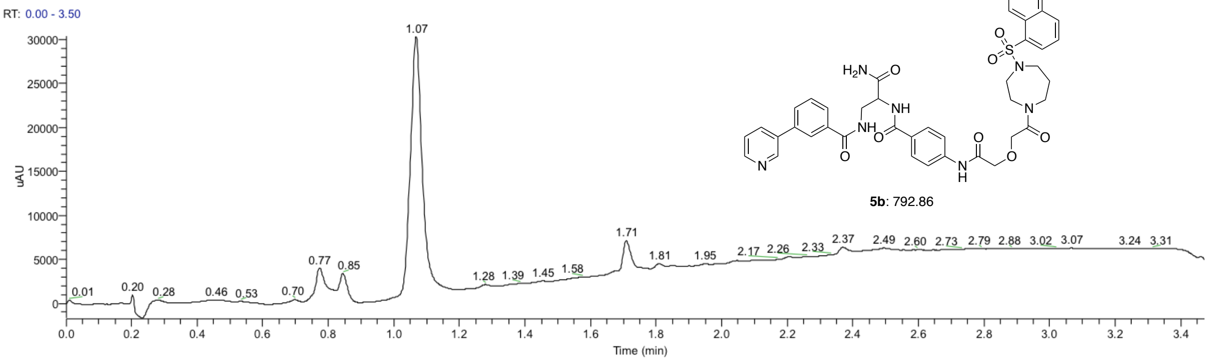

ESI (150-2000)

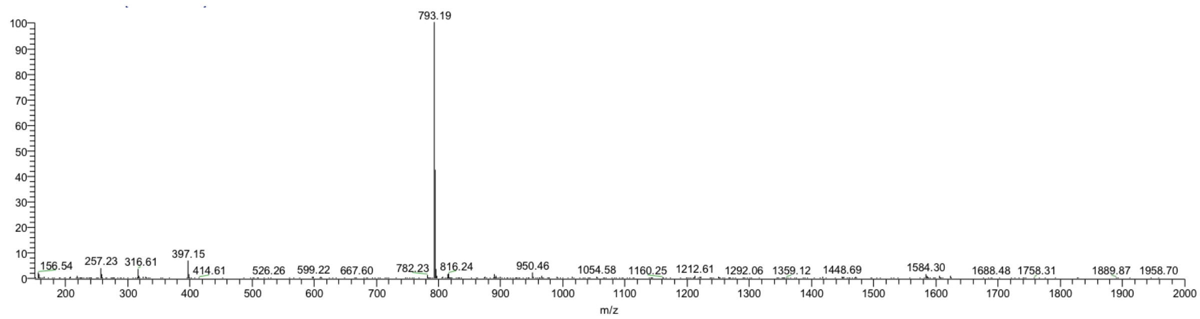

LC (Total Scan 190-800nm)

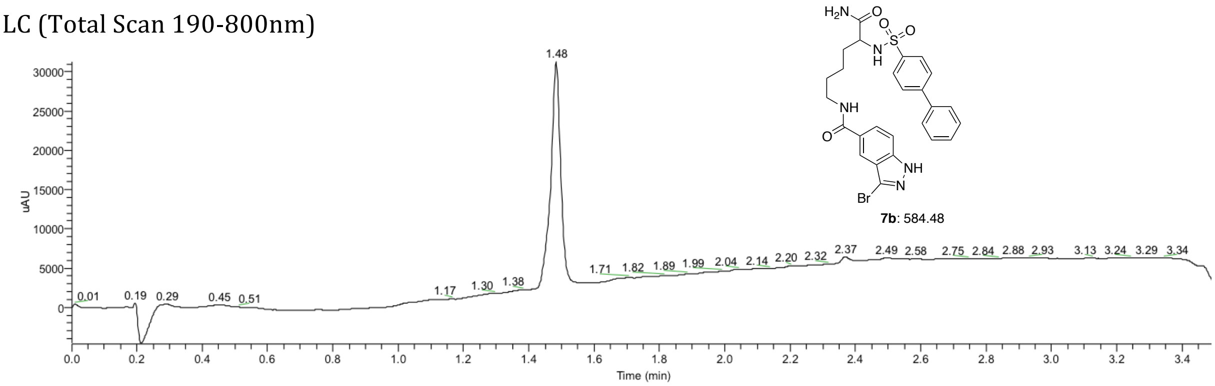

ESI (150-2000)

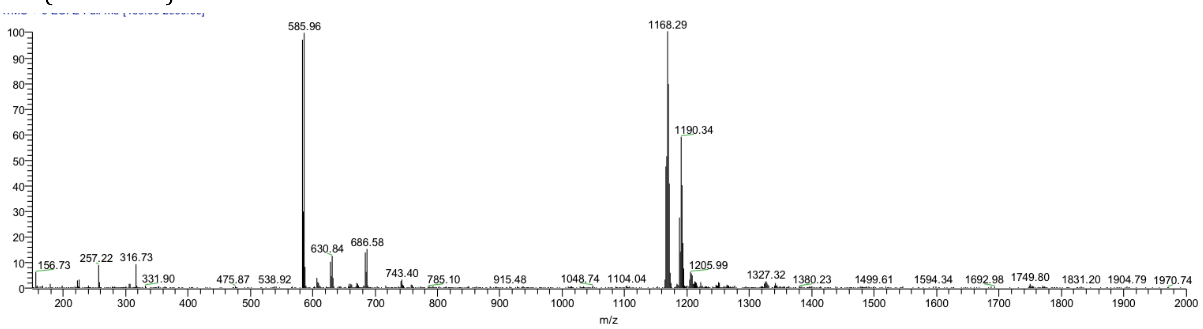

LC (Total Scan 190-800nm)

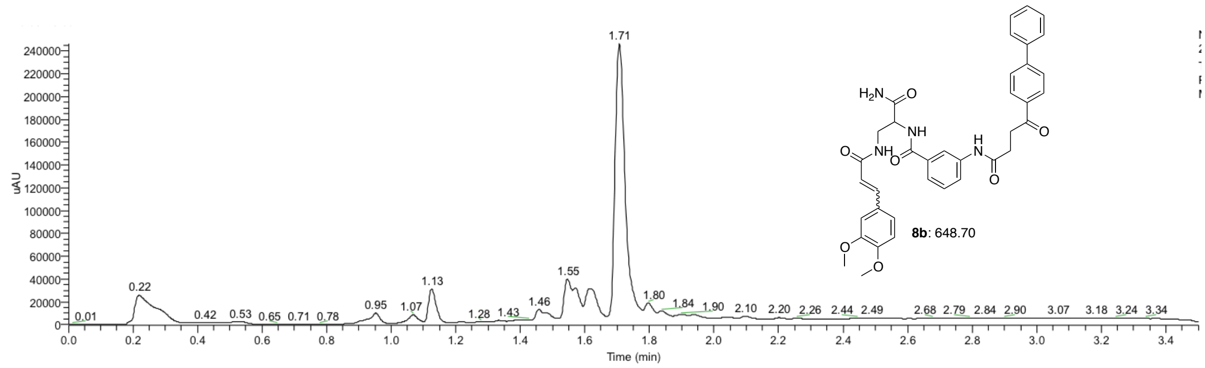

ESI (150-2000)

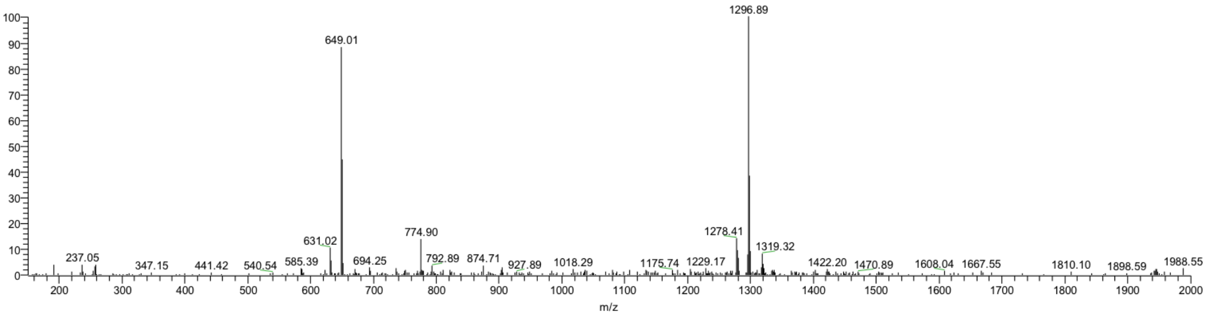

LC (Total Scan 190-800nm)

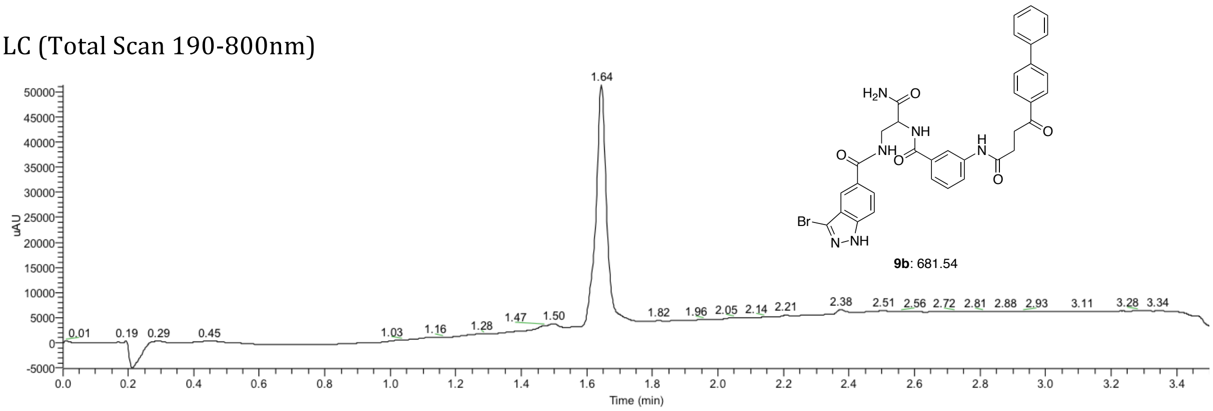

ESI (150-2000)

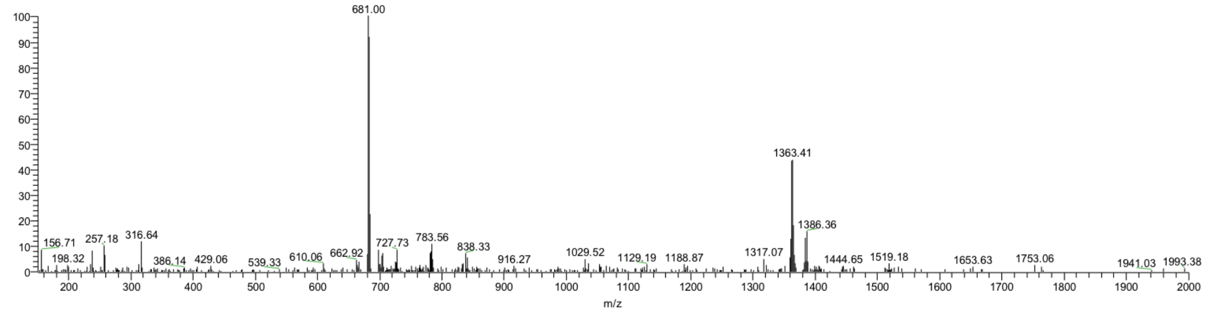

LC (Total Scan 190-800nm)

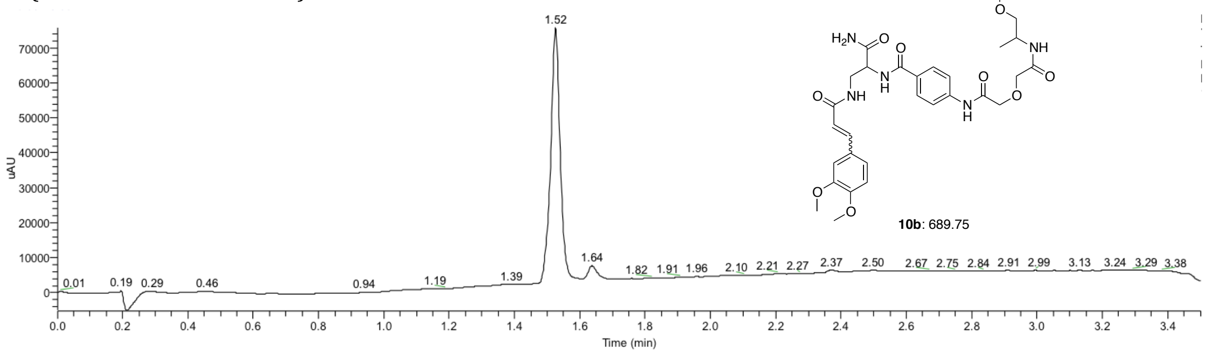

ESI (150-2000)

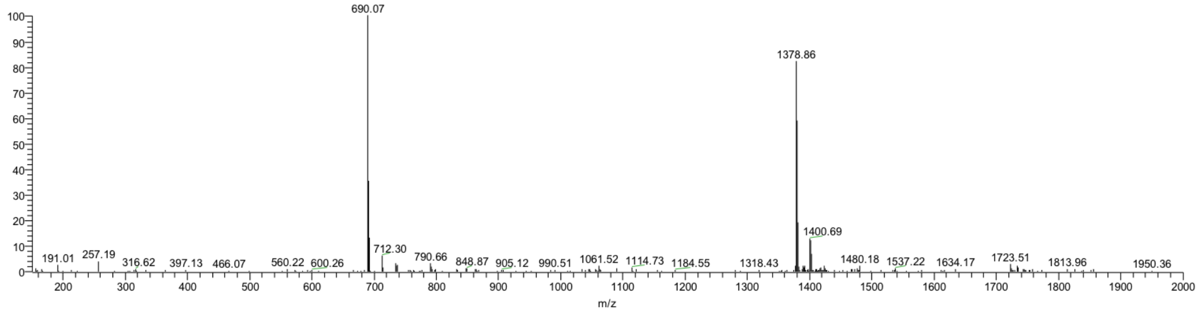

LC (Total Scan 190-800nm)

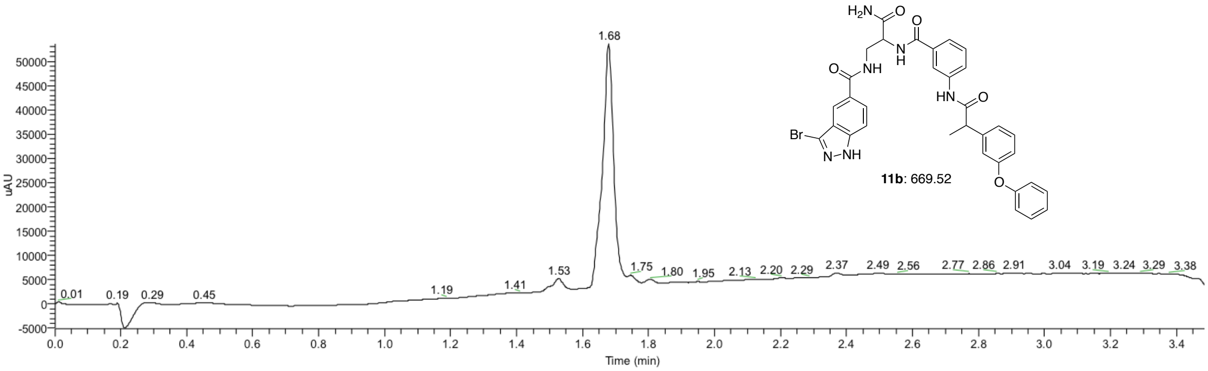

ESI (150-2000)

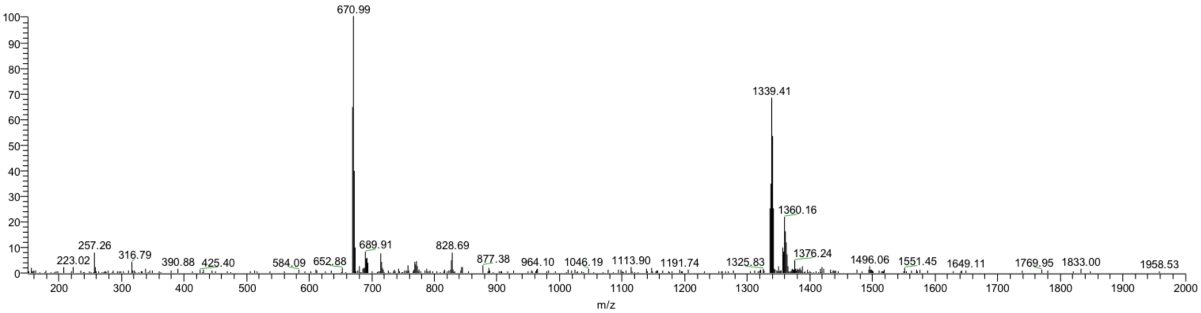

LC (Total Scan 190-800nm)

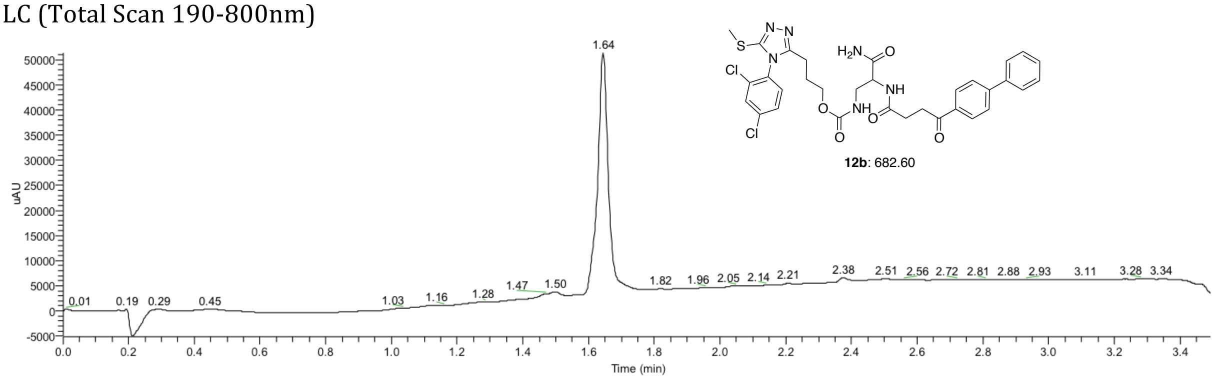

ESI (150-2000)

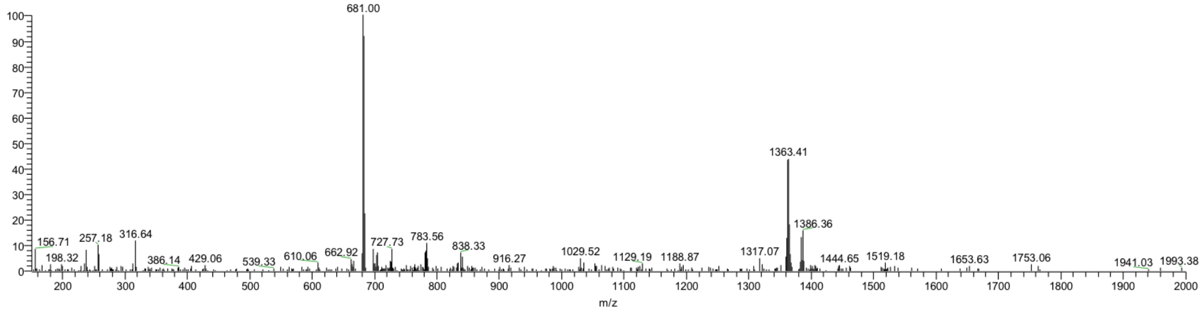

LC (Total Scan 190-800nm)

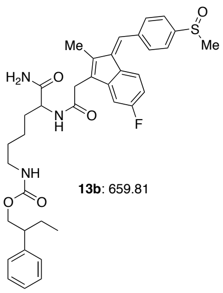

13b: 659.81

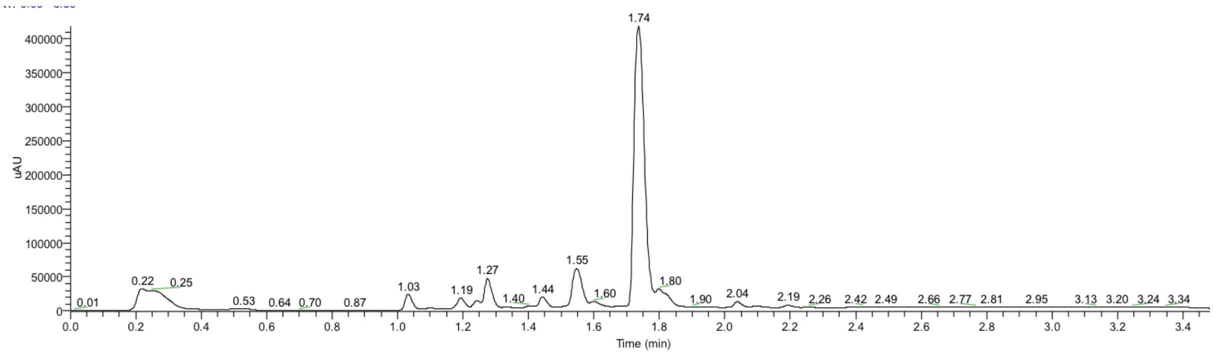

ESI (150-2000)

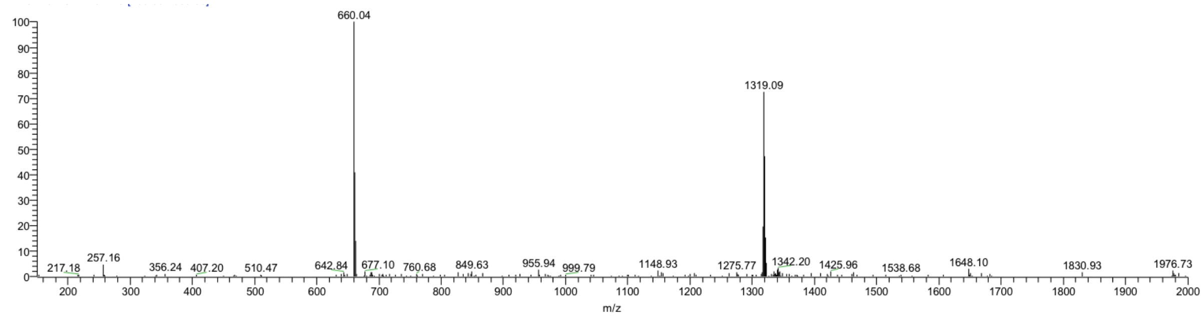

LC (Total Scan 190-800nm)

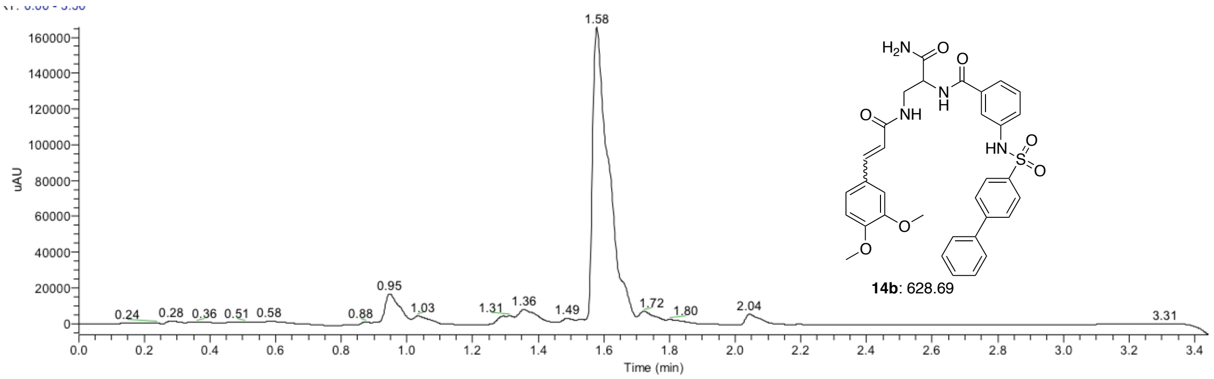

14b: 628.69

ESI (150-2000)

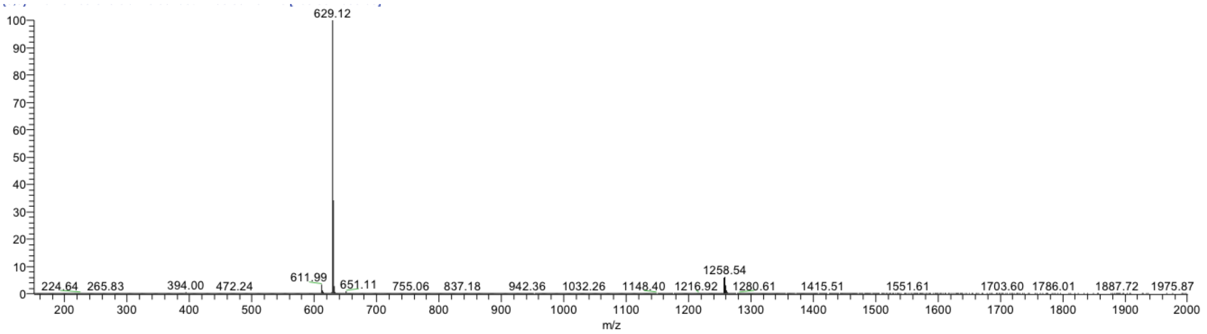

LC (Total Scan 190-800nm)

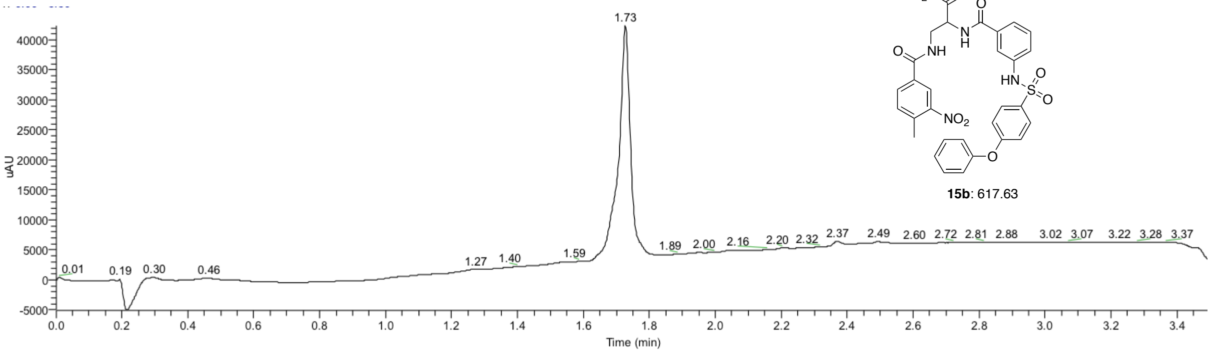

ESI (150-2000)

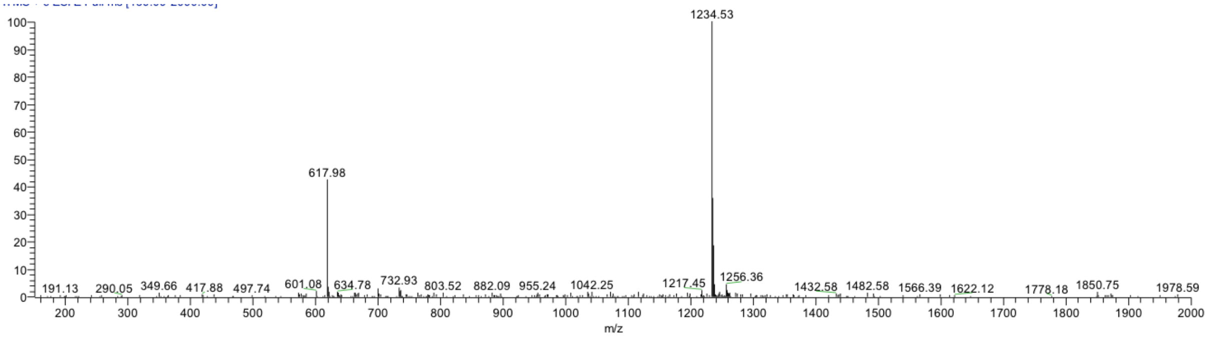

7. SPR and BLI affinity measurements of compounds 1-15.

CM5 sensor-chips (Biacore GE) were functionalized with streptavidin following the manufacturer's instructions. Biotynilated compounds **1a** to **15a** were then immobilized on the sensor chips by biotin-streptavidin coupling until stabilization of the baseline. Hsp70 and Hsp90 analytes were dissolved in 100mM Hepes, 50mM KCl, 5mM $MgCl_2$, 2.0mM DTT, at variable concentrations and injected at 20µL/min with an association/dissociation time of 3 min. The control channel used the same conditions but without loading of the test compound. Between injections the sensor-chip surface was regenerated by injection of 10µL, 10mM HCl. The association/dissociation constants were calculated using BiaEvaluation 4.1 software.

Compound	Loading (RU)	Compound	Codons	K_D Hsp70 (nM) [χ^2]	K_D Hsp90 (nM) [χ^2]
**1a**	101	**1a**	**6-67**	5.94 [8.9]	563 [9.1]
**2a**	86	**2a**	**6-95**	1.58 [1.8]	low binding
**3a**	54	**3a**	**44-67**	7.65 [0.28]	27 [0.28]
**4a**	130	**4a**	**44-28**	2.69 [0.69]	10.7 [4.8]
**5a**	147	**5a**	**5-96**	0.38 [6.3]	5.44 [55]
**6a**	136	**6a**	**74-28**	2.33 [4.8]	18.7 [4.3]
**7a**	215	**7a**	**74-89**	1.34 [8.2]	3.17 [25]
**8a**	697	**8a**	**8-64**	0.80 [11.2]	16.9 [34]
**9a**	611	**9a**	**8-89**	0.64 [34]	14.7 [23]
**10a**	178	**10a**	**45-64**	1.88 [9.3]	2.97 [3.6]
**11a**	805	**11a**	**32-89**	12.10 [3.7]	5.6 [40]
**12a**	698	**12a**	**4-28**	2.02 [4.11]	10.1 [3.3]
**13a**	552	**13a**	**42-27**	1.16 [93]	29.5 [75]
**14a**	843	**14a**	**80-64**	2.88 [9.6]	52.8 [46]
**15a**	1241	**15a**	**72-66**	1.30 [11.5]	35.3 [52]

1a Hsp70 (2.8nM, 5.6nM, 11nM, 22nM, 44nM)

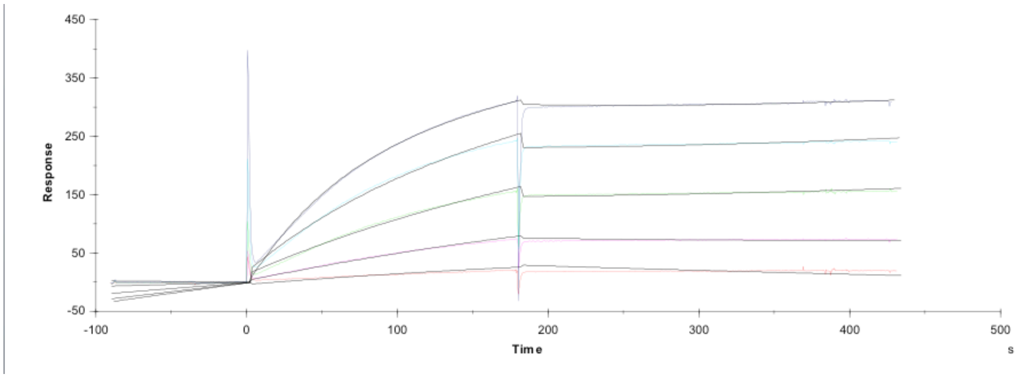

1a Hsp90 (62nM, 124nM, 248nM, 496nM, 1nM)

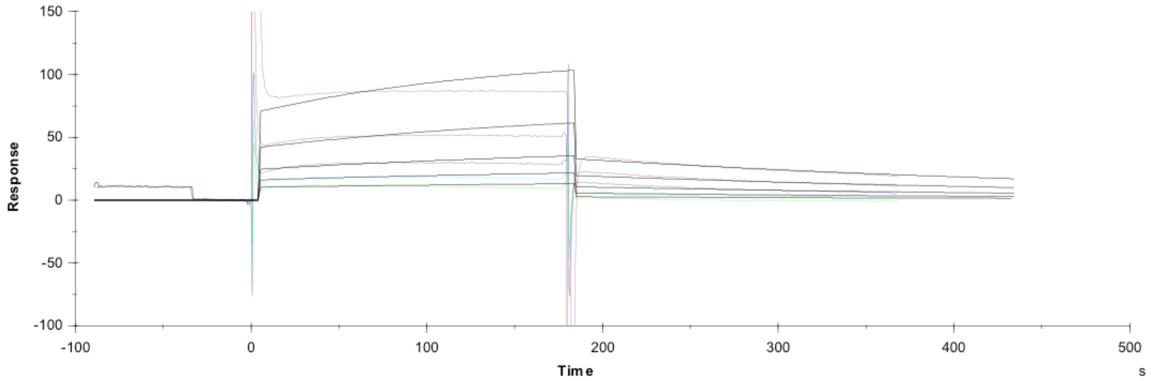

2a Hsp70 (2.8nM, 5.6nM, 11nM, 22nM, 44nM)

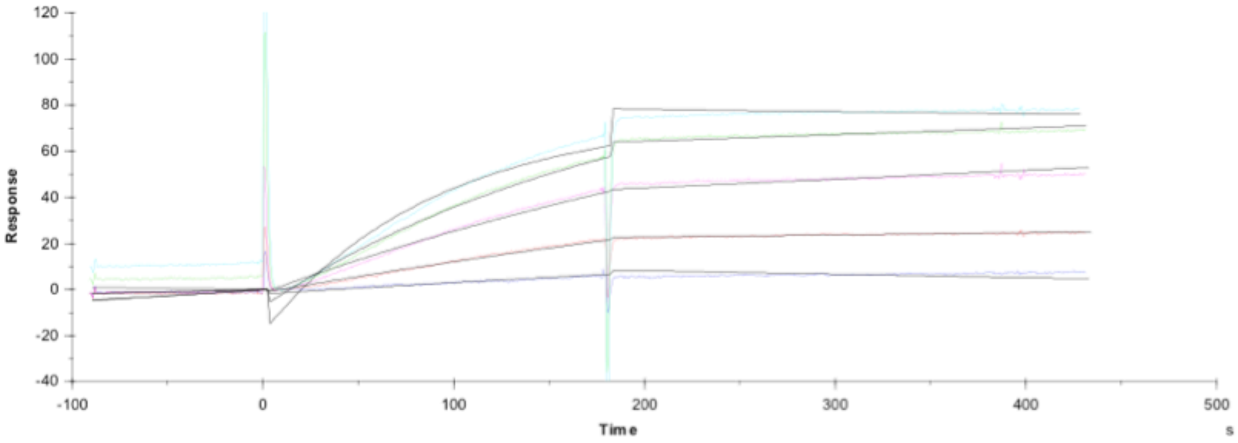

2a Hsp90 (125nM, 250nM, 500nM)

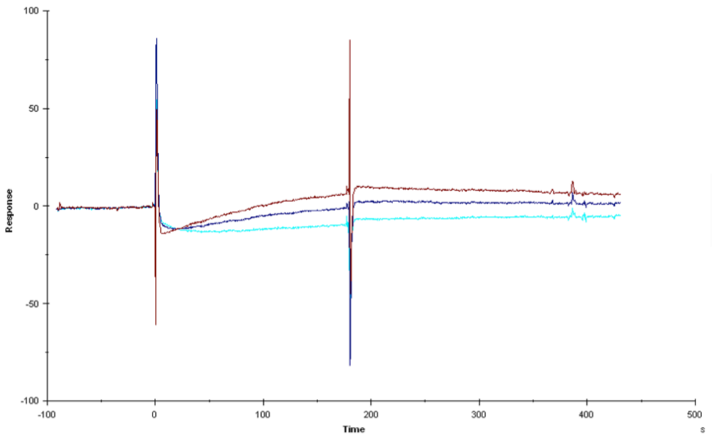

3a Hsp70 (2.8nM, 5.6nM, 11nM, 22nM, 44nM)

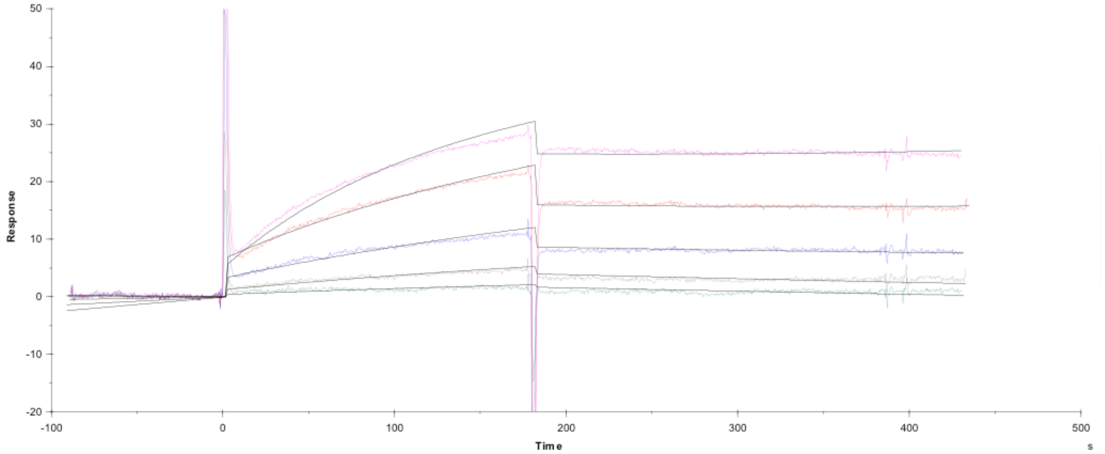

3a Hsp90 (16nM, 32nM, 64nM, 125nM, 250nM)

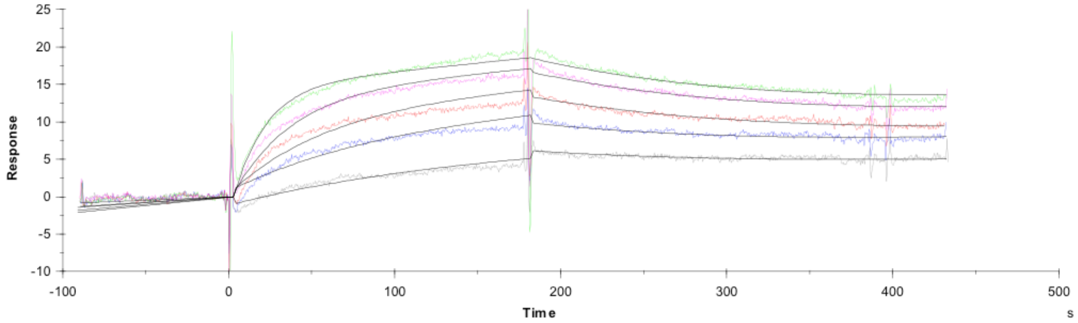

4a Hsp70 (5.6nM, 11nM, 22nM, 44nM, 89nM)

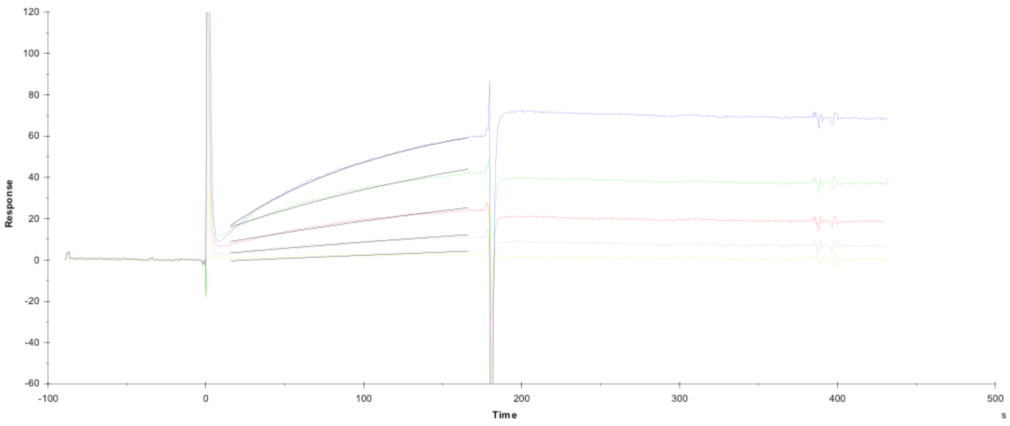

4a Hsp90 (2nM, 4nM, 8nM, 16nM, 31nM, 62nM, 125nM)

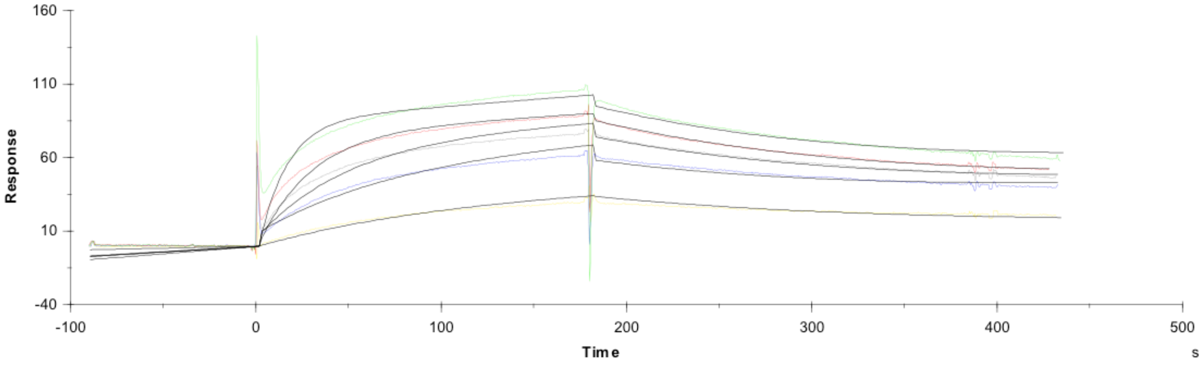

5a Hsp70 (1.4 nM, 2.8nM, 5.6nM, 11nM, 22nM, 44nM, 88nM)

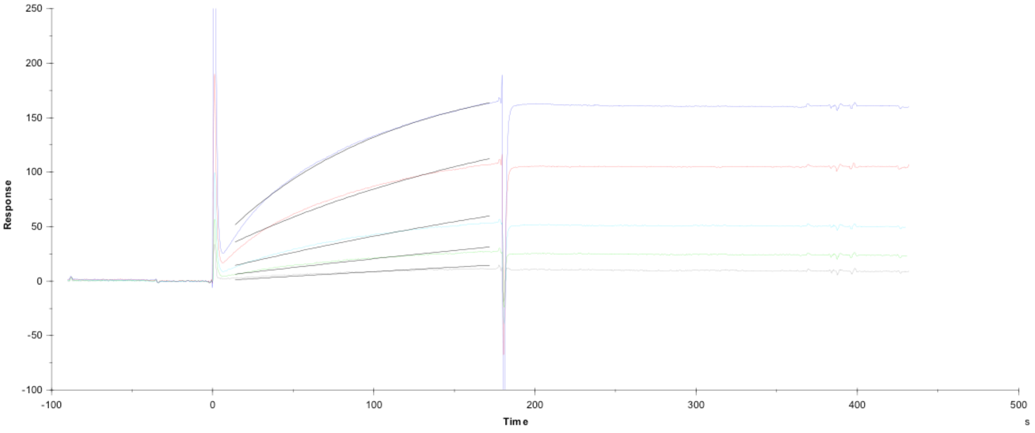

5a Hsp90 (16nM, 31nM, 62nM, 125nM, 250nM)

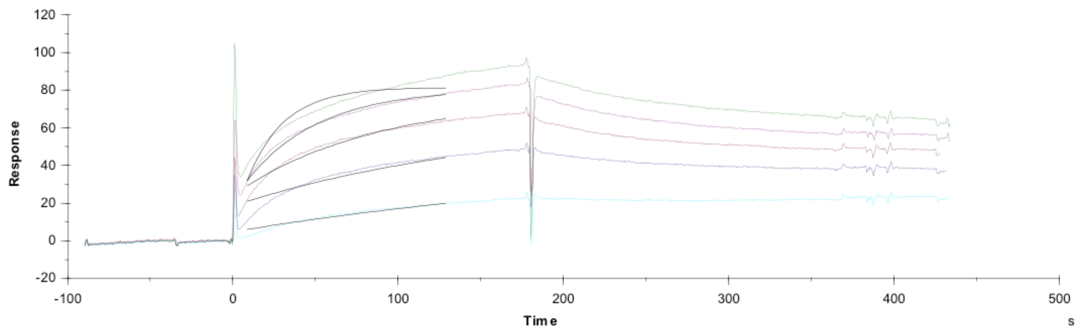

6a Hsp70 (5.6nM, 11nM, 22nM, 44nM, 89nM, 178nM)

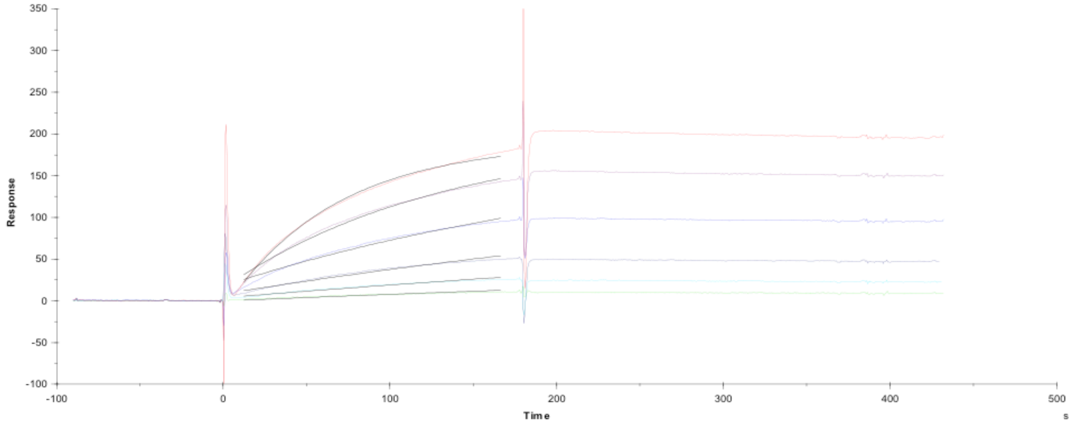

6a Hsp90 (16nM, 31nM, 62nM, 125nM, 250nM)

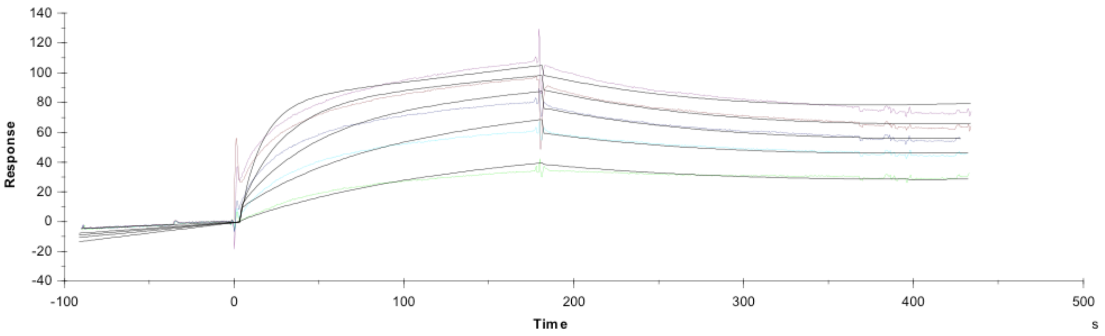

7a Hsp70 (1.4nM, 2.8nM, 5.6nM, 11nM, 22nM)

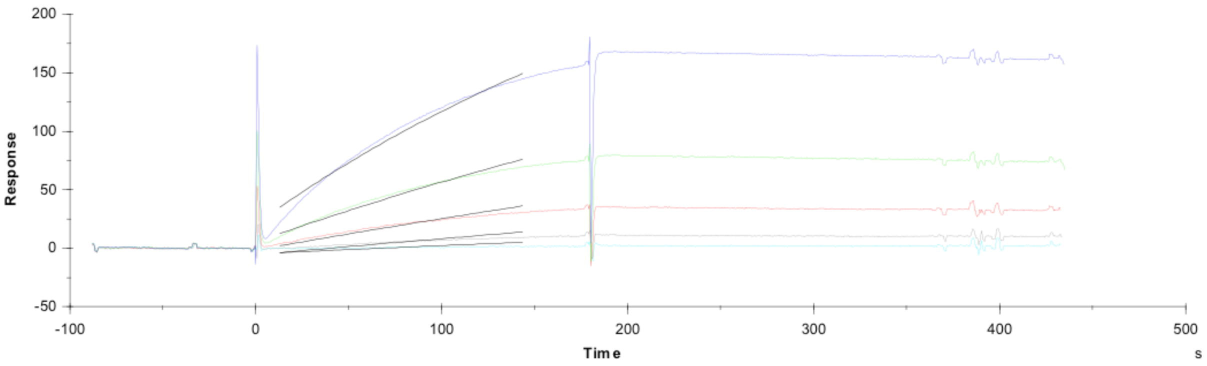

7a Hsp90 (8.0nM 16nM, 31nM, 62nM, 125nM)

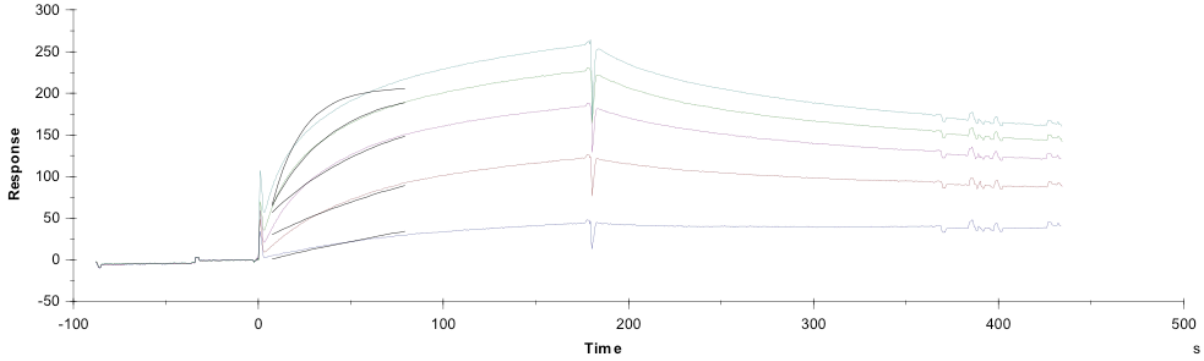

8a Hsp70 (2.8nM, 5.6nM, 11nM, 22nM, 44nM)

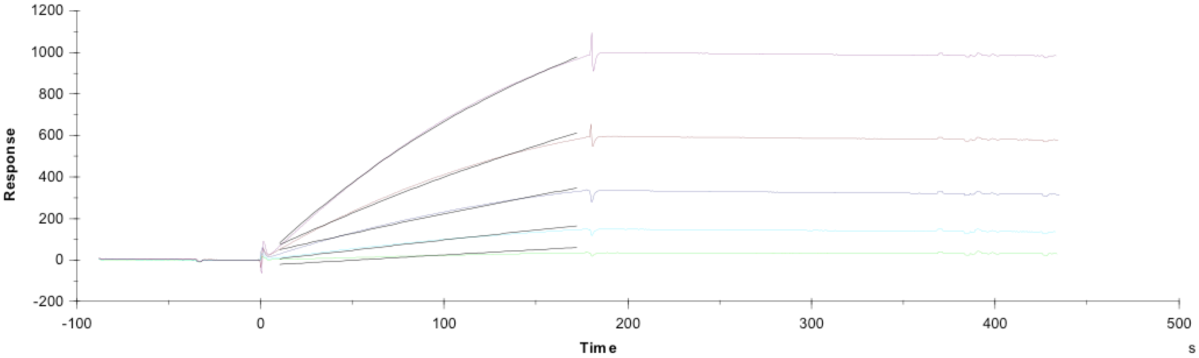

8a Hsp90 (31nM, 62nM, 125nM, 250nM, 500nM)

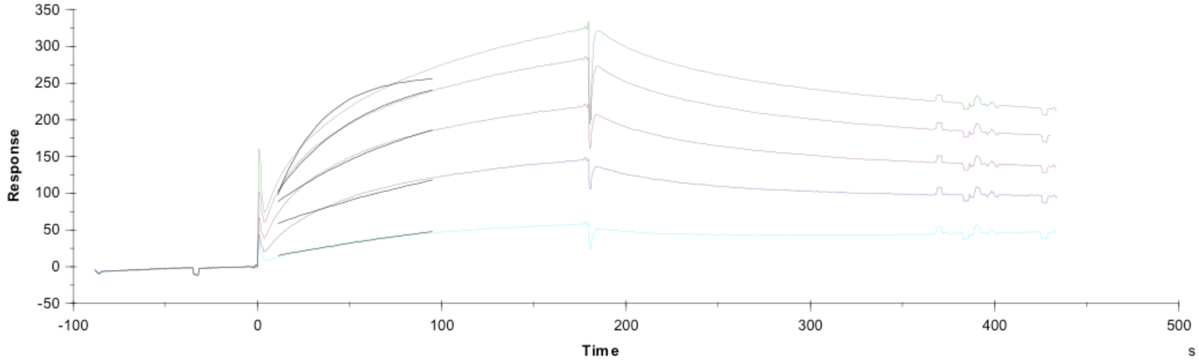

9a Hsp70 (2.8nM, 5.6nM, 11nM, 22nM, 44nM)

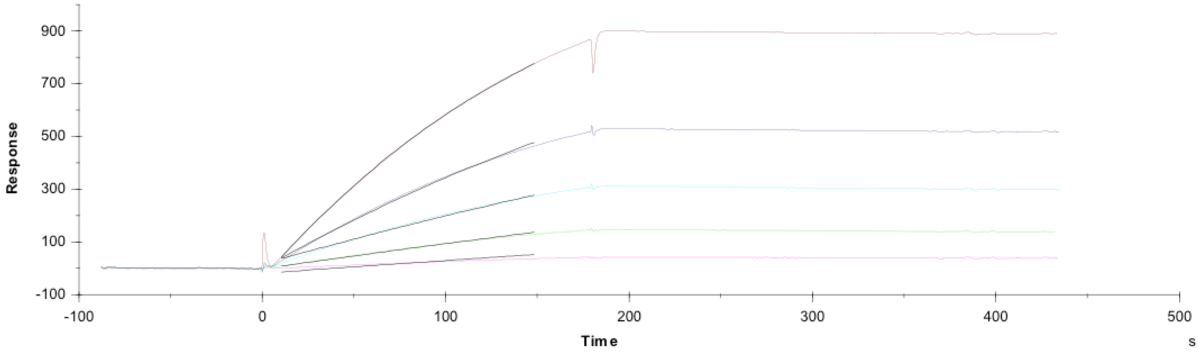

9a Hsp90 (31nM, 62nM, 125nM, 250nM, 500nM)

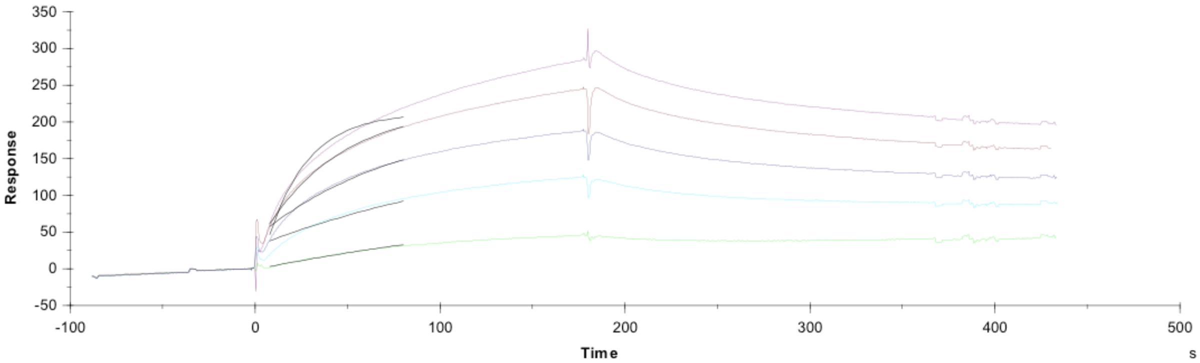

10a Hsp70 (5.6nM, 11nM, 22nM, 44nM, 89nM)

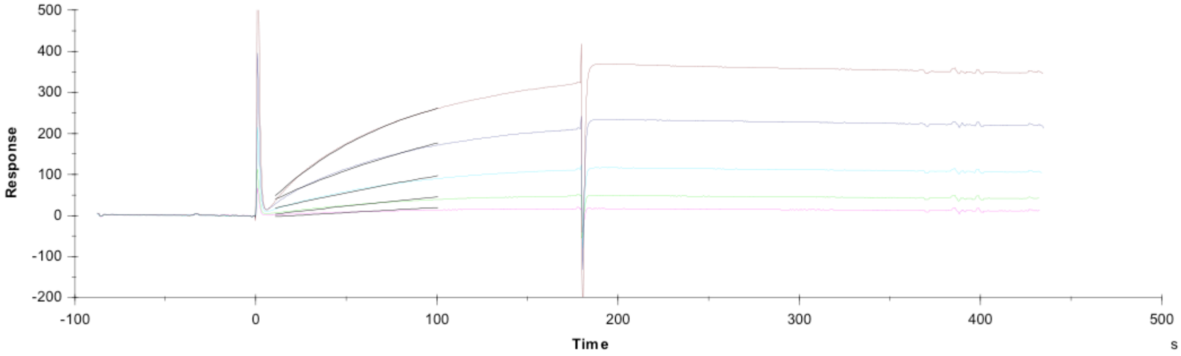

10a Hsp90 (8.0nM 16nM, 31nM, 62nM, 125nM)

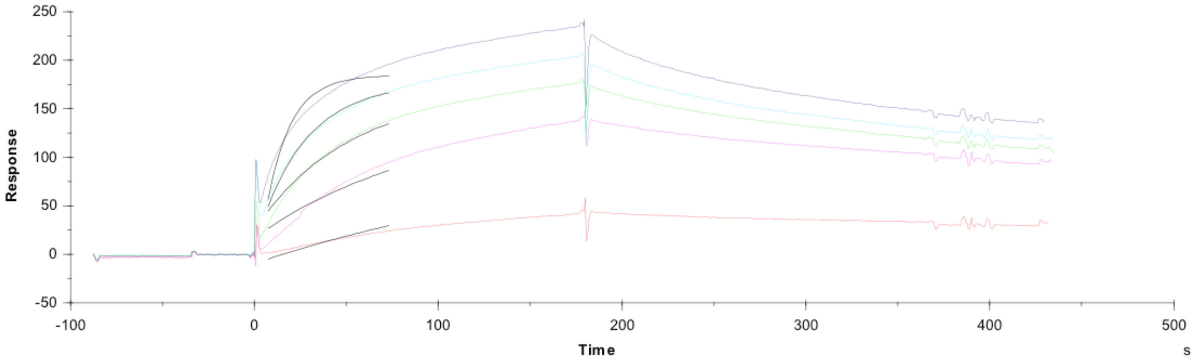

11a Hsp70 (1.4nM, 2.8nM, 5.6nM, 11nM, 22nM)

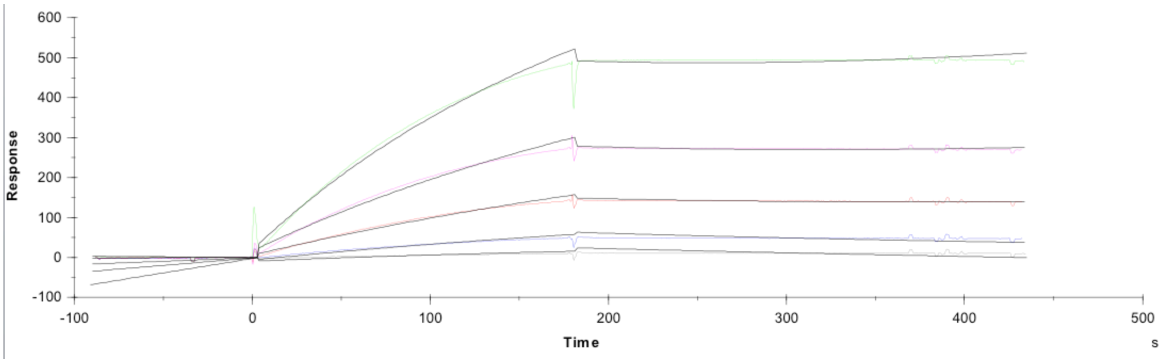

11a Hsp90 (31nM, 62nM, 125nM, 250nM, 500nM)

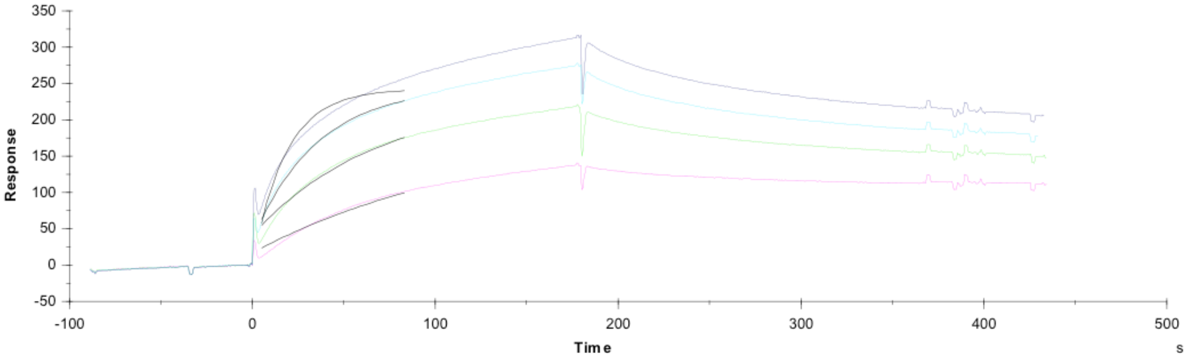

12a Hsp70 (2.8nM, 5.6nM, 11nM, 22nM, 44nM)

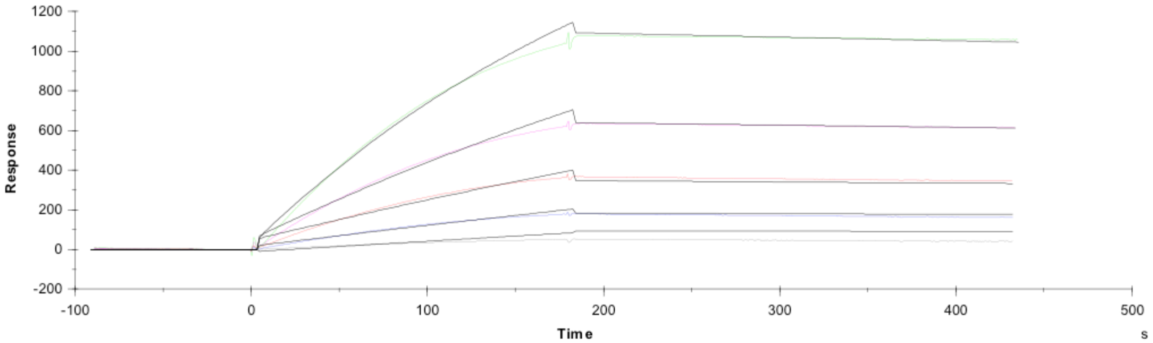

12a Hsp90 (31nM, 62nM, 125nM, 250nM, 500nM)

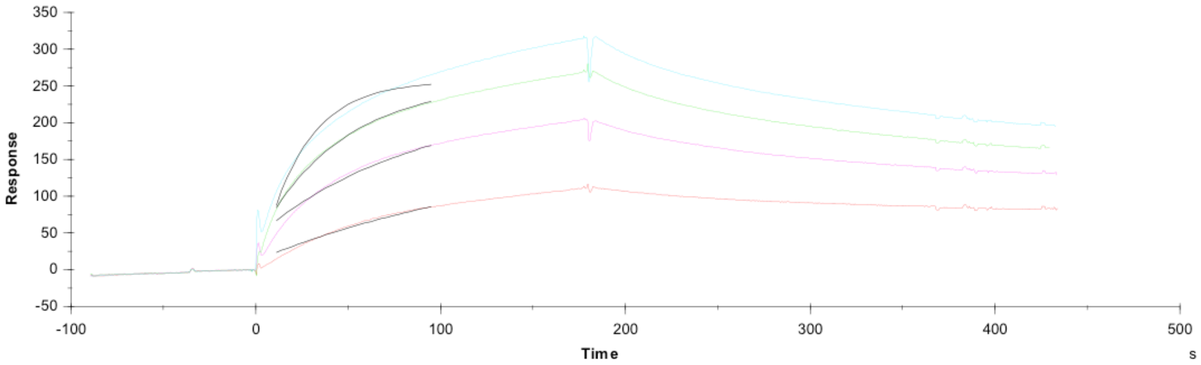

13a Hsp70 (2.8nM, 5.6nM, 11nM, 22nM, 44nM)

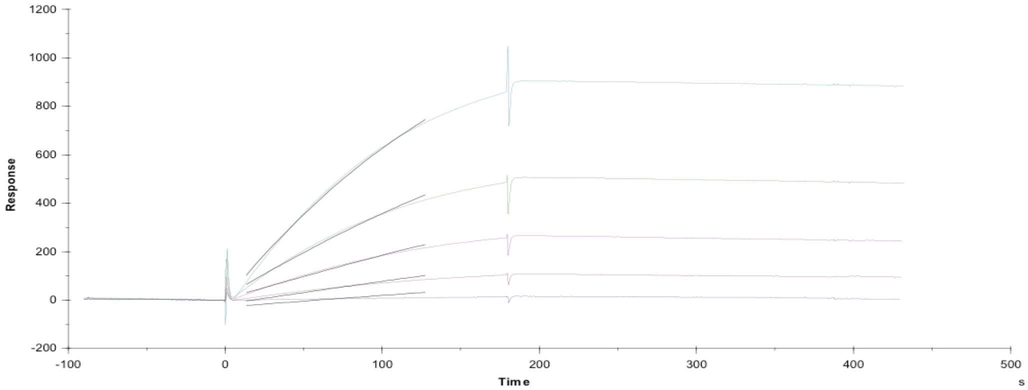

13a Hsp90 (16nM, 31nM, 62nM, 125nM, 250nM)

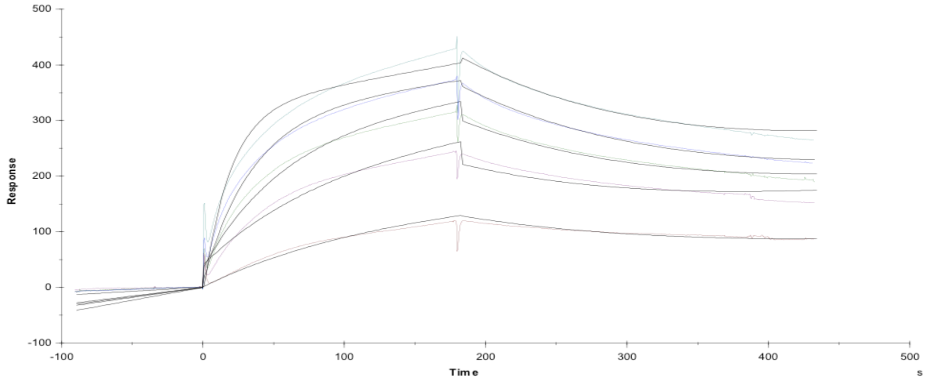

14a Hsp70 (5.6nM, 11nM, 22nM, 44nM, 89nM)

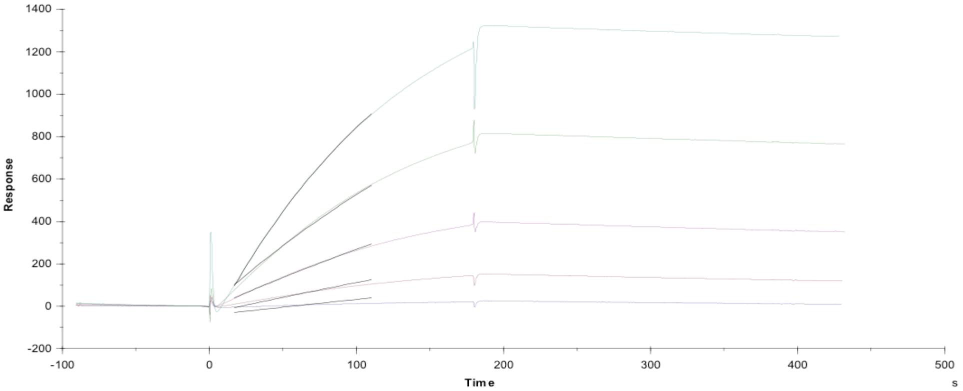

14a Hsp90 (31nM, 62nM, 125nM, 250nM, 500nM)

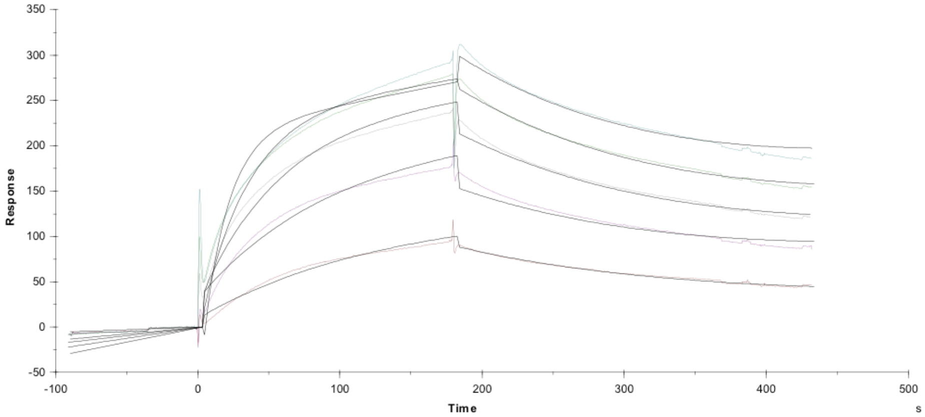

15a Hsp70 (2.8nM, 5.6nM, 11nM, 22nM, 44nM)

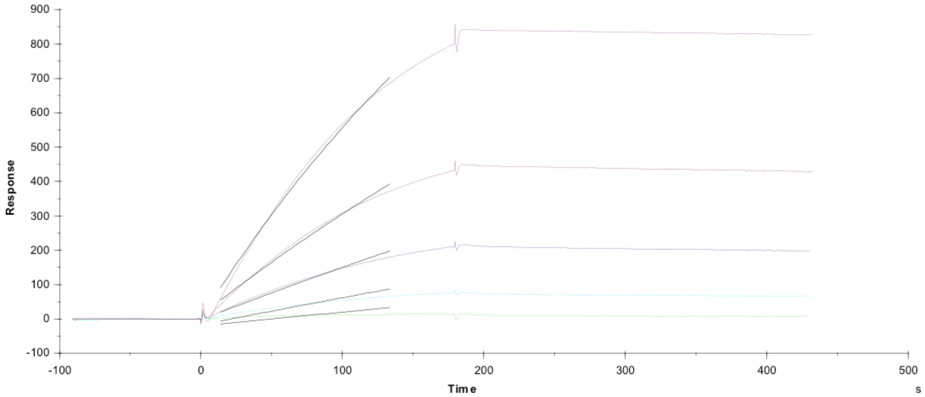

15a Hsp90 (16nM, 31nM, 62nM, 125nM, 250nM)

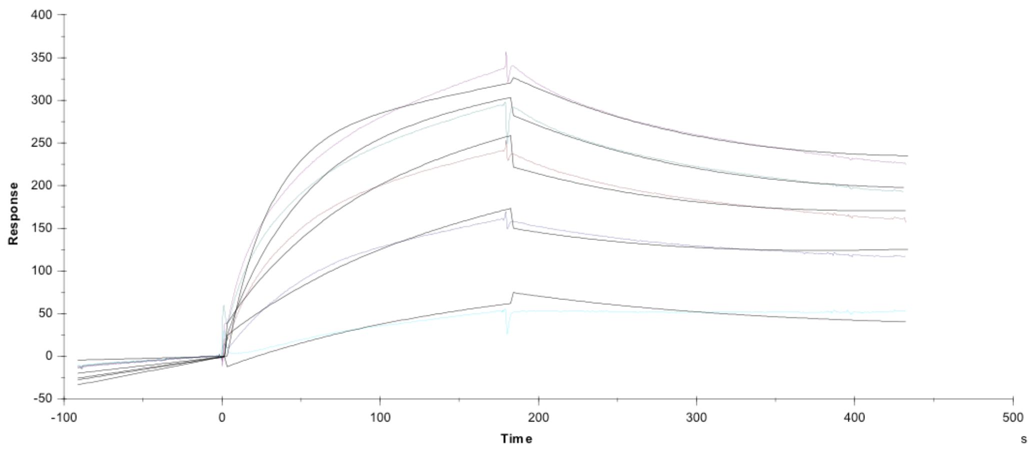

Biolayer interferometry (BLI) measurements to assess the binding of Hsp 70 to small molecule ligands

Biotin conjugates **1a** and **2b** were dissolved at 10 µM in 25mM HEPES, 50mM KCl - 5mM $MgCl_2$ - 20 mM NaCl - 0.05% Tween 20 buffer. Streptavidin functionalized Biosensors (Fortebio Pall, Menlo Park, Ca) were rehydrated in the same buffer for 10 min. The biosensor was loaded by immersed in a solution containing the biotin conjugate during 120s. The solution was changed to buffer alone and the baseline was acquired for 60 s. The biosensor tip was then immersed in a solution containing the Hsp70-his (Novus Biologicals) at different concentrations. The response was recorded for 120s. The response to dissociation was recorded by submerging the biosensor tip in the buffer for 120s. Kinetic constants were derived using Blitz Pro Software analysis. The competition experiments were carried out in the same conditions, pre-incubating Hsp70 with the non biotinylated small molecule (**1b**) for 15 min at 37° C prior to the association phase.

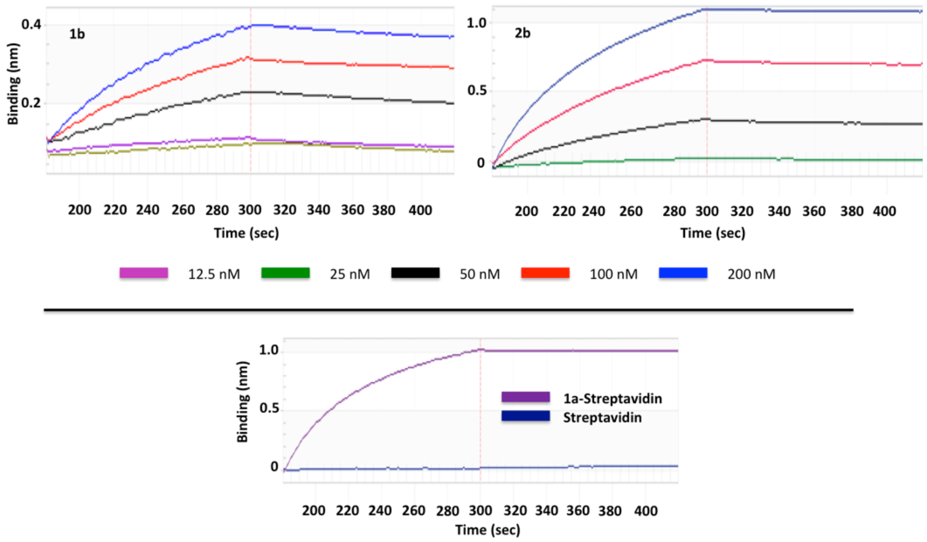

Dose response curve for binding of hsp70 to immobilized **1a** and **2a** with streptavidin biosensor by bio-layer interferometry (BLItz). Measured K_D for **1b**: 7.4 nM; Measured K_D for **2b**: 3.97 nM (top). Plot showing the curve for the binding of HSP70 at 400 nM to the Streptavidin biosensor vs the binding of HSP70 at the same concentration into **1a** immobilized on the same biosensor (bottom).

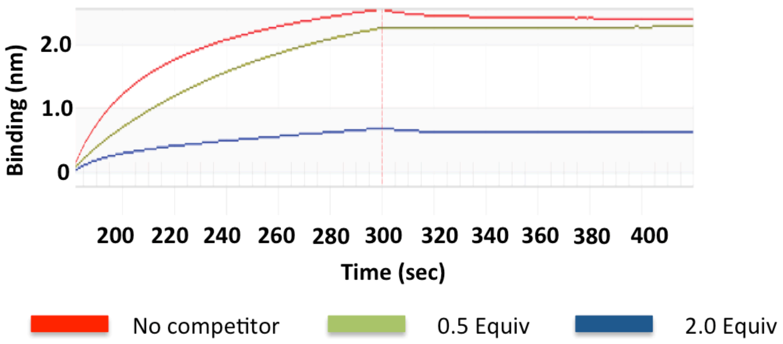

Competition experiment with **1b** at different molar equivalence to Hsp70 for immobilized **1a**.

8. Protein pull-down with immobilized ligand

2 nmol of biotinylated compounds diluted in PBS were incubated with 50 µl of streptavidin coated magnetic beads (Dynal). After 20 min of immobilization, the beads were washed three times with 10 mM Tris-HCl , pH 7, 2M NaCl and finally the beads were equilibrated with screening buffer [200 mM HEPES pH 7.4, 0.5 M KCl, 50 mM $MgCl_2$, 200 mM Na_2MO_4, 0.1% Tergitol, 2 mM DTT]. Hsp70-GST or Hsp90 (Stressgen) were diluted to 300 nM in the screnning buffer and incubated with the streptavidin coated magnetic beads, functionalized with the biotinylated compounds as well as a negative control functionalized with biotin. After 1 hour of incubation with gentle agitation at room temperature, the magnetic beads were separated from the supernatant by placement of the tubes on a magnetic stand and the beads were washed 5 times with 50 µl of screening buffer. Finally the washed beads were resuspended in 50 µl of screening buffer and denaturing sample SDS buffer was added to the supernatant, the washings, and the tubes containing the beads (elution). The samples were heated at 95°C for 5 min and loaded on 10% polyacrylamide SDS gel. After separation by electrophoresis, the gels were stained with coomassie or silver nitrate (Silver staining kit -protein Plus One Tm, Amersham) and the intensity of the bands was quantified using ImageJ software.

9. Hsp70 ATPase activity measures

Enzymatic activity measurements were performed according to previously described protocols.[6] Recombinant Hsp70- NBC1-18367 (Novus Biologicals) and recombinant Hsp40 (Novus Biologicals) were diluted on ATPase assay buffer, (0.017% Triton X-100, 100 mM Tris–HCl, 20 mM KCl, and 6 mM $MgCl_2$, pH 7.4). 14 µl of enzyme mix were pipetted in each well of a 96 well plate and 1 µl of a 100 mM solution of compound or DMSO was added to each well. After 15 min incubation at room temperature, 10 µl of 2.5 mM ATP was added to each well, to start the enzymatic activity measure. The reaction took place in a total final volume of 25 µL, 1 µM Hsp70, 1µM Hsp40 and 1 mM ATP. The plate was then incubated at 37°C for 3 hours. After incubation, 80 µL of Malachite Green reactive freshly prepared was added to each well of the plate for 1 min and the reaction was stopped by addition of 34% sodium citrate. The 600 nm absorbance was measured on a Spectramax M5 spectrophotometer (Molecular Devices) and the values obtained were corrected by background

[6] M.G. Rowlands, Y.M. Newbatt, C. Prodromou, L.H. Pearl, P. Workman, W. Aherne. *Anal. Biochem.* 2004, **327**, 176–183

substraction. As a control of Hsp70's ATPase activity, the kinetics of ATP hydrolysis was measure to be 733 μM/h/μg of protein, consistent with previous study.[6]

Malachita Green Hsp70 ATPase assay. ATP hydrolysis using Hsp70, Hsp40 (1 μM), ATP (1 mM)

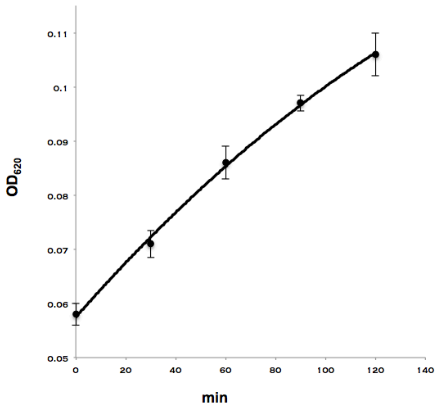

10. Pull down of Hsp70 from crude cell extracts

HEK293-T cells were grown on DMEM complemented with 10% Calf Fetal Serum (Sigma), 1% PenStrep, 1% non-essential amino acids, 1% Glutamine.

When the cells reached confluence after 48-72 h culture at 37° C, 5% CO_2, the monolayer from a T75 cell culture (10^6 cells approx.) flask was carefully washed with cold, sterile PBS and finally 1 ml of cold lysis buffer was added, 20 mM Tris-HCl pH 8, 137 mM NaCl, 10 % glycerol, 1% Triton X100, 2 mM EDTA, 1X HALT Protease inhibitors (Pierce). The flask was gently agitated for 5 min in ice and the cell monolayer scrapped from the flask and recovered in a mini centrifuge tube. The recovered cells were subjected to ultra sonication (three cycles of 30 seconds, on ice) and the protein lysate was centrifuged for 30 min at 14000 rpm, to eliminate cell membranes and cell debris. The supernatant was transferred to a fresh tube, and the soluble cytosolic proteins were quantified by Bradford Western blot immune detection.

10 μg of crude cell extract were incubated with functionalized SA coated beads Dynal (see Pulldown experiments). After one hour incubation with gentle agitation at room temperature in screening buffer complemented with protease inhibitors, the supernatant was recovered in a fresh tube and the beads washed five times with 50 μl of screening buffer. Finally the beads were resuspended in 50 μl of screening buffer and SDS sample buffer was added to all supernatants and bead containing tubes. The samples were heat denatured at 95° C for 5 min and analyzed on 12% SDS-PAGE (Invitrogen NuPAGE).   Alternatively, beads were submitted for tryptic digest and mass spectrometric analysis.

The polyacrilamide gel, containing the crude cell extract proteins after pull down separation were transferred to PVDF membranes (Invitrogen I-blot system) for western blot analysis. The western blot membranes were blocked (5% BSA or 5% non fat dry milk in TBS-T) and stored at -20°C.

mAb anti Hsp70 /72 , N6F3-6.410 (Stressgen), anti Hsp90, SPA-846 (Stressgen), pAb to carbonic anhydrase 2, R1069B (Acris Antibodies GmbH) were diluted as recommended by the manufacturer for western blot applications in TBS-T- 5% BSA.

The corresponding secondary antibodies HRP conjugated were purchased from Pierce Thermo Scientific. After incubation with each antibody, the western blot membranes were washed with TBS-T buffer, and the presence of antibodies was detected by chemical luminescence (Amersham ECL +).

The images were captured using a CCD camera placed in a dark room (Fusion FX7, Vilber Lourmat). The intensity of bands for quantitative purposes was done on ImageJ software.
